# Trends in body mass index for people with and without HIV: Pooled analysis of nationally-representative health surveys from 10 countries and 173,800 adults in Africa

**DOI:** 10.1371/journal.pgph.0003640

**Published:** 2024-09-17

**Authors:** Rodrigo M. Carrillo-Larco, Caroline A. Bulstra, Jennifer Manne-Goehler, Mark J. Siedner, Leslie C. M. Johnson, Vincent C. Marconi, Michael H. Chung, Willem Daniel Francois Venter, Erica Kocher, Samanta Lalla-Edward, Nomathemba C. Chandiwana, Jacob K. Kariuki, Mohammed K. Ali

**Affiliations:** 1 Hubert Department of Global Health, Rollins School of Public Health, Emory University, Atlanta, Georgia, United States of America; 2 Department of Global Health and Population, Health Systems Innovation Lab, Harvard T.H. Chan School of Public Health, Harvard University, Boston, Massachusetts, United States of America; 3 Heidelberg Institute of Global Health, Heidelberg University Hospital, Heidelberg, Germany; 4 Division of Infectious Diseases, Brigham and Women’s Hospital, Harvard Medical School, Boston, Massachusetts, United States of America; 5 Medical Practice Evaluation Center, Massachusetts General Hospital, Harvard Medical School, Boston, Massachusetts, United States of America; 6 Division of Infectious Diseases, Massachusetts General Hospital, Harvard Medical School, Boston, Massachusetts, United States of America; 7 Clinical Research Department, Africa Health Research Institute, KwaZulu-Natal, South Africa; 8 Department of Family and Preventive Medicine, Emory School of Medicine, Emory University, Atlanta, Georgia, United States of America; 9 Division of Infectious Diseases, Emory University School of Medicine, Emory University, Atlanta, Georgia, United States of America; 10 Department of Epidemiology, Rollins School of Public Health, Emory University, Atlanta, Georgia, United States of America; 11 Faculty of Health Sciences, Ezintsha, University of the Witwatersrand, Johannesburg, South Africa; 12 Faculty of Health Sciences, Department of Public Health Medicine, School of Health Systems and Public Health, University of Pretoria, Pretoria, South Africa; 13 Nutrition and Health Sciences Program, Laney Graduate School, Emory University, Atlanta, Georgia, United States of America; 14 Nell Hodgson Woodruff School of Nursing, Emory University, Atlanta, Georgia, United States of America; University of Modena and Reggio Emilia: Universita degli Studi di Modena e Reggio Emilia, ITALY

## Abstract

It remains unclear if and how body mass index (BMI) levels have changed over time in HIV endemic regions. We described trends in mean BMI and prevalence of overweight between 2003–2019 in 10 countries in Africa including people living with (PLWH) and without (PLWoH) HIV. We pooled Demographic and Health Surveys (DHS) from countries where ≥2 surveys >4 years apart were available with height/weight measurements and HIV tests. HIV status was ascertained with a finger-prick dried blood spot (DBS) specimen tested in a laboratory. The DBS is taken as part of the regular DHS procedures. We summarized age and socioeconomic status standardized sex-specific mean BMI (kg/m^2^) and prevalence of overweight (BMI ≥25 kg/m^2^) by HIV status. We fitted country-level meta-regressions to ascertain if changes in ART coverage were correlated with changes in BMI. Before 2011, women LWH (22.9 [95% CI: 22.2–23.6]) and LWoH (22.6 [95% CI: 22.3–22.8]) had similar mean BMI. Over time, mean BMI increased more in women LWH (+0.8 [95% CI: 0.7–0.8] BMI units) than LWoH (+0.2 [95% CI: 0.2–0.3]). Before 2013, the mean BMI was similar between men LWH (21.1 (95% CI: 20.3–21.9)) and LWoH (20.8 (95% CI: 20.6–21.1)). Over time, mean BMI increased more in men LWoH (+0.3 [95% CI: 0.3–0.3]) than LWH (+0.1 [95% CI: 0.1–0.1]). The same profile was observed for prevalence of overweight. ART coverage was not strongly associated with BMI changes. Mean BMI and prevalence of overweight were similar in PLWH and PLWoH, yet in some cases the estimates for PWLH were on track to catch up with those for PLWoH. BMI monitoring programs are warranted in PLWH to address the rising BMI trends.

## Introduction

Before antiretroviral treatments (ART) for HIV became available, HIV was associated with weight loss and wasting. The advent of ART and large-scale efforts to diagnose HIV early and to swiftly provide ART may have spared people living with HIV (PLWH) from experiencing significant loss of weight due to the impact of opportunistic illnesses, malabsorption, concomitant wasting, and the impact of inflammation-induced raised basal metabolic rate [1]. Increases in mean body mass index (BMI; kg/m^2^) and prevalence of overweight in non-HIV populations have been extensively characterized globally over the past four decades [2,3], and these increases have paralleled increases in other cardiometabolic conditions such as hypertension [4] and diabetes [5]. Similarly, efforts to study cardiometabolic risk factors in PLWH, including high BMI, have shown some signals of increasing weight and cardiometabolic conditions in this important subgroup. However, most of these studies were conducted almost a decade ago [6] and have generally included different subpopulations (e.g., community and clinical samples) and representation (e.g., subnational health surveys). To date, there have not been nationally-representative reports comparing trends in BMI and weight status in PLWH and people living without HIV (PLWoH) at the country level [7].

For public health officials, policymakers, and non-governmental organizations, the limited characterization of the BMI distribution in PLWH and PLWoH at the national level impedes monitoring efforts, development of clinical guidelines, and formulation of policies to address weight-related consequences of high BMI such as cardiometabolic diseases in PLWH. Community-based studies or those conducted in one or a few clinics may be too biased to inform population-wide interventions and programs.

We pooled nationally-representative data from 10 African countries with at least two survey timepoints four years apart including more than 173,800 people, to describe mean BMI and prevalence of overweight in PLWH and PLWoH over the past two decades in Africa, a region experiencing the largest burden of HIV [8]. Further, we examined if changes in national ART coverage were associated with BMI changes.

## Methods

### Study design and data sources

We obtained data from Demographic and Health Surveys (DHS) [9]. DHS has regularly conducted population-based health surveys among a nationally-representative sample of women (15–49 years) and men (15–59 years) in >90 low- and middle-income countries worldwide [9]. The DHS are multipurpose surveys studying topics such as maternal, reproductive, and child health, as well as non-communicable diseases. Because the DHS were originally designed to study maternal and child health, they only started including men since the early 2000s. Consequently, we report findings separately for men and women because not all DHS had collected data for both sex.

### Study population–countries

**Fig 1:**
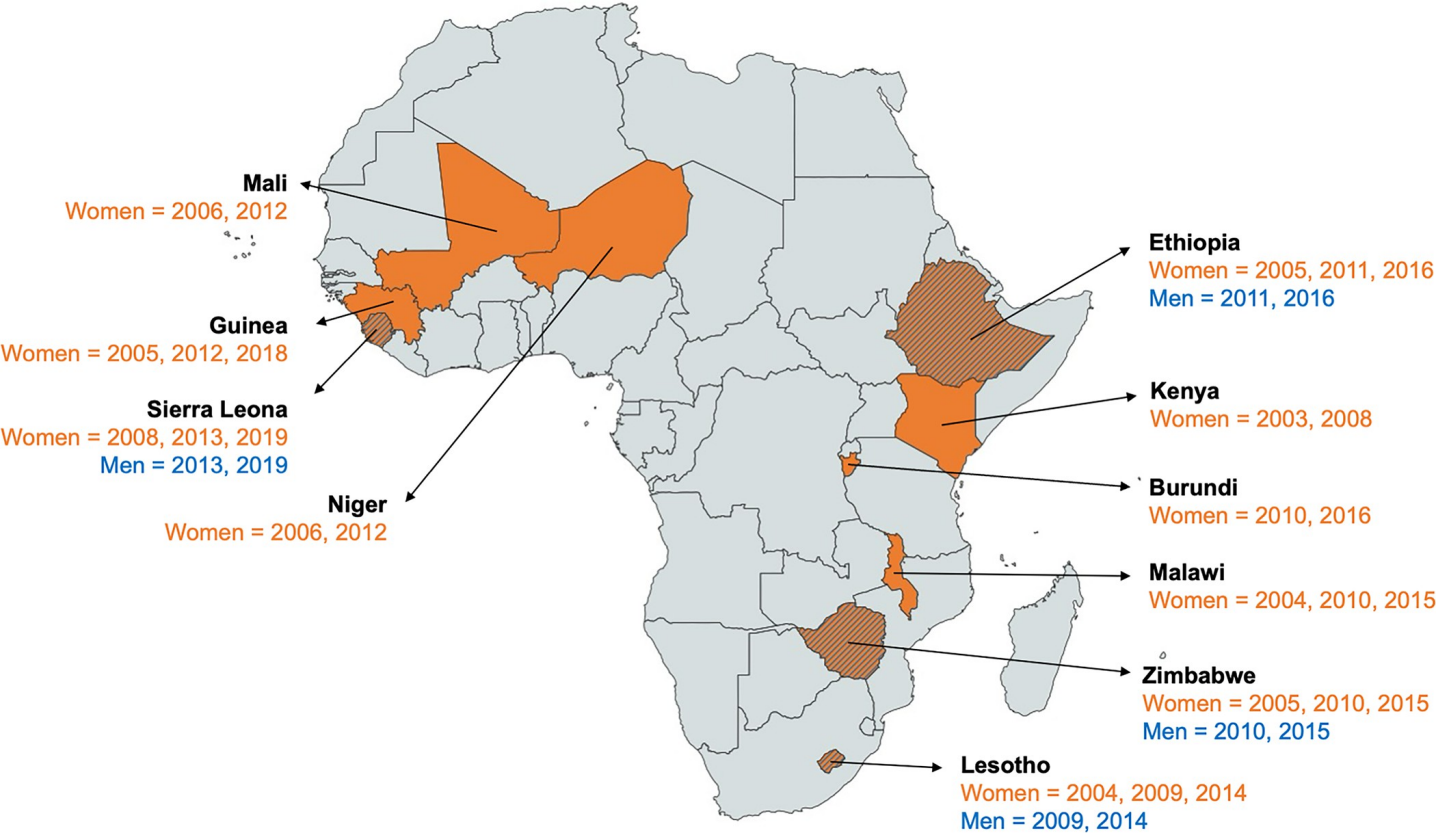
Map of sub-Saharan Africa showing the countries, surveys, and years included in the analysis. Countries in solid color contributed data for women only. The years refer to when the Demographic and Health Survey (DHS) was conducted in each country. The map was created by authors using lisenced ArcGIS Pro desktop software version 10.5 (http://www.arcgis.com/). Sources: Country boundaries are provided under an open license (CC-BY) by GADM (https://gadm.org/). Data obtained through the Demographic and Health Surveys (DHS) Program (https://dhsprogram.com/).

We selected countries with at least two DHS survey rounds and those in which weight and height were measured and HIV blood tests were collected. We included countries in Africa because the burden of HIV is greatest in this region, particularly in Sub-Saharan Africa [8]. We pooled 26 DHS from 10 countries for women; for men, we pooled eight DHS from four unique countries (Table A in S1 Text and Fig 1).

### Study population–analytical sample

We excluded observations with implausible values in weight (<12 kg (n = 6) or >300 kg (n = 1)), height (<100 cm (n = 114) or >250 cm (n = 0)), and BMI (<10 kg/m^2^ (n = 0) or >80 kg/m^2^ (n = 0)) [10]; we also excluded pregnant women (self-reported; n = 26,711).

We conducted a complete-case analysis with respect to the sampling variables (primary sampling unit, stratum and sampling weights), age and sex, anthropometrics, and HIV test (Table B in S1 Text). There were no substantial differences between individuals included and excluded from the analytical dataset (Table C in S1 Text).

### Variables–exposure

BMI (kg/m^2^) was calculated from measured weight and height ascertained during the fieldwork by trained personnel using standard tools and following DHS protocols across countries [9]. Overweight was defined as BMI ≥25 kg/m^2^. We focused on overweight (BMI ≥25 kg/m^2^) rather than obesity (BMI ≥30 kg/m^2^) to maximize the number of observations, as very fewer people would meet the criterion for obesity than for overweight [2] and the estimates would be impresice.

### Variables–outcome

HIV status was ascertained with a finger-prick dried blood spot (DBS) specimen tested in a laboratory. The DBS is taken as part of the regular DHS procedures. The exact testing algorithm in each survey and the laboratory tests they used are shown in Table A in S1 Text. In brief, and while there were slight variations per survey, the testing algorithm often involved three tests. Negative results in the first test were considered negative, whereas positive results were tested again with a different test. If positive with both tests, then the results were positive; however, if there were discrepancies between the two tests, a third test was used. HIV status was defined as negative (no) and positive (yes).

### Variables–sociodemographic and ART coverage

Sex and age were self-reported during DHS data collection. We used the socioeconomic status (SES) variable provided by the DHS in five groups (poorest, poor, middle, richer, and richest) [11].

We used ART coverage (%) at the country level for the same year when the DHS was conducted. ART coverage data was extracted from World Health Organization statistics [12].

### Statistical analysis

We described sex-specific mean BMI and prevalence (%) of overweight disaggregated by HIV status overall and by country. Unless otherwise stated, means and prevalence estimates were computed accounting for the sampling design of each DHS. Means and prevalence estimates were reported with 95% Confidence Intervals (95% CI). The mean and prevalence estimates were age-SES standardized in reference to a standard population we built using the full dataset; in so doing, we standardized our results, with respect to the age and SES distribution, to a standard population across the countries included in the analysis and throughout the observation period [13]. Details about the age-SES standardization are available in Table D in S1 Text. Crude mean and prevalence estimates (i.e., not age-SES standardized) are also available in S1 Text.

First, we grouped the earliest and latest surveys and computed age-SES standardized sex-specific mean BMI and prevalence of overweight for each timepoint (Table E in S1 Text). For women, earlier surveys included Burundi 2010, Ethiopia 2005, Guinea 2005, Kenya 2003, Lesotho 2004, Malawi 2004, Mali 2006, Niger 2006, Sierra Leone 2008, and Zimbabwe 2005; conversely, the latest surveys included Burundi 2016, Ethiopia 2016, Guinea 2018, Kenya 2008, Lesotho 2014, Malawi 2015, Mali 2012, Niger 2012, Sierra Leone 2019, and Zimbabwe 2015. Because 2011 roughly split the same number of surveys before and after, for women the earliest and latest periods are hereafter referred to as 2011 or before and 2012 and later. For men, the earliest surveys included Ethiopia, 2011, Lesotho 2009, Sierra Leona 2013, and Zimbabwe 2015; conversely, the latest surveys included Ethiopia 2016, Lesotho 2014, Sierra Leona 2019, and Zimbabwe 2015. Because 2014 roughly split the same number of surveys before and after, for men the earliest and latest periods are hereafter referred to as 2013 or before and 2014 or after. These mean and prevalence estimates were age-SES standardized and reported separately by HIV status.

Second, we computed age-SES standardized sex-specific mean BMI as well as the prevalence of overweight by country throughout the observation period and results were reported separately by HIV status.

Third, for PLWH we plotted the difference in mean BMI between the latest and earliest periods (y-axis) versus the difference in ART coverage between the latest and earliest periods (x-axis). In addition, we conducted a study-level meta-regression with these two variables: difference in mean BMI was the dependent variable and difference in ART coverage was the independent variable. We fitted a crude model only including the dependent and independent variables. We also fitted an adjusted model including region and income group to which each country belonged (Table A in S1 Text). Results for the meta-regressions were presented as β coefficients with the 95% CI, and a p-value <0.05 was considered statistically significant. The meta-regression used the crude mean BMI (not age-SES standardized).

Fourth, we plotted on the y-axis a difference in difference: difference in mean BMI between the latest and earliest periods in PLWoH *minus* difference in mean BMI between the latest and earliest periods in PLWH. On the x-axis, we plotted the difference in ART coverage between the latest and earliest periods. In addition, we conducted a study-level meta-regression with these two variables as the dependent (difference in difference) and independent (difference in ART coverage) variables. We fitted a crude model only including the dependent and independent variables. We also fitted an adjusted model including region and income group to which each country belonged (Table A in S1 Text). Results for the meta-regressions were presented as β coefficients with the 95% CI, and a p-value <0.05 was considered statistically significant. The meta-regression used the crude mean estimates (not age-SES standardized).

Equivalent meta-regressions described above in the third and fourth steps were conducted for the prevalence of overweight; that is, the mean BMI was replaced by the prevalence of overweight and the other procedures were the same.

Fifth, to further explore the role of SES in the distribution of BMI and overweight in PLWH and PLWoH, we computed the mean BMI and prevalence of overweight by DHS, HIV status, sex, and SES quintile. Owing to the small absolute number of observations after such thin stratification, these SES-specific mean and prevalence estimates were not age standardized. In addition and including the full study population, we fitted individual-level multilevel linear (outcome: BMI) and logistic (outcome: overweight status) regressions where the independent variable was HIV status. We fitted regression models adjusted by age, sex, and SES; also, we fitted regression models adjusted by age and sex, and stratified by SES. We used the *mixed* and *meqrlogit* functions in Stata for multilevel linear and logistic regressions, respectively. We specified a random intercepted for each DHS and assumed an unstructured covariance matrix. The regression models were fitted overall and stratified by period (earliest and latest surveys).

Data management and analysis were conducted in R (4.3.0). The regressions were conducted in Stata (17.0 SE-Standard Edition, College Station, Texas, US).

### Ethics

All analyses were performed on publicly available de-identified DHS data.

### Role of the funding source

The funder had no role in study design or analysis plan, neither in the presentation of the results or conclusions. The opinions in this manuscript belong to the authors alone, and do not necessarily represent those of the institutions to which the authors belong.

## Results

### Study population

The analysis included 10 unique countries for women (Burundi, Ethiopia, Guinea, Malawi, Mali, Niger, Kenya, Lesotho, Sierra Leone, and Zimbabwe) prior to (n = 33,563) and after 2012 (55,040). The analysis included four unique countries for men (Ethiopia, Lesotho, Sierra Leone, and Zimbabwe) with data prior to (n = 24,827) and after 2014 (n = 23,653).

The included surveys were conducted between 2003 and 2019 (Table 1). The unweighted description of the study sample by DHS is available in Table 1 and Table F in S1 Text. The largest DHS datasets were from Ethiopia (23,436 and 21,442 in 2011 and 2016, respectively) and the smallest DHS dataset was from Malawi (2,190 in 2004). The absolute number of PLWH ranged between 24 (Niger, 2012) and 2,315 (Zimbabwe, 2015). For DHS having both men and women, the sex distribution was roughly the same except in Lesotho 2009 (45% men) as well as Zimbabwe in 2010 and 2015 (45% and 46% men, respectively). The mean age ranged between 30 and 33 years. The unweighted distribution of SES is available in Table G in S1 Text, showing differences between PLWH and PLWoH; notably, most PLWH were in the *richer* or *richest* quintiles and in most countries this was particularly so in the earliest surveys (hence the age-SES standardization of the mean and prevalence estimates).

**Table 1:** Unweighted description of the study sample by survey.

	Age (years)	Sex	BMI (kg/m^2^)	HIV test+
**Burundi 2010 (N = 3344)**	Mean (SD): 30.0 (9.13)	Men: 0 (0%)	Mean (SD): 21.5 (3.68)	No: 3253 (97.3%)
	Median [Min, Max]: 28.0 [18.0, 49.0]	Women: 3344 (100%)	Median [Min, Max]: 20.9 [10.8, 62.1]	Yes: 91 (2.7%)
**Burundi 2016 (N = 6582)**	Mean (SD): 30.3 (8.86)	Men: 0 (0%)	Mean (SD): 21.2 (3.32)	No: 6468 (98.3%)
	Median [Min, Max]: 29.0 [18.0, 49.0]	Women: 6582 (100%)	Median [Min, Max]: 20.7 [10.8, 54.8]	Yes: 114 (1.7%)
**Ethiopia 2005 (N = 4631)**	Mean (SD): 29.9 (8.88)	Men: 0 (0%)	Mean (SD): 20.5 (3.21)	No: 4501 (97.2%)
	Median [Min, Max]: 28.0 [18.0, 49.0]	Women: 4631 (100%)	Median [Min, Max]: 20.0 [10.1, 66.6]	Yes: 130 (2.8%)
**Ethiopia 2011 (N = 23436)**	Mean (SD): 31.2 (9.91)	Men: 11345 (48.4%)	Mean (SD): 20.1 (3.08)	No: 22919 (97.8%)
	Median [Min, Max]: 30.0 [18.0, 59.0]	Women: 12091 (51.6%)	Median [Min, Max]: 19.6 [10.5, 63.8]	Yes: 517 (2.2%)
**Ethiopia 2016 (N = 21442)**	Mean (SD): 31.4 (9.82)	Men: 9925 (46.3%)	Mean (SD): 20.6 (3.39)	No: 21064 (98.2%)
	Median [Min, Max]: 30.0 [18.0, 59.0]	Women: 11517 (53.7%)	Median [Min, Max]: 19.9 [11.3, 65.8]	Yes: 378 (1.8%)
**Guinea 2005 (N = 2968)**	Mean (SD): 31.7 (9.05)	Men: 0 (0%)	Mean (SD): 21.8 (3.47)	No: 2906 (97.9%)
	Median [Min, Max]: 30.0 [18.0, 49.0]	Women: 2968 (100%)	Median [Min, Max]: 21.2 [12.9, 47.8]	Yes: 62 (2.1%)
**Guinea 2012 (N = 3586)**	Mean (SD): 30.7 (9.08)	Men: 0 (0%)	Mean (SD): 22.6 (4.12)	No: 3496 (97.5%)
	Median [Min, Max]: 30.0 [18.0, 49.0]	Women: 3586 (100%)	Median [Min, Max]: 21.8 [12.9, 67.9]	Yes: 90 (2.5%)
**Guinea 2018 (N = 4033)**	Mean (SD): 31.1 (9.16)	Men: 0 (0%)	Mean (SD): 23.7 (4.92)	No: 3957 (98.1%)
	Median [Min, Max]: 30.0 [18.0, 49.0]	Women: 4033 (100%)	Median [Min, Max]: 22.7 [11.3, 75.7]	Yes: 76 (1.9%)
**Kenya 2003 (N = 2551)**	Mean (SD): 30.1 (8.72)	Men: 0 (0%)	Mean (SD): 23.1 (4.41)	No: 2310 (90.6%)
	Median [Min, Max]: 29.0 [18.0, 49.0]	Women: 2551 (100%)	Median [Min, Max]: 22.2 [12.9, 60.1]	Yes: 241 (9.4%)
**Kenya 2008 (N = 3077)**	Mean (SD): 30.5 (9.03)	Men: 0 (0%)	Mean (SD): 23.3 (4.79)	No: 2796 (90.9%)
	Median [Min, Max]: 29.0 [18.0, 49.0]	Women: 3077 (100%)	Median [Min, Max]: 22.4 [10.6, 75.0]	Yes: 281 (9.1%)
**Lesotho 2004 (N = 2352)**	Mean (SD): 30.6 (9.35)	Men: 0 (0%)	Mean (SD): 25.3 (5.35)	No: 1636 (69.6%)
	Median [Min, Max]: 29.0 [18.0, 49.0]	Women: 2352 (100%)	Median [Min, Max]: 24.2 [11.2, 53.7]	Yes: 716 (30.4%)
**Lesotho 2009 (N = 5676)**	Mean (SD): 31.2 (10.4)	Men: 2575 (45.4%)	Mean (SD): 23.6 (5.16)	No: 4212 (74.2%)
	Median [Min, Max]: 29.0 [18.0, 59.0]	Women: 3101 (54.6%)	Median [Min, Max]: 22.3 [12.3, 60.5]	Yes: 1464 (25.8%)
**Lesotho 2014 (N = 5052)**	Mean (SD): 31.5 (10.4)	Men: 2352 (46.6%)	Mean (SD): 24.2 (5.27)	No: 3674 (72.7%)
	Median [Min, Max]: 30.0 [18.0, 59.0]	Women: 2700 (53.4%)	Median [Min, Max]: 22.7 [14.5, 63.7]	Yes: 1378 (27.3%)
**Malawi 2004 (N = 2190)**	Mean (SD): 30.0 (8.75)	Men: 0 (0%)	Mean (SD): 22.2 (3.39)	No: 1819 (83.1%)
	Median [Min, Max]: 28.0 [18.0, 49.0]	Women: 2190 (100%)	Median [Min, Max]: 21.6 [12.3, 56.7]	Yes: 371 (16.9%)
**Malawi 2010 (N = 5597)**	Mean (SD): 30.6 (8.70)	Men: 0 (0%)	Mean (SD): 22.6 (3.73)	No: 4801 (85.8%)
	Median [Min, Max]: 29.0 [18.0, 49.0]	Women: 5597 (100%)	Median [Min, Max]: 22.0 [10.4, 76.1]	Yes: 796 (14.2%)
**Malawi 2015 (N = 6167)**	Mean (SD): 30.2 (8.62)	Men: 0 (0%)	Mean (SD): 23.2 (4.20)	No: 5384 (87.3%)
	Median [Min, Max]: 29.0 [18.0, 49.0]	Women: 6167 (100%)	Median [Min, Max]: 22.3 [10.2, 69.4]	Yes: 783 (12.7%)
** Mali 2006 (N = 3492)**	Mean (SD): 30.8 (8.97)	Men: 0 (0%)	Mean (SD): 22.6 (4.19)	No: 3436 (98.4%)
	Median [Min, Max]: 30.0 [18.0, 49.0]	Women: 3492 (100%)	Median [Min, Max]: 21.7 [11.9, 56.4]	Yes: 56 (1.6%)
** Mali 2012 (N = 4016)**	Mean (SD): 30.4 (8.47)	Men: 0 (0%)	Mean (SD): 22.8 (4.60)	No: 3966 (98.8%)
	Median [Min, Max]: 30.0 [18.0, 49.0]	Women: 4016 (100%)	Median [Min, Max]: 21.8 [12.6, 71.1]	Yes: 50 (1.2%)
**Niger 2006 (N = 3297)**	Mean (SD): 30.7 (8.64)	Men: 0 (0%)	Mean (SD): 22.2 (4.14)	No: 3263 (99.0%)
	Median [Min, Max]: 30.0 [18.0, 49.0]	Women: 3297 (100%)	Median [Min, Max]: 21.3 [13.5, 51.8]	Yes: 34 (1.0%)
**Niger 2012 (N = 3869)**	Mean (SD): 30.6 (8.37)	Men: 0 (0%)	Mean (SD): 22.7 (4.49)	No: 3845 (99.4%)
	Median [Min, Max]: 30.0 [18.0, 49.0]	Women: 3869 (100%)	Median [Min, Max]: 21.9 [13.8, 77.2]	Yes: 24 (0.6%)
**Sierra Leone 2008 (N = 2830)**	Mean (SD): 30.8 (8.30)	Men: 0 (0%)	Mean (SD): 23.9 (5.75)	No: 2773 (98.0%)
	Median [Min, Max]: 30.0 [18.0, 49.0]	Women: 2830 (100%)	Median [Min, Max]: 22.9 [10.0, 75.4]	Yes: 57 (2.0%)
**Sierra Leone 2013 (N = 11905)**	Mean (SD): 32.2 (10.2)	Men: 5790 (48.6%)	Mean (SD): 22.4 (3.87)	No: 11713 (98.4%)
	Median [Min, Max]: 31.0 [18.0, 59.0]	Women: 6115 (51.4%)	Median [Min, Max]: 21.7 [10.6, 64.8]	Yes: 192 (1.6%)
**Sierra Leone 2019 (N = 10972)**	Mean (SD): 32.7 (10.5)	Men: 5177 (47.2%)	Mean (SD): 22.9 (3.84)	No: 10765 (98.1%)
	Median [Min, Max]: 31.0 [18.0, 59.0]	Women: 5795 (52.8%)	Median [Min, Max]: 22.1 [14.1, 62.8]	Yes: 207 (1.9%)
**Zimbabwe 2005 (N = 5908)**	Mean (SD): 29.9 (8.89)	Men: 0 (0%)	Mean (SD): 23.4 (4.28)	No: 4498 (76.1%)
	Median [Min, Max]: 28.0 [18.0, 49.0]	Women: 5908 (100%)	Median [Min, Max]: 22.5 [11.0, 62.0]	Yes: 1410 (23.9%)
**Zimbabwe 2010 (N = 11357)**	Mean (SD): 30.5 (9.26)	Men: 5117 (45.1%)	Mean (SD): 22.9 (4.33)	No: 9235 (81.3%)
	Median [Min, Max]: 29.0 [18.0, 54.0]	Women: 6240 (54.9%)	Median [Min, Max]: 21.9 [13.2, 62.9]	Yes: 2122 (18.7%)
**Zsimbabwe 2015 (N = 13483)**	Mean (SD): 31.1 (9.20)	Men: 6199 (46.0%)	Mean (SD): 23.6 (4.69)	No: 11168 (82.8%)
	Median [Min, Max]: 30.0 [18.0, 54.0]	Women: 7284 (54.0%)	Median [Min, Max]: 22.5 [11.4, 78.0]	Yes: 2315 (17.2%)

An expanded version of this table, depicting additional variables such as weight, height, and the proportion of overweight individuals, is available as Table F in S1 Text. BMI = body mass index (kg/m^2^).

### Age-SES standardized sex-specific mean body mass index and prevalence of overweight trends

**Fig 2:**
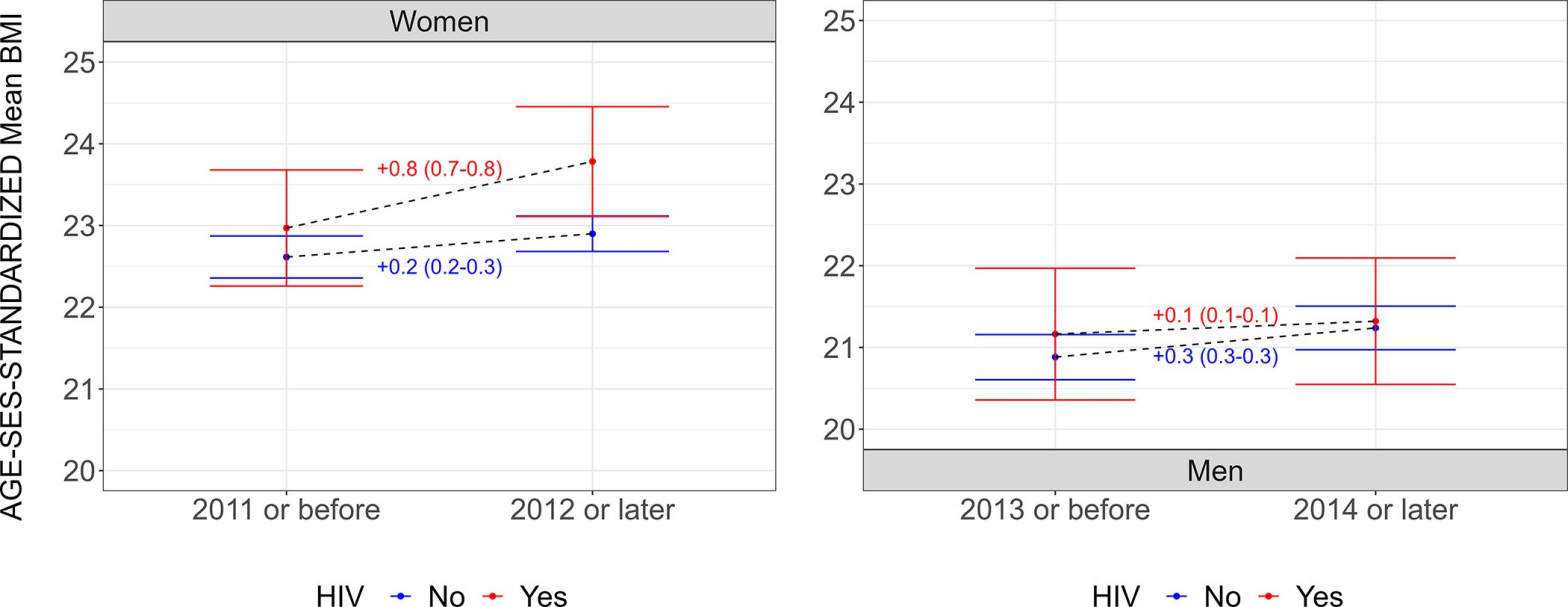
Age-SES standardized mean body mass index (BMI; kg/m^2^) by period, sex, and HIV status. Results account for the complex survey design of each DHS. Results without age-SES standardization are available in Table H in S1 Text. Underlying results are available in Table I in S1 Text. A comparison between the age-standardized means versus the crude means is shown in Fig H in S1 Text. For a list of DHS included in the earliest (before 2011 for women and before 2014 for men) and latest (after 2011 for women and after 2011 for men) periods, please refer to Table E in S1 Text. SES: socioeconomic status.

Before 2012, women LWH and LWoH had virtually the same mean BMI: 22.9 (95% CI: 22.2–23.6) and 22.6 (95% CI: 22.3–22.8), respectively (Fig 2). Over time, mean BMI increased more in women LWH (+0.8 (95% CI: 0.7–0.8) BMI units) than it did in women LWoH (+0.2 (95% CI: 0.2–0.3) BMI units). Before 2014, mean BMI was similar between men LWH and LWoH: 21.1 (95% CI: 20.3–21.9) and 20.8 (95% CI: 20.6–21.1), respectively. Over time, mean BMI increased more in men LWoH (+0.3 (95% CI: 0.3–0.3) BMI units) than it did in men LWH (+0.1 (95% CI: 0.1–0.1) BMI units). Overall, the same profile was observed regarding the prevalence of overweight: no substantial differences between PLWH and PWLoH in the earliest and latest periods, and the prevalence of overweight increased more in women LWH as well as in men LWoH (Fig A in S1 Text).

### Age-SES standardized mean body mass index and prevalence of overweight by country and sex

**Fig 3:**
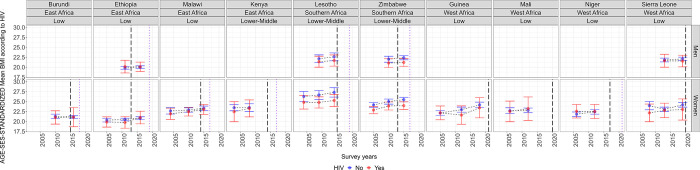
Age-SES standardized mean body mass index (BMI; kg/m^2^) stratified by HIV status, sex and survey. Results account for the complex survey design of each DHS. The vertical black dashed line signals the year when antiretroviral treatment (ART) coverage reached 50% of the population. The vertical purple dotted line signals the year when the ART coverage reached 75% of the population. The crude results (i.e., not age-SES standardized) are available in Table L in S1 Text. The underlying results are available in Table M in S1 Text. SES: socioeconomic status.

Across countries, data timepoints, and for both men and women, we did not observe large differences between PLWH and PLWoH; that is, mean BMI and prevalence estimates were close between PLWH and PLWoH and the 95% CI overlapped (Fig 3 and Fig B in S1 Text). In some countries the estimates for PLWH have been catching up with those of PLWoH. For example, in Malawi the difference in mean BMI between women LWH and women LWoH was -0.7 (95% CI: -1.3; -0.1) in 2004, but it was -0.2 (95% CI: -0.9; 0.4) in 2015. Similarly, in Kenya mean BMI in women LWH was -0.9 (95% CI: -2.7; 0.8) lower than in women LWoH in 2003, but in 2014 such difference was -0.2 (95% CI: -1.3; 0.9).

### Antiretroviral treatment coverage and changes in mean body mass index and prevalence of overweight

**Fig 4:**
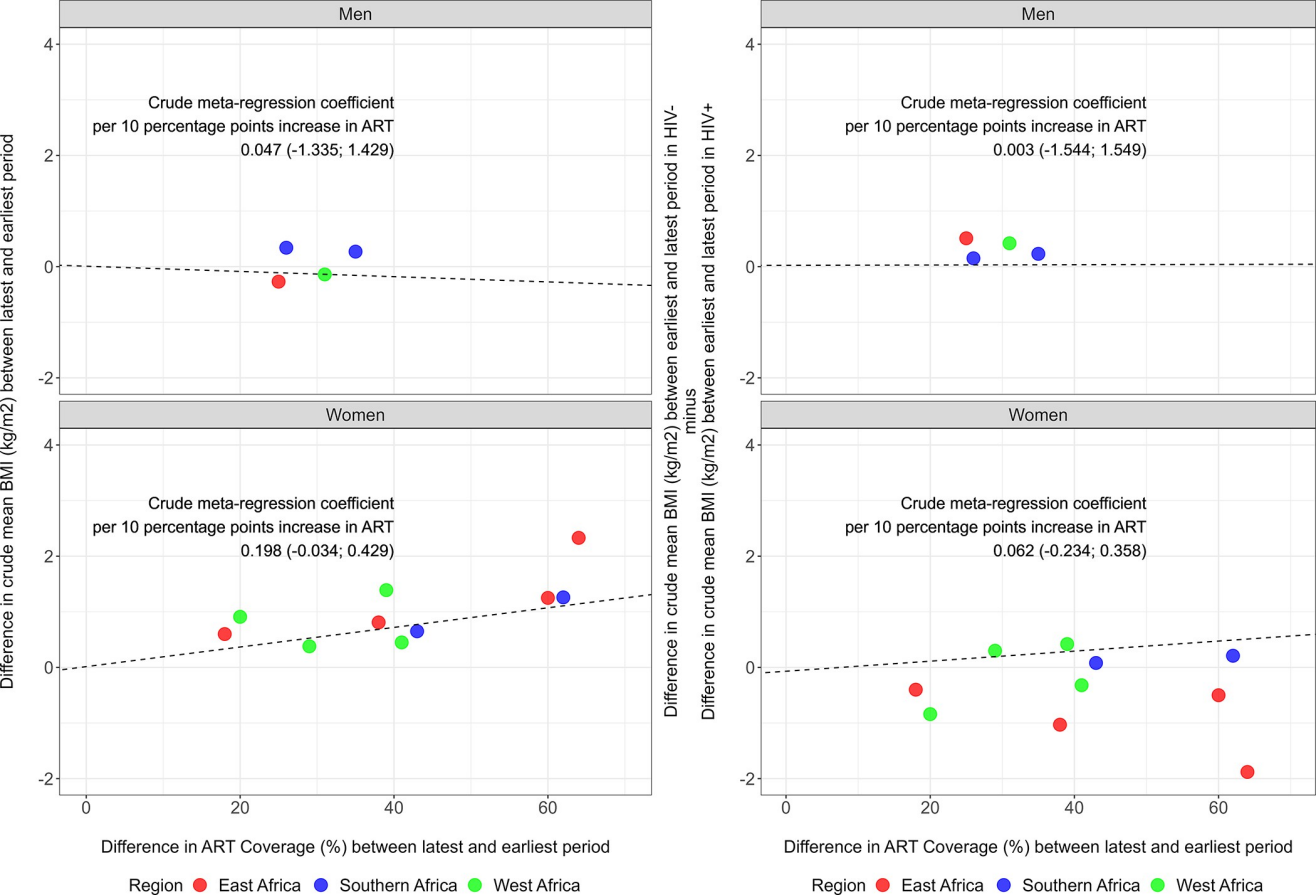
Meta-regression of mean body mass index (BMI; kg/m^2^), as well as difference in mean body mass index between people without and with HIV, on antiretroviral treatment coverage. Each dot is a DHS. Colors depict the regions. We plotted the difference in mean BMI between the latest and earliest periods (y-axis), versus the difference in ART coverage between the latest and earliest periods (x-axis). The exposure was ART coverage by 10 percentage points to make the regression coefficients meaningful, i.e., observed prevalence of ART coverage divided by 10. Both the outcome and the exposure were at the country level and in the same calendar year. The meta-regression used the crude mean BMI (not age standardized). This figure shows the coefficients for the crude meta-regression, for coefficients of the adjusted meta-regression model and the 95% confidence intervals, please refer to Table P in S1 Text.

In women and men, there was not a strong (i.e., not statistically significant) positive correlation between increase in country-level ART coverage and increases in BMI as well as in prevalence of overweight (Fig 4 and Fig C in S1 Text). Greater ART coverage was not strongly correlated with a narrowing gap between women LWoH and women LWH; conversely, in men, greater ART coverage seemed to be correlated with a widening gap between men LWoH and men LWH though the correlation was not strong.

### Mean body mass index and prevalence of overweight by country, sex, and SES

The non-age standardized mean BMI increased from the poorest to the richest quintiles across years, for both men and women as well as in PLWH and PLWoH (Table Q in S1 Text, Fig D in S1 Text, and Fig E in S1 Text). PLWH had, on average, -0.73 (95% CI: -0.79; -0.65) BMI units less than PLWoH, and this was independent of age, sex, and SES; similar estimates were observed in the period-specific regression models (Table S in S1 Text) and in the regression models stratified by SES quintiles (Table T in S1 Text).

The non-age standardized prevalence of overweight increased from the poorest to the richest quintiles across years, for both men and women as well as in PLWH and PLWoH (Table R in S1 Text, Fig F in S1 Text, and Fig G in S1 Text). PLWH were 31% (OR: 0.69 [95% CI: 0.66–0.73]) less likely to have overweight in comparison to PLWoH, independent of age, sex, and SES; similar findings were seen in the period-specific regression models (Table S in S1 Text) as well as in the regression models stratified by SES quintiles (Table T in S1 Text).

## Discussion

### Main findings

In this pooled analysis of DHS from 10 countries and more than 173,800 adults in West, East, and Southern Africa from 2003–2019, age-SES standardized mean BMI and prevalence of overweight increased for PLWH and PLWoH, and yet the increase was more perceptible in women LWH than in women LWoH. Notably, mean BMI and prevalence of overweight between PLWH and PLWoH were not substantially different in the earliest and latest periods. ART coverage was not strongly associated with increases in BMI and overweight prevalence in women and men. On average, PLWH, both women and men, had lower mean BMI and prevalence of overweight than PLWoH independent of age, sex and SES.

As our study shows, nationally-representative BMI distributions in PLWH and PLWoH between 2003 and 2019, these estimates are uniquely positioned to inform policies and public health priorities. This should motivate urgent policies to ensure healthy weight gain and cardiometabolic health in PLWH, particularly women. Our results also imply a public health concern, above and beyond the physiological effects of high BMI on individual patients. Our findings contextualize the growing burden of high BMI in PLWH and reinforce the notion that weight gain in PLWH deserves careful monitoring, to avoid multimorbidity and complications as PLWH age.

### Results in context

A 2012 systematic review reported that HIV-positive status was associated with lower BMI (standard mean difference -0.32 [95% CI: -0.45; -0.18]) [6]. We have now updated and advanced this evidence reporting that mean BMI and prevalence of overweight in PLWH has been catching up with that of PLWoH. The systematic review by Dillon *et al*. provided evidence regarding other cardiometabolic risk factors (e.g., lipids, glucose and blood pressure) and also compared PLWH according to ART status [6]. We leveraged DHS data which historically have not systematically collected information about other cardiometabolic risk factors; disaggregating the results for PLWH would have led to small absolute numbers and therefore unreliable estimates. In addition, information about ART status and viral load is not collected in DHS. National surveys collecting several cardiovascular risk factors and that oversample PLWH are warranted; alternatively, pooling consecutive surveys to maximize the absolute number of PLWH could be an efficient and timely solution. There is urgent need for national health surveys with data on cardiometabolic diseases and HIV. The fact that only 26 DHS from 10 countries had anthropometric measurements and HIV tests, shows there is a glaring lack of data on both cardiometabolic risk factors and HIV at the population level.

We did not document large differences in mean BMI and prevalence of overweight between PLWH and PLWoH. While unexpected, particularly in the early years, and considering the limitations of our study as well as those from the original data resource (DHS), there are several possible reasons to consider. First, we analyzed national household-based surveys; hence, PLWH who were very ill and with low BMI may not have participated or were not able to participate (e.g., hospitalized or bedridden). Second, our findings could have been influenced by survival bias, yet it is not possible to evaluate the magnitude or direction of such bias. For instance, PLWH who survived and participated in DHS could have been healthier and heavier, shifting the BMI distribution to the right; alternatively, PLWH could have survived but still had low weight shifting the BMI distribution to the left. Third, the distribution of socioeconomic status in PLWH could also explain our findings. In all the DHS analyzed here, PLWH were predominately in the *richer* or *richest* groups and evidence suggests that in low- and middle-income countries the burden of obesity is greatest in these demograhics [14,15]. Although we age-SES standardized our estimates, there could still be other SES features shifting the BMI distribution upwards for PLWH (i.e., residual confounding). Fourth, it is possible that there may have been differential rates of HIV incidence whereby people who had higher SES and weight became HIV positive, hence the higher means and prevalences observed in later periods. This is largely speculative and we cannot fully assess this hypothesis because our work described two timepoints individually. Fifth, differential HIV screening over time could have also played a role in explaining our findings. Early in the HIV pandemic testing started with symptomatic patients who, arguably, would have some degree of low weight or wasting; as the HIV pandemic progressed, new testing strategies were implemented and more resources became available, testing moved to the broader population finding asymptomatic individuals who may not have yet experienced significant weight loss. Finally, our findings are in line with previous community-based studies or studies enrolling PLWH from clinics, that have reported similar BMI levels between PLWH and PLWoH [14,16–18]. For example, a study with 153 PLWH in Ethiopia in 2005–2006 reported that the mean BMI was 21.2 (standard deviation = 3.0) [16]; estimates for the year 2006 in Ethiopia showed that the national mean BMI was 20.4 (95% CI: 19.8–21.0) and 19.9 (95% CI: 19.2–20.7) for women and men, respectively [3]. A study with 53,825 patients enrolled between 2004 and 2011 from HIV/AIDS care and treatment centrers in Tanzania reported that the median BMI was 22 (interquartile range: 20–25) [17]; in the same period, the national mean BMI in Tanzania remained within the same range (e.g., 23.3 [95% CI: 22.9–23.7] in women in 2008 and 22.1 [95% CI: 21.6–22.7] in men in 2008) [3]. A study with 247 women from an academic hospital in South Africa reported that the median BMI in PLWoH was virtually the same as in PLWH with preserved CD4 count (27.3 [interquartile range: 23.1–31.7] versus 27.8 [interquartile range: 23.3–32.3], respectively) [18]. Although this is not an exhaustive comparison, these reports do not suggest that the average BMI in PLWH was substantially different than in PLWoH.

The mean BMI and the prevalence of overweight have been increasing worldwide in the general population [2,3]. BMI increases have also been documented in many parts of Africa [3] and our results are largely in line with these estimates. General drivers of the obesity epidemic, namely sedentarism and unhealthy diet [15], could explain our findings. In addition to general risk factors for weight gain [15], there may be other, more specific drivers related to HIV infection [19], weight-inducing or weight-blunting effects of newer versus older ART regimes [20–23], aging of PLWH associated with greater access to life-saving ART, and possibly earlier diagnosis through large-scale programs like the U.S. President’s Emergency Plan for AIDS Relief (PEPFAR) program [24].

We saw weak evidence of seemingly different trends in men and women, including the results from the meta-regressions where in some instances there was a positive correlation for women but negative for men. The sex disparities in HIV-related outcomes, such as lower life expectancy in men compared to women after ART [25,26], are well documented. In this regard, survival bias could play a role, with healthier women who have better weight status surviving, thus reflecting the higher BMI increase in women LWH than in men LWH. Similarly, viral load suppression seems to be higher in women than in men in Sub-Saharan Africa [27], facilitating higher and faster weigh gain in women LWH. Our findings also parallel global estimates revealing that BMI has increased faster in women in many parts of Africa [2]. Nonetheless, these hypotheses deserve further exploration to identify the most important explanations, enabling interventions to prevent sex disparities in weight gain among PLWH.

### Strengths and limitations

We pooled nationally-representative health surveys in 10 countries in Africa with objective measurements of weight, height, and HIV. We studied countries with at least two survey timepoints four years apart. The DHS follow a consistent sampling strategy and measurement protocols across countries [9].

Notwithstanding, there are important limitations. First, there were fewer surveys with data for men. The DHS have been including men in recent years and future analyses will have access to more men data. For transparency, we have reported the results by sex, including confidence intervals and sample sizes. Second, we did not have information about the antiretrovirals that the survey participants were taking. Thus, we could not assess the correlates with specific treatment schemes. Similarly, we did not have data about viral load, and therefore could not dissect BMI levels in people with high versus undetectable viral load. Third, most DHS herein analyzed were conducted before 2018. Although there is no systematic repository of when Integrase Strand Transfer Inhibitors (INSTIs) began to be prescribed extensively by country, it must have been circa 2018 in most parts of Africa. Because most of the DHS herein analyzed were conducted before 2018, it was not possible to ascertain the ecological association between BMI and INSTI coverage. Our findings can therefore be considered pre-INSTI and future analyses should report on BMI trends following widespread use of INSTI. Evidence have shown that taking INSTIs may be associated with cardiometabolic risk factors including hypertension [21,28–30], diabetes [28,31,32], and weight gain [20–23], yet weight status should also be evaluated at the population level and our work provides baseline results for future estimates. Fourth, we did not study other anthropometric indicators such as waist circumference which may be more strongly associated with cardiometabolic diseases, or other markers to characterize lipodystrophy. Data for waist circumference, and other anthropometric indicators, were not available across DHS. Fifth, we presented mean and prevalence estimates at the country level, and we conducted country-level meta-regressions, so the limitations of an ecological analysis apply to these findings. Sixth, we analyzed two or more DHS and the willingness to participate of PLWH may have changed over time due to stigma or overall health status. It is possible that PLWH in the earliest years were less likely to partake in health surveys due to stigma related to their HIV status. Finally, the pooled DHS dataset included relatively young people limiting the extrapolation of our findings to people in their fifth and sixth (or older) decade of life, when people seem to gain weight; for example, women gain ~3 pounds per year independent of initial weight and race [33]. Our analysis still did not capture the BMI distribution across the lifespan. With increasing lifespan across the region and HIV becoming more of a chronic illness, future analyses should include a significant sample of midlife and older adults.

## Conclusions

We provide evidence that mean BMI and prevalence of overweight were similar in PLWH and PLWoH between 2003 and 2019, yet in some cases the estimates for PWLH were on track to catch up with those for PWLoH. ART coverage was not strongly associated with BMI changes nor with closing or widening gaps in BMI levels between PLWH and PLWoH at the population level. Monitoring programs of BMI levels are warranted in PLWH to prevent the rising BMI trends, and to ultimately avoid the negative cardiometabolic consequences of long-term exposure to high BMI. Many more national health surveys collecting HIV information alongside anthropometric data and other markers of noncommunicable diseases, such as blood pressure and glucose and lipid biomarkers, are urgently warranted worldwide.

## Supporting information

S1 Text Table A in S1 Text. List of data sources included in the analysis. Table B in S1 Text. Number of included and excluded observations by survey. Table C in S1 Text. Overall differences between included and excluded observations. Table D in S1 Text. Age and socioeconomic status (SES) standardization. Table E in S1 Text. Distribution of DHS in earliest (blue font) or latest (red font) periods. Table F in S1 Text. Unweighted description of the study sample. Table G in S1 Text. Socioeconomic status by country and HIV status. Table H in S1 Text. Mean body mass index in the earliest and latest surveys by sex and HIV status; not age-SES standardized (i.e., crude mean estimates). Table I in S1 Text. Mean body mass index in the earliest and latest surveys by sex and HIV status; age-SES standardized. Table J in S1 Text. Prevalence (%) of overweight in the earliest and latest surveys by sex and HIV status; not age-SES standardized (i.e., crude prevalence estimates). Table K in S1 Text. Prevalence (%) of overweight in the earliest and latest surveys by sex and HIV status; age-SES standardized. Table L in S1 Text. Mean body mass index stratified by HIV status throughout the observation period by sex and country, not age-SES standardized (i.e., crude mean estimates). Table M in S1 Text. Age-SES standardized mean body mass index stratified by HIV status throughout the observation period by sex and country. Table N in S1 Text. Prevalence of overweight stratified by HIV status throughout the observation period by sex, not age-SES standardized (i.e., crude prevalence estimates). Table O in S1 Text. Age-SES standardized prevalence of overweight stratified by HIV status throughout the observation period by sex. Table P in S1 Text. Study-level meta-regression of mean body mass index, and proportion of overweight, on antiretroviral therapy coverage stratified by sex. Table Q in S1 Text. Mean body mass index throughout the observation period by socioeconomic quintile and HIV status; not age-SES standardized. Table R in S1 Text. Prevalence of overweight throughout the observation period by socioeconomic quintile and HIV status; not age-SES standardized. Table S in S1 Text. Multilevel association between HIV status (independent variable) and body mass index as well as overweight (dependent variable), adjusted by socioeconomic status, age, and sex, overall and stratified by study period (early vs late surveys). Table T in S1 Text. Multilevel association between HIV status (independent variable) and body mass index as well as overweight (dependent variable), adjusted by age and sex, stratified by study period (early vs late surveys), overall and for each level of socioeconomic status. Fig A in S1 Text. Age-SES standardized prevalence of overweight (%) by period, sex, and HIV status. Fig B in S1 Text. Age-SES standardized prevalence (%) of overweight stratified by sex and survey. Fig C in S1 Text. Meta-regression of prevalence (%) of overweight, as well as difference in prevalence of overweight between people without and with HIV, on antiretroviral treatment coverage. Fig D in S1 Text. Mean body mass index throughout the observation period by socioeconomic quintile and HIV status in men; not age-SES standardized. Fig E in S1 Text. Mean body mass index throughout the observation period by socioeconomic quintile and HIV status in women; not age-SES standardized. Fig F in S1 Text. Prevalence of overweight throughout the observation period by socioeconomic quintile and HIV status in men; not age-SES standardized. Fig G in S1 Text. Prevalence of overweight throughout the observation period by socioeconomic quintile and HIV status in women; not age-SES standardized. Fig H in S1 Text. Comparison of crude and age-standardized mean body mass index by region and sex.
(DOCX)
