# Supplementary material for: Trends in body mass index for people with and without HIV: Pooled analysis of nationally-representative health surveys from 10 countries and 173,800 adults in Africa
Source: PLOS Glob Public Health. 2024 Sep 17;4(9):e0003640. doi: 10.1371/journal.pgph.0003640 (PMC11407641; doi:10.1371/journal.pgph.0003640)
Supplement: S1 Text — List of data sources included in the analysis. Table B in S1 Text. Number of included and excluded observations by survey. Table C in S1 Text. Overall differences between included and excluded observations. Table D in S1 Text. Age and socioeconomic status (SES) standardization. Table E in S1 Text. Distribution of DHS in earliest (blue font) or latest (red font) periods. Table F in S1 Text. Unweighted description of the study sample. Table G in S1 Text. Socioeconomic status by country and HIV status. Table H in S1 Text. Mean body mass index in the earliest and latest surveys by sex and HIV status; not age-SES standardized (i.e., crude mean estimates). Table I in S1 Text. Mean body mass index in the earliest and latest surveys by sex and HIV status; age-SES standardized. Table J in S1 Text. Prevalence (%) of overweight in the earliest and latest surveys by sex and HIV status; not age-SES standardized (i.e., crude prevalence estimates). Table K in S1 Text. Prevalence (%) of overweight in the earliest and latest surveys by sex and HIV status; age-SES standardized. Table L in S1 Text. Mean body mass index stratified by HIV status throughout the observation period by sex and country, not age-SES standardized (i.e., crude mean estimates). Table M in S1 Text. Age-SES standardized mean body mass index stratified by HIV status throughout the observation period by sex and country. Table N in S1 Text. Prevalence of overweight stratified by HIV status throughout the observation period by sex, not age-SES standardized (i.e., crude prevalence estimates). Table O in S1 Text. Age-SES standardized prevalence of overweight stratified by HIV status throughout the observation period by sex. Table P in S1 Text. Study-level meta-regression of mean body mass index, and proportion of overweight, on antiretroviral therapy coverage stratified by sex. Table Q in S1 Text. Mean body mass index throughout the observation period by socioeconomic quintile and HIV status; not age-SES standardized. T [file pgph.0003640.s001.docx]

**S1 text:**

**Trends in body mass index for people with and without HIV: Pooled analysis of nationally-representative health surveys from 10 countries and 173,800 adults in Africa**

**Contents**

[Table 1 in S1 text. List of data sources included in the analysis. 4](#_Toc175817758)

[Table 2 in S1 text. Number of included and excluded observations by survey. 6](#_Toc175817759)

[Table 3 in S1 text. Overall differences between included and excluded observations. 7](#_Toc175817760)

[Table 4 in S1 text. Age and socioeconomic status (SES) standardization. 8](#_Toc175817761)

[Table 5 in S1 text. Distribution of DHS in earliest (blue font) or latest (red font) periods. 22](#_Toc175817762)

[Table 6 in S1 text. Unweighted description of the study sample. 23](#_Toc175817763)

[Table 7 in S1 text. Socioeconomic status by country and HIV status. 27](#_Toc175817764)

[Table 8 in S1 text. Mean body mass index in the earliest and latest surveys by sex and HIV status; not age-SES standardized (i.e., crude mean estimates). 30](#_Toc175817765)

[Table 9 in S1 text. Mean body mass index in the earliest and latest surveys by sex and HIV status; age-SES standardized. 31](#_Toc175817766)

[Table 10 in S1 text. Prevalence (%) of overweight in the earliest and latest surveys by sex and HIV status; not age-SES standardized (i.e., crude prevalence estimates). 32](#_Toc175817767)

[Table 11 in S1 text. Prevalence (%) of overweight in the earliest and latest surveys by sex and HIV status; age-SES standardized. 33](#_Toc175817768)

[Table 12 in S1 text. Mean body mass index stratified by HIV status throughout the observation period by sex and country, not age-SES standardized (i.e., crude mean estimates). 34](#_Toc175817769)

[Table 13 in S1 text. Age-SES standardized mean body mass index stratified by HIV status throughout the observation period by sex and country. 36](#_Toc175817770)

[Table 14 in S1 text. Prevalence of overweight stratified by HIV status throughout the observation period by sex, not age-SES standardized (i.e., crude prevalence estimates). 38](#_Toc175817771)

[Table 15 in S1 text. Age-SES standardized prevalence of overweight stratified by HIV status throughout the observation period by sex. 42](#_Toc175817772)

[Table 16 in S1 text. Study-level meta-regression of mean body mass index, and proportion of overweight, on antiretroviral therapy coverage stratified by sex. 46](#_Toc175817773)

[Table 17 in S1 text. Mean body mass index throughout the observation period by socioeconomic quintile and HIV status; not age-SES standardized. 47](#_Toc175817774)

[Table 18 in S1 text. Prevalence of overweight throughout the observation period by socioeconomic quintile and HIV status; not age-SES standardized. 55](#_Toc175817775)

[Table 19 in S1 text. Multilevel association between HIV status (independent variable) and body mass index as well as overweight (dependent variable), adjusted by socioeconomic status, age, and sex, overall and stratified by study period (early vs late surveys). 70](#_Toc175817776)

[Table 20 in S1 text. Multilevel association between HIV status (independent variable) and body mass index as well as overweight (dependent variable), adjusted by age and sex, stratified by study period (early vs late surveys), overall and for each level of socioeconomic status. 71](#_Toc175817777)

[Fig 1 in S1 text. Age-SES standardized prevalence of overweight (%) by period, sex, and HIV status. 72](#_Toc175817778)

[Fig 2 in S1 text. Age-SES standardized prevalence (%) of overweight stratified by sex and survey. 73](#_Toc175817779)

[Fig 3 in S1 text. Meta-regression of prevalence (%) of overweight, as well as difference in prevalence of overweight between people without and with HIV, on antiretroviral treatment coverage. 74](#_Toc175817780)

[Fig 4 in S1 text. Mean body mass index throughout the observation period by socioeconomic quintile and HIV status in men; not age-SES standardized. 75](#_Toc175817781)

[Fig 5 in S1 text. Mean body mass index throughout the observation period by socioeconomic quintile and HIV status in women; not age-SES standardized. 76](#_Toc175817782)

[Fig 6 in S1 text. Prevalence of overweight throughout the observation period by socioeconomic quintile and HIV status in men; not age-SES standardized. 77](#_Toc175817783)

[Fig 7 in S1 text. Prevalence of overweight throughout the observation period by socioeconomic quintile and HIV status in women; not age-SES standardized. 78](#_Toc175817784)

[Fig 8 in S1 text. Comparison of crude and age-standardized mean body mass index by region and sex. 79](#_Toc175817785)

[References 80](#_Toc175817786)

# **Table A in S1 text. List of data sources included in the analysis.**

| **Sex** | **Survey year** | **Country** | **Region** | **Income group** | **Link to data and documentation** | **HIV tests*** |
| --- | --- | --- | --- | --- | --- | --- |
| Women | 2010 | Burundi | East Africa | Low | <https://dhsprogram.com/methodology/survey/survey-display-346.cfm> | Vironostika® HIV Uni-Form Ag/Ab; Enzygnost®HIVIntegral II; inno-lia hiv i/ii score |
| Women | 2016 | Burundi | East Africa | Low | <https://dhsprogram.com/methodology/survey/survey-display-463.cfm> | Bioelisa HIV 1+2 Ag/Ab; Enzygnost®HIVIntegral II; inno-lia hiv i/ii line immunoassay |
| Women | 2005 | Ethiopia | East Africa | Low | <https://dhsprogram.com/methodology/survey/survey-display-248.cfm> | Vironostika HIV Uni-Form Plus O; Enzygnost Anti HIV-1/2 Plus; Genetic Systems HIV-1 Western Blot |
| Men | 2011 | Ethiopia | East Africa | Low | <https://dhsprogram.com/methodology/survey/survey-display-359.cfm> | Vironostika® HIV Uni-Form II Plus O; Murex HIV Ag/Ab Combination; Murex HIV Ag/Ab Combination |
| Women | 2011 | Ethiopia | East Africa | Low |  |  |
| Men | 2016 | Ethiopia | East Africa | Low | <https://dhsprogram.com/methodology/survey/survey-display-478.cfm> | Genscreen ULTRA Ag/Ab; Bioelisa HIV 1+2 Ag/Ab combination; Inno-Lia HIV I/II line immunoassay |
| Women | 2016 | Ethiopia | East Africa | Low |  |  |
| Women | 2005 | Guinea | West Africa | Low | <https://dhsprogram.com/methodology/survey/survey-display-249.cfm> | Vironostika HIV Uniform II Plus O; Enzygnost Anti-HIV1/2 Plus; Western Blot HIV |
| Women | 2012 | Guinea | West Africa | Low | <https://dhsprogram.com/methodology/survey/survey-display-391.cfm> | Vironostika Ab/Ag; Enzygnost Ab/Ag |
| Women | 2018 | Guinea | West Africa | Low | <https://dhsprogram.com/methodology/survey/survey-display-539.cfm> | Bioelisa HIV-1 + 2 Ag / Ab; Genscreen HIV ½; INNO-LIA™ HIV I / II Score |
| Women | 2003 | Kenya | East Africa | Lower-Middle | <https://dhsprogram.com/methodology/survey/survey-display-216.cfm> | Enzygnost Anti-HIV-1/2 Plus; Vironostika HIV-1 MicroELISA; INN-OLIA HIV confirmation Western blot |
| Women | 2008 | Kenya | East Africa | Lower-Middle | <https://dhsprogram.com/methodology/survey/survey-display-300.cfm> | Vironostika Anti-HIV-1/2 Plus; Murex HIV-1/2 MicroELISA System; polymerase chain reaction (PCR) DNA |
| Women | 2004 | Lesotho | Southern Africa | Lower-Middle | <https://dhsprogram.com/methodology/survey/survey-display-256.cfm> | Vironostika HIV Uniform II Plus O; Genscreen HIV1/2 & Enzygnost; Abbott Determine |
| Men | 2009 | Lesotho | Southern Africa | Lower-Middle | <https://dhsprogram.com/methodology/survey/survey-display-317.cfm> |  |
| Women | 2009 | Lesotho | Southern Africa | Lower-Middle |  |  |
| Men | 2014 | Lesotho | Southern Africa | Lower-Middle | <https://dhsprogram.com/methodology/survey/survey-display-462.cfm> | Vironostika® HIV Ag/Ab; Enzygnost® HIV Integral 4 assay; Genetic Systems HIV-1 Western blot |
| Women | 2014 | Lesotho | Southern Africa | Lower-Middle |  |  |
| Women | 2006 | Mali | West Africa | Low | <https://dhsprogram.com/methodology/survey/survey-display-276.cfm> | Vironostika Uniform II; Murex HIV 1.2.0; ImmunoComb®Bispot HIV 1&2; Western Blot |
| Women | 2012 | Mali | West Africa | Low | <https://dhsprogram.com/methodology/survey/survey-display-405.cfm> | Vironostika VIH Uniform II Plus O; Enzignost® HIV Integral II; HIV 2.2 Blot® |
| Women | 2004 | Malawi | East Africa | Low | <https://dhsprogram.com/methodology/survey/survey-display-251.cfm> |  |
| Women | 2010 | Malawi | East Africa | Low | <https://dhsprogram.com/methodology/survey/survey-display-333.cfm> | Vironostika® HIV Uni-Form II Plus O; Enzygnost® Anti-HIV 1/2 Plus; Western Blot 2.2 |
| Women | 2015 | Malawi | East Africa | Low | <https://dhsprogram.com/methodology/survey/survey-display-483.cfm> | Enzygnost Integral II; Murex HIV Ag/Ab combination; InnoLia HIV I/II Score |
| Women | 2006 | Niger | West Africa | Low | <https://dhsprogram.com/methodology/survey/survey-display-277.cfm> | Genscreen® Plus HIV Ag-Ab; Vironostika® HIV Uni-Form II Ag-Ab plus O; ImmunoComb®Bispot HIV 1&2 |
| Women | 2012 | Niger | West Africa | Low | <https://dhsprogram.com/methodology/survey/survey-display-407.cfm> | Vironostika® HIV Uni-Form II plus O; Enzygnost® Anti-HIV1/2 plus; InnoLia |
| Women | 2008 | Sierra Leone | West Africa | Low | <https://dhsprogram.com/methodology/survey/survey-display-324.cfm> | Vironostika HIV Uniform II Plus O; Murex HIV 1.2.O test kit; Western Blot 2.2 |
| Men | 2013 | Sierra Leone | West Africa | Low | <https://dhsprogram.com/methodology/survey/survey-display-450.cfm> | Vironostika Ag/Ab; Enzygnost Integral II (EIA2); Inno-Lia HIV I/II line immunoassay |
| Women | 2013 | Sierra Leone | West Africa | Low |  |  |
| Men | 2019 | Sierra Leone | West Africa | Low | <https://dhsprogram.com/methodology/survey/survey-display-545.cfm> | Determine (Abbott Laboratories) and STAT-PAK (Chembio Diagnostics). |
| Women | 2019 | Sierra Leone | West Africa | Low |  |  |
| Women | 2005 | Zimbabwe | Southern Africa | Lower-Middle | <https://dhsprogram.com/methodology/survey/survey-display-260.cfm> | Vironostika® HIV Uni-Form II Plus O; AniLab Systems; Genetic Systems New LAV Blot I |
| Men | 2010 | Zimbabwe | Southern Africa | Lower-Middle | <https://dhsprogram.com/methodology/survey/survey-display-367.cfm> | Ani Labsystems HIV EIA; Vironostika® HIV Uni-Form II Plus O; HIV 2.2 western blot |
| Women | 2010 | Zimbabwe | Southern Africa | Lower-Middle |  |  |
| Men | 2015 | Zimbabwe | Southern Africa | Lower-Middle | <https://dhsprogram.com/methodology/survey/survey-display-475.cfm> | Vironostika® HIV Ag/Ab; Enzygnost® HIV Integral II; INNO-LIA™ HIV I/II Score Blot Assay; Geenius HIV 1/2 |
| Women | 2015 | Zimbabwe | Southern Africa | Lower-Middle |  |  |

<https://dhsprogram.com/>

*Further details about the methodology for HIV screening are available in the reports (links).

# **Table B in S1 text. Number of included and excluded observations by survey.**

| **Sex** | **Country** | **Year** | **Missing** | **Complete** |
| --- | --- | --- | --- | --- |
| Men | Ethiopia | 2011 | 2765 | 11345 |
| Men | Ethiopia | 2016 | 2763 | 9925 |
| Men | Lesotho | 2009 | 742 | 2575 |
| Men | Lesotho | 2014 | 579 | 2352 |
| Men | Sierra Leone | 2013 | 1472 | 5790 |
| Men | Sierra Leone | 2019 | 2020 | 5177 |
| Men | Zimbabwe | 2010 | 2363 | 5117 |
| Men | Zimbabwe | 2015 | 2197 | 6199 |
| Women | Burundi | 2010 | 6045 | 3344 |
| Women | Burundi | 2016 | 10687 | 6582 |
| Women | Ethiopia | 2005 | 9439 | 4631 |
| Women | Ethiopia | 2011 | 4424 | 12091 |
| Women | Ethiopia | 2016 | 4166 | 11517 |
| Women | Guinea | 2005 | 4986 | 2968 |
| Women | Guinea | 2012 | 5556 | 3586 |
| Women | Guinea | 2018 | 6841 | 4033 |
| Women | Kenya | 2003 | 5644 | 2551 |
| Women | Kenya | 2008 | 5367 | 3077 |
| Women | Lesotho | 2004 | 4743 | 2352 |
| Women | Lesotho | 2009 | 4523 | 3101 |
| Women | Lesotho | 2014 | 3921 | 2700 |
| Women | Mali | 2006 | 11091 | 3492 |
| Women | Mali | 2012 | 6408 | 4016 |
| Women | Malawi | 2004 | 9508 | 2190 |
| Women | Malawi | 2010 | 17423 | 5597 |
| Women | Malawi | 2015 | 18395 | 6167 |
| Women | Niger | 2006 | 5926 | 3297 |
| Women | Niger | 2012 | 7291 | 3869 |
| Women | Sierra Leone | 2008 | 4544 | 2830 |
| Women | Sierra Leone | 2013 | 10543 | 6115 |
| Women | Sierra Leone | 2019 | 9779 | 5795 |
| Women | Zimbabwe | 2005 | 2999 | 5908 |
| Women | Zimbabwe | 2010 | 2931 | 6240 |
| Women | Zimbabwe | 2015 | 2671 | 7284 |

# **Table C in S1 text. Overall differences between included and excluded observations.**

|  | **Included**  **(n=173,813)** | **Excluded**  **(n=283,9982)** | **p-value** |
| --- | --- | --- | --- |
| Age (mean) | 31.1 | 31.0 | 0.279 |
| Sex (%) |  |  |  |
| Men | 27.9 | 34.6 | <0.001 |
| Women | 72.1 | 65.4 |  |
| Wealth Index (%) |  |  |  |
| Poorest | 19.4 | 17.7 | <0.001 |
| Poorer | 17.2 | 17.4 |  |
| Middle | 17.2 | 18.1 |  |
| Richer | 19.6 | 20.0 |  |
| Richest | 26.6 | 26.9 |  |
| Weight (kg) | 57.7 | 54.9 | <0.001 |
| Height (cm) | 161.2 | 158.8 | <0.001 |
| Body mass index (kg/m^2^) | 22.2 | 21.7 | <0.001 |
| Overweight (BMI = 25+ kg/m^2^; %) |  |  |  |
| <25 kg/m^2^ | 81.7 | 84.5 | <0.001 |
| 25+ kg/m^2^ | 18.3 | 15.6 |  |
| HIV test (%) |  |  |  |
| Negative | 92.0 | 96.3 | <0.001 |
| Positive | 8.0 | 3.8 |  |

# **Table D in S1 text. Age and socioeconomic status (SES) standardization.**

We applied direct standardization through a weighted average. We computed the mean BMI and the prevalence of overweight in 10-year age groups accounting for the complex survey design of each DHS. The age-SES-standardization was applied stratified by sex and HIV status, as well as by country and survey period, accordingly. The same process was applied to the lower and upper 95% confidence intervals.

We developed a standard population using the pooled dataset (i.e., including all DHS pooled in the analysis). By sex, we computed the proportion of observations in each 10-year age group and SES quintile. These proportion were then normalized by sex, so that the weights will add up to 1 in men and in women, respectively. The normalized weights were used to compute the age-SES standardized results (mean and prevalence estimates). The weights, together with the 95% confidence interval and standard errors, as well as the normalized weights, are presented in the table below.

| **Sex** | **Age** | **SES** | **Sample size used** | **Weights** | **Lower 95% confidence interval** | **Upper 95% confidence interval** | **Standard error** | **Weights normalized** |
| --- | --- | --- | --- | --- | --- | --- | --- | --- |
| Men | 18-29 | Middle | 3725 | 0.189040305 | 0.180286052 | 0.198116905 | 0.004548079 | **0.047260076** |
| Men | 18-29 | Poorer | 3507 | 0.178491542 | 0.169714101 | 0.187620362 | 0.004567178 | **0.044622886** |
| Men | 18-29 | Poorest | 3770 | 0.144258072 | 0.134766207 | 0.154299249 | 0.004981132 | **0.036064518** |
| Men | 18-29 | Richer | 4633 | 0.229867466 | 0.218367003 | 0.241786250 | 0.005974040 | **0.057466866** |
| Men | 18-29 | Richest | 6433 | 0.258342615 | 0.243820435 | 0.273417003 | 0.007550600 | **0.064585654** |
| Men | 30-39 | Middle | 2038 | 0.184079989 | 0.174126728 | 0.194468221 | 0.005188305 | **0.046019997** |
| Men | 30-39 | Poorer | 2212 | 0.193440464 | 0.182929893 | 0.204403863 | 0.005477293 | **0.048360116** |
| Men | 30-39 | Poorest | 2817 | 0.182942792 | 0.171892134 | 0.194536986 | 0.005775668 | **0.045735698** |
| Men | 30-39 | Richer | 2416 | 0.206047868 | 0.194133444 | 0.218495252 | 0.006214091 | **0.051511967** |
| Men | 30-39 | Richest | 3367 | 0.233488888 | 0.218451505 | 0.249231294 | 0.007852007 | **0.058372222** |
| Men | 40-49 | Middle | 1597 | 0.203605560 | 0.191708889 | 0.216043164 | 0.006207016 | **0.050901390** |
| Men | 40-49 | Poorer | 1550 | 0.190911576 | 0.179953415 | 0.202372352 | 0.005718243 | **0.047727894** |
| Men | 40-49 | Poorest | 2046 | 0.199924786 | 0.187277549 | 0.213202062 | 0.006612520 | **0.049981197** |
| Men | 40-49 | Richer | 1519 | 0.188982826 | 0.176196044 | 0.202469495 | 0.006701160 | **0.047245707** |
| Men | 40-49 | Richest | 2128 | 0.216575253 | 0.201074366 | 0.232922721 | 0.008124078 | **0.054143813** |
| Men | 50-59 | Middle | 866 | 0.210134041 | 0.194661612 | 0.226490410 | 0.008118824 | **0.052533510** |
| Men | 50-59 | Poorer | 926 | 0.210334490 | 0.195224655 | 0.226284958 | 0.007922808 | **0.052583623** |
| Men | 50-59 | Poorest | 1034 | 0.169479889 | 0.154548645 | 0.185537100 | 0.007902046 | **0.042369972** |
| Men | 50-59 | Richer | 854 | 0.212027496 | 0.195351152 | 0.229721008 | 0.008767086 | **0.053006874** |
| Men | 50-59 | Richest | 1042 | 0.198024084 | 0.178041123 | 0.219650539 | 0.010612295 | **0.049506021** |
| Women | 18-29 | Middle | 10508 | 0.182134303 | 0.177063439 | 0.187317335 | 0.002615503 | **0.060711434** |
| Women | 18-29 | Poorer | 10515 | 0.180140133 | 0.175181313 | 0.185207803 | 0.002557495 | **0.060046711** |
| Women | 18-29 | Poorest | 11221 | 0.167477837 | 0.161503942 | 0.173626945 | 0.003092170 | **0.055825946** |
| Women | 18-29 | Richer | 12612 | 0.207992868 | 0.202007527 | 0.214107968 | 0.003086565 | **0.069330956** |
| Women | 18-29 | Richest | 18690 | 0.262254859 | 0.253654041 | 0.271041402 | 0.004435398 | **0.087418286** |
| Women | 30-39 | Middle | 6506 | 0.192611034 | 0.186765067 | 0.198595301 | 0.003017601 | **0.064203678** |
| Women | 30-39 | Poorer | 6462 | 0.186998079 | 0.181476371 | 0.192648251 | 0.002849661 | **0.062332693** |
| Women | 30-39 | Poorest | 7668 | 0.190391155 | 0.183978144 | 0.196973749 | 0.003314838 | **0.063463718** |
| Women | 30-39 | Richer | 7194 | 0.202928076 | 0.196647876 | 0.209356573 | 0.003241703 | **0.067642692** |
| Women | 30-39 | Richest | 9316 | 0.227071656 | 0.218846583 | 0.235512658 | 0.004251258 | **0.075690552** |
| Women | 40-49 | Middle | 4724 | 0.206791256 | 0.199996605 | 0.213755068 | 0.003509485 | **0.068930419** |
| Women | 40-49 | Poorer | 4672 | 0.202275947 | 0.195633124 | 0.209085698 | 0.003431443 | **0.067425316** |
| Women | 40-49 | Poorest | 5212 | 0.196320195 | 0.188913682 | 0.203944072 | 0.003833866 | **0.065440065** |
| Women | 40-49 | Richer | 4820 | 0.207506645 | 0.200248201 | 0.214957473 | 0.003752016 | **0.069168882** |
| Women | 40-49 | Richest | 5213 | 0.187105957 | 0.179173711 | 0.195305817 | 0.004114800 | **0.062368652** |

The age-SES standardization worked very well in most instances, as shown in the table below where the distribution of each sex-age group-SES quantile was rather similar (~20%). This implies that, through this standardization, we fixed the imbalanced distribution of SES and age between populations.

| **DHS** | **Sex** | **Age** | **SES** | **Weighted proportion** | **Lower 95% confidence interval** | **Upper 95% confidence interval** |
| --- | --- | --- | --- | --- | --- | --- |
| Burundi, 2010 | Women | 18-29 | Middle | 0.195560628 | 0.172000186 | 0.221485079 |
| Burundi, 2010 | Women | 18-29 | Poorer | 0.195217914 | 0.173740590 | 0.218647637 |
| Burundi, 2010 | Women | 18-29 | Poorest | 0.199057526 | 0.174977477 | 0.225545480 |
| Burundi, 2010 | Women | 18-29 | Richer | 0.192434444 | 0.168908383 | 0.218376284 |
| Burundi, 2010 | Women | 18-29 | Richest | 0.217729488 | 0.189523426 | 0.248844481 |
| Burundi, 2010 | Women | 30-39 | Middle | 0.164708743 | 0.138625598 | 0.194590889 |
| Burundi, 2010 | Women | 30-39 | Poorer | 0.204565783 | 0.175467386 | 0.237102057 |
| Burundi, 2010 | Women | 30-39 | Poorest | 0.227485767 | 0.194812958 | 0.263842680 |
| Burundi, 2010 | Women | 30-39 | Richer | 0.200337375 | 0.170598914 | 0.233798489 |
| Burundi, 2010 | Women | 30-39 | Richest | 0.202902333 | 0.171812644 | 0.238001042 |
| Burundi, 2010 | Women | 40-49 | Middle | 0.232990308 | 0.198797712 | 0.271074174 |
| Burundi, 2010 | Women | 40-49 | Poorer | 0.221765485 | 0.188374417 | 0.259185285 |
| Burundi, 2010 | Women | 40-49 | Poorest | 0.196866449 | 0.163425958 | 0.235225590 |
| Burundi, 2010 | Women | 40-49 | Richer | 0.204800211 | 0.172800802 | 0.240998892 |
| Burundi, 2010 | Women | 40-49 | Richest | 0.143577548 | 0.115870706 | 0.176586361 |
| Burundi, 2016 | Women | 18-29 | Middle | 0.207549510 | 0.190524715 | 0.225671479 |
| Burundi, 2016 | Women | 18-29 | Poorer | 0.191129988 | 0.173502248 | 0.210093439 |
| Burundi, 2016 | Women | 18-29 | Poorest | 0.168930970 | 0.153506990 | 0.185564979 |
| Burundi, 2016 | Women | 18-29 | Richer | 0.208458089 | 0.189294078 | 0.229014184 |
| Burundi, 2016 | Women | 18-29 | Richest | 0.223931443 | 0.194383364 | 0.256540803 |
| Burundi, 2016 | Women | 30-39 | Middle | 0.191854290 | 0.170594517 | 0.215076457 |
| Burundi, 2016 | Women | 30-39 | Poorer | 0.184355181 | 0.164889217 | 0.205553557 |
| Burundi, 2016 | Women | 30-39 | Poorest | 0.222915847 | 0.200295489 | 0.247300841 |
| Burundi, 2016 | Women | 30-39 | Richer | 0.182687089 | 0.163279618 | 0.203839368 |
| Burundi, 2016 | Women | 30-39 | Richest | 0.218187592 | 0.189819064 | 0.249490223 |
| Burundi, 2016 | Women | 40-49 | Middle | 0.197518566 | 0.174918673 | 0.222251868 |
| Burundi, 2016 | Women | 40-49 | Poorer | 0.239663820 | 0.215158838 | 0.266013777 |
| Burundi, 2016 | Women | 40-49 | Poorest | 0.213568477 | 0.190510462 | 0.238594696 |
| Burundi, 2016 | Women | 40-49 | Richer | 0.206984048 | 0.183281764 | 0.232877538 |
| Burundi, 2016 | Women | 40-49 | Richest | 0.142265089 | 0.120846793 | 0.166759413 |
| Ethiopia, 2005 | Women | 18-29 | Middle | 0.208194157 | 0.182420485 | 0.236555702 |
| Ethiopia, 2005 | Women | 18-29 | Poorer | 0.178177404 | 0.154751566 | 0.204292293 |
| Ethiopia, 2005 | Women | 18-29 | Poorest | 0.163172972 | 0.138589607 | 0.191149353 |
| Ethiopia, 2005 | Women | 18-29 | Richer | 0.185108436 | 0.162385636 | 0.210212884 |
| Ethiopia, 2005 | Women | 18-29 | Richest | 0.265347031 | 0.224033385 | 0.311223611 |
| Ethiopia, 2005 | Women | 30-39 | Middle | 0.172318290 | 0.143260692 | 0.205853521 |
| Ethiopia, 2005 | Women | 30-39 | Poorer | 0.220388631 | 0.189879392 | 0.254261437 |
| Ethiopia, 2005 | Women | 30-39 | Poorest | 0.200504246 | 0.168949655 | 0.236276698 |
| Ethiopia, 2005 | Women | 30-39 | Richer | 0.193149853 | 0.165162344 | 0.224604025 |
| Ethiopia, 2005 | Women | 30-39 | Richest | 0.213638980 | 0.179167301 | 0.252701161 |
| Ethiopia, 2005 | Women | 40-49 | Middle | 0.173513079 | 0.138511593 | 0.215150423 |
| Ethiopia, 2005 | Women | 40-49 | Poorer | 0.201592772 | 0.167958975 | 0.240018844 |
| Ethiopia, 2005 | Women | 40-49 | Poorest | 0.228615800 | 0.188031429 | 0.274993156 |
| Ethiopia, 2005 | Women | 40-49 | Richer | 0.206611069 | 0.168693541 | 0.250483357 |
| Ethiopia, 2005 | Women | 40-49 | Richest | 0.189667281 | 0.150710804 | 0.235896498 |
| Ethiopia, 2011 | Men | 18-29 | Middle | 0.190256322 | 0.171357899 | 0.210708998 |
| Ethiopia, 2011 | Men | 18-29 | Poorer | 0.184797318 | 0.165434266 | 0.205867645 |
| Ethiopia, 2011 | Men | 18-29 | Poorest | 0.144776194 | 0.122644996 | 0.170126565 |
| Ethiopia, 2011 | Men | 18-29 | Richer | 0.219853693 | 0.191346223 | 0.251288515 |
| Ethiopia, 2011 | Men | 18-29 | Richest | 0.260316475 | 0.231567243 | 0.291281990 |
| Ethiopia, 2011 | Men | 30-39 | Middle | 0.195042308 | 0.173631018 | 0.218396133 |
| Ethiopia, 2011 | Men | 30-39 | Poorer | 0.191160926 | 0.168603448 | 0.215952465 |
| Ethiopia, 2011 | Men | 30-39 | Poorest | 0.171501002 | 0.147889788 | 0.198005890 |
| Ethiopia, 2011 | Men | 30-39 | Richer | 0.202262456 | 0.175307028 | 0.232195626 |
| Ethiopia, 2011 | Men | 30-39 | Richest | 0.240033310 | 0.208373075 | 0.274833929 |
| Ethiopia, 2011 | Men | 40-49 | Middle | 0.200305654 | 0.171513868 | 0.232573883 |
| Ethiopia, 2011 | Men | 40-49 | Poorer | 0.193341058 | 0.168818628 | 0.220480703 |
| Ethiopia, 2011 | Men | 40-49 | Poorest | 0.225220817 | 0.194301872 | 0.259475331 |
| Ethiopia, 2011 | Men | 40-49 | Richer | 0.192233312 | 0.161818347 | 0.226818012 |
| Ethiopia, 2011 | Men | 40-49 | Richest | 0.188899159 | 0.160219466 | 0.221359378 |
| Ethiopia, 2011 | Men | 50-59 | Middle | 0.238569990 | 0.204590712 | 0.276232820 |
| Ethiopia, 2011 | Men | 50-59 | Poorer | 0.209822180 | 0.178508059 | 0.244991256 |
| Ethiopia, 2011 | Men | 50-59 | Poorest | 0.164773150 | 0.131189730 | 0.204925847 |
| Ethiopia, 2011 | Men | 50-59 | Richer | 0.212415640 | 0.178051033 | 0.251384279 |
| Ethiopia, 2011 | Men | 50-59 | Richest | 0.174419041 | 0.137758989 | 0.218364273 |
| Ethiopia, 2011 | Women | 18-29 | Middle | 0.179286158 | 0.161451245 | 0.198624562 |
| Ethiopia, 2011 | Women | 18-29 | Poorer | 0.176687752 | 0.159469152 | 0.195333464 |
| Ethiopia, 2011 | Women | 18-29 | Poorest | 0.161463011 | 0.140621425 | 0.184729552 |
| Ethiopia, 2011 | Women | 18-29 | Richer | 0.186409634 | 0.161422404 | 0.214276407 |
| Ethiopia, 2011 | Women | 18-29 | Richest | 0.296153446 | 0.268002851 | 0.325944287 |
| Ethiopia, 2011 | Women | 30-39 | Middle | 0.190704795 | 0.168723665 | 0.214809601 |
| Ethiopia, 2011 | Women | 30-39 | Poorer | 0.188692614 | 0.167509747 | 0.211872463 |
| Ethiopia, 2011 | Women | 30-39 | Poorest | 0.195467345 | 0.170855614 | 0.222672275 |
| Ethiopia, 2011 | Women | 30-39 | Richer | 0.185828216 | 0.160849621 | 0.213697959 |
| Ethiopia, 2011 | Women | 30-39 | Richest | 0.239307033 | 0.207985895 | 0.273714832 |
| Ethiopia, 2011 | Women | 40-49 | Middle | 0.203857288 | 0.179252486 | 0.230889328 |
| Ethiopia, 2011 | Women | 40-49 | Poorer | 0.211477785 | 0.185110796 | 0.240492073 |
| Ethiopia, 2011 | Women | 40-49 | Poorest | 0.219251314 | 0.187535150 | 0.254650159 |
| Ethiopia, 2011 | Women | 40-49 | Richer | 0.202640032 | 0.174302289 | 0.234277686 |
| Ethiopia, 2011 | Women | 40-49 | Richest | 0.162773583 | 0.138664873 | 0.190148568 |
| Ethiopia, 2016 | Men | 18-29 | Middle | 0.179326758 | 0.156965573 | 0.204102261 |
| Ethiopia, 2016 | Men | 18-29 | Poorer | 0.184167422 | 0.160540640 | 0.210399855 |
| Ethiopia, 2016 | Men | 18-29 | Poorest | 0.154848876 | 0.128558861 | 0.185371516 |
| Ethiopia, 2016 | Men | 18-29 | Richer | 0.228665974 | 0.200532232 | 0.259465746 |
| Ethiopia, 2016 | Men | 18-29 | Richest | 0.252990972 | 0.213668731 | 0.296818209 |
| Ethiopia, 2016 | Men | 30-39 | Middle | 0.208238095 | 0.182667215 | 0.236353417 |
| Ethiopia, 2016 | Men | 30-39 | Poorer | 0.200176597 | 0.171245053 | 0.232624074 |
| Ethiopia, 2016 | Men | 30-39 | Poorest | 0.158270774 | 0.132959151 | 0.187359749 |
| Ethiopia, 2016 | Men | 30-39 | Richer | 0.205508606 | 0.176020138 | 0.238507274 |
| Ethiopia, 2016 | Men | 30-39 | Richest | 0.227805932 | 0.187859835 | 0.273386768 |
| Ethiopia, 2016 | Men | 40-49 | Middle | 0.216965534 | 0.189416275 | 0.247299199 |
| Ethiopia, 2016 | Men | 40-49 | Poorer | 0.197702756 | 0.171269943 | 0.227097153 |
| Ethiopia, 2016 | Men | 40-49 | Poorest | 0.178663465 | 0.149408656 | 0.212217329 |
| Ethiopia, 2016 | Men | 40-49 | Richer | 0.193555553 | 0.163966412 | 0.227034276 |
| Ethiopia, 2016 | Men | 40-49 | Richest | 0.213112694 | 0.173709438 | 0.258656069 |
| Ethiopia, 2016 | Men | 50-59 | Middle | 0.188997722 | 0.154332117 | 0.229338178 |
| Ethiopia, 2016 | Men | 50-59 | Poorer | 0.187854607 | 0.152410836 | 0.229310981 |
| Ethiopia, 2016 | Men | 50-59 | Poorest | 0.154212167 | 0.120108392 | 0.195844100 |
| Ethiopia, 2016 | Men | 50-59 | Richer | 0.249851225 | 0.207192718 | 0.297991400 |
| Ethiopia, 2016 | Men | 50-59 | Richest | 0.219084280 | 0.164930130 | 0.284952190 |
| Ethiopia, 2016 | Women | 18-29 | Middle | 0.187206826 | 0.165515860 | 0.211021577 |
| Ethiopia, 2016 | Women | 18-29 | Poorer | 0.175936696 | 0.152939419 | 0.201569148 |
| Ethiopia, 2016 | Women | 18-29 | Poorest | 0.159689653 | 0.133434288 | 0.189978601 |
| Ethiopia, 2016 | Women | 18-29 | Richer | 0.184322422 | 0.160481520 | 0.210815702 |
| Ethiopia, 2016 | Women | 18-29 | Richest | 0.292844406 | 0.247394949 | 0.342839914 |
| Ethiopia, 2016 | Women | 30-39 | Middle | 0.210507556 | 0.186350955 | 0.236883895 |
| Ethiopia, 2016 | Women | 30-39 | Poorer | 0.175819436 | 0.153684651 | 0.200387389 |
| Ethiopia, 2016 | Women | 30-39 | Poorest | 0.182558290 | 0.154674821 | 0.214194681 |
| Ethiopia, 2016 | Women | 30-39 | Richer | 0.193864492 | 0.168883520 | 0.221555572 |
| Ethiopia, 2016 | Women | 30-39 | Richest | 0.237250229 | 0.199295448 | 0.279906469 |
| Ethiopia, 2016 | Women | 40-49 | Middle | 0.186518390 | 0.161205334 | 0.214788390 |
| Ethiopia, 2016 | Women | 40-49 | Poorer | 0.186467435 | 0.159858270 | 0.216365147 |
| Ethiopia, 2016 | Women | 40-49 | Poorest | 0.172176677 | 0.142435420 | 0.206631723 |
| Ethiopia, 2016 | Women | 40-49 | Richer | 0.243307442 | 0.211299573 | 0.278452093 |
| Ethiopia, 2016 | Women | 40-49 | Richest | 0.211530059 | 0.173908966 | 0.254779563 |
| Guinea, 2005 | Women | 18-29 | Middle | 0.172710554 | 0.142853918 | 0.207298119 |
| Guinea, 2005 | Women | 18-29 | Poorer | 0.176612360 | 0.147829236 | 0.209621153 |
| Guinea, 2005 | Women | 18-29 | Poorest | 0.176025633 | 0.140165046 | 0.218727086 |
| Guinea, 2005 | Women | 18-29 | Richer | 0.217368868 | 0.181258525 | 0.258402652 |
| Guinea, 2005 | Women | 18-29 | Richest | 0.257282584 | 0.204629287 | 0.318066233 |
| Guinea, 2005 | Women | 30-39 | Middle | 0.208716088 | 0.175721921 | 0.246056051 |
| Guinea, 2005 | Women | 30-39 | Poorer | 0.175478016 | 0.145145880 | 0.210587369 |
| Guinea, 2005 | Women | 30-39 | Poorest | 0.255316600 | 0.208356953 | 0.308732477 |
| Guinea, 2005 | Women | 30-39 | Richer | 0.188833872 | 0.154938531 | 0.228142492 |
| Guinea, 2005 | Women | 30-39 | Richest | 0.171655423 | 0.129950929 | 0.223308759 |
| Guinea, 2005 | Women | 40-49 | Middle | 0.232741135 | 0.191554016 | 0.279720022 |
| Guinea, 2005 | Women | 40-49 | Poorer | 0.210414037 | 0.174363727 | 0.251646110 |
| Guinea, 2005 | Women | 40-49 | Poorest | 0.223968633 | 0.180165924 | 0.274850584 |
| Guinea, 2005 | Women | 40-49 | Richer | 0.191498225 | 0.155000582 | 0.234207889 |
| Guinea, 2005 | Women | 40-49 | Richest | 0.141377973 | 0.105876879 | 0.186302480 |
| Guinea, 2012 | Women | 18-29 | Middle | 0.164661048 | 0.139043880 | 0.193934745 |
| Guinea, 2012 | Women | 18-29 | Poorer | 0.201840806 | 0.169145947 | 0.239037193 |
| Guinea, 2012 | Women | 18-29 | Poorest | 0.167238653 | 0.137740538 | 0.201577171 |
| Guinea, 2012 | Women | 18-29 | Richer | 0.203486022 | 0.172051781 | 0.239005494 |
| Guinea, 2012 | Women | 18-29 | Richest | 0.262773470 | 0.212106001 | 0.320619051 |
| Guinea, 2012 | Women | 30-39 | Middle | 0.219450676 | 0.184536577 | 0.258873461 |
| Guinea, 2012 | Women | 30-39 | Poorer | 0.189078629 | 0.155296751 | 0.228223654 |
| Guinea, 2012 | Women | 30-39 | Poorest | 0.177473452 | 0.146103584 | 0.213891591 |
| Guinea, 2012 | Women | 30-39 | Richer | 0.192283950 | 0.159001295 | 0.230622956 |
| Guinea, 2012 | Women | 30-39 | Richest | 0.221713293 | 0.171327210 | 0.281877081 |
| Guinea, 2012 | Women | 40-49 | Middle | 0.239327284 | 0.202111935 | 0.280982009 |
| Guinea, 2012 | Women | 40-49 | Poorer | 0.211599439 | 0.176069387 | 0.252105491 |
| Guinea, 2012 | Women | 40-49 | Poorest | 0.195913629 | 0.152736465 | 0.247727775 |
| Guinea, 2012 | Women | 40-49 | Richer | 0.204019225 | 0.169329945 | 0.243729873 |
| Guinea, 2012 | Women | 40-49 | Richest | 0.149140424 | 0.110202399 | 0.198763221 |
| Guinea, 2018 | Women | 18-29 | Middle | 0.167366173 | 0.144416323 | 0.193139798 |
| Guinea, 2018 | Women | 18-29 | Poorer | 0.186215794 | 0.161532486 | 0.213709529 |
| Guinea, 2018 | Women | 18-29 | Poorest | 0.179284097 | 0.151568362 | 0.210808659 |
| Guinea, 2018 | Women | 18-29 | Richer | 0.190104195 | 0.166986204 | 0.215594366 |
| Guinea, 2018 | Women | 18-29 | Richest | 0.277029742 | 0.241440521 | 0.315681778 |
| Guinea, 2018 | Women | 30-39 | Middle | 0.204664842 | 0.176000095 | 0.236657315 |
| Guinea, 2018 | Women | 30-39 | Poorer | 0.221050581 | 0.193496506 | 0.251305752 |
| Guinea, 2018 | Women | 30-39 | Poorest | 0.204229970 | 0.174855525 | 0.237121027 |
| Guinea, 2018 | Women | 30-39 | Richer | 0.175442307 | 0.151081103 | 0.202793283 |
| Guinea, 2018 | Women | 30-39 | Richest | 0.194612301 | 0.161297248 | 0.232897611 |
| Guinea, 2018 | Women | 40-49 | Middle | 0.218887799 | 0.187907915 | 0.253381637 |
| Guinea, 2018 | Women | 40-49 | Poorer | 0.245319700 | 0.213243291 | 0.280500852 |
| Guinea, 2018 | Women | 40-49 | Poorest | 0.224042591 | 0.191205772 | 0.260700943 |
| Guinea, 2018 | Women | 40-49 | Richer | 0.165242391 | 0.136474807 | 0.198678726 |
| Guinea, 2018 | Women | 40-49 | Richest | 0.146507519 | 0.117153074 | 0.181703346 |
| Kenya, 2003 | Women | 18-29 | Middle | 0.193278131 | 0.164209559 | 0.226100413 |
| Kenya, 2003 | Women | 18-29 | Poorer | 0.191275427 | 0.160217777 | 0.226728075 |
| Kenya, 2003 | Women | 18-29 | Poorest | 0.135114988 | 0.109575279 | 0.165501043 |
| Kenya, 2003 | Women | 18-29 | Richer | 0.202051696 | 0.172167771 | 0.235646179 |
| Kenya, 2003 | Women | 18-29 | Richest | 0.278279758 | 0.228025693 | 0.334805803 |
| Kenya, 2003 | Women | 30-39 | Middle | 0.173580147 | 0.142815831 | 0.209352944 |
| Kenya, 2003 | Women | 30-39 | Poorer | 0.200326149 | 0.165575179 | 0.240270504 |
| Kenya, 2003 | Women | 30-39 | Poorest | 0.173391611 | 0.137610756 | 0.216144234 |
| Kenya, 2003 | Women | 30-39 | Richer | 0.213650936 | 0.178237439 | 0.253926437 |
| Kenya, 2003 | Women | 30-39 | Richest | 0.239051157 | 0.195705246 | 0.288553240 |
| Kenya, 2003 | Women | 40-49 | Middle | 0.203338707 | 0.163877151 | 0.249467455 |
| Kenya, 2003 | Women | 40-49 | Poorer | 0.206943515 | 0.167249952 | 0.253193326 |
| Kenya, 2003 | Women | 40-49 | Poorest | 0.186392673 | 0.142735101 | 0.239670284 |
| Kenya, 2003 | Women | 40-49 | Richer | 0.241247220 | 0.196528577 | 0.292437745 |
| Kenya, 2003 | Women | 40-49 | Richest | 0.162077888 | 0.119484406 | 0.216128069 |
| Kenya, 2008 | Women | 18-29 | Middle | 0.154673754 | 0.128680398 | 0.184804107 |
| Kenya, 2008 | Women | 18-29 | Poorer | 0.159710838 | 0.131150030 | 0.193109001 |
| Kenya, 2008 | Women | 18-29 | Poorest | 0.169429336 | 0.132728389 | 0.213777046 |
| Kenya, 2008 | Women | 18-29 | Richer | 0.226430821 | 0.189663485 | 0.267968714 |
| Kenya, 2008 | Women | 18-29 | Richest | 0.289755252 | 0.243136367 | 0.341282224 |
| Kenya, 2008 | Women | 30-39 | Middle | 0.165927976 | 0.133057241 | 0.204998986 |
| Kenya, 2008 | Women | 30-39 | Poorer | 0.176896926 | 0.144371952 | 0.214908885 |
| Kenya, 2008 | Women | 30-39 | Poorest | 0.155140422 | 0.120614532 | 0.197331619 |
| Kenya, 2008 | Women | 30-39 | Richer | 0.231992381 | 0.182270217 | 0.290460495 |
| Kenya, 2008 | Women | 30-39 | Richest | 0.270042295 | 0.210265460 | 0.339507448 |
| Kenya, 2008 | Women | 40-49 | Middle | 0.208191552 | 0.170308981 | 0.251941774 |
| Kenya, 2008 | Women | 40-49 | Poorer | 0.179097511 | 0.145441940 | 0.218549313 |
| Kenya, 2008 | Women | 40-49 | Poorest | 0.201152332 | 0.152042198 | 0.261238222 |
| Kenya, 2008 | Women | 40-49 | Richer | 0.213425101 | 0.168871361 | 0.265971900 |
| Kenya, 2008 | Women | 40-49 | Richest | 0.198133505 | 0.147192814 | 0.261302062 |
| Lesotho, 2004 | Women | 18-29 | Middle | 0.194712524 | 0.162928716 | 0.230985707 |
| Lesotho, 2004 | Women | 18-29 | Poorer | 0.192455455 | 0.164813905 | 0.223492332 |
| Lesotho, 2004 | Women | 18-29 | Poorest | 0.147682319 | 0.121108438 | 0.178900208 |
| Lesotho, 2004 | Women | 18-29 | Richer | 0.224404392 | 0.192412279 | 0.260003243 |
| Lesotho, 2004 | Women | 18-29 | Richest | 0.240745309 | 0.202606373 | 0.283510987 |
| Lesotho, 2004 | Women | 30-39 | Middle | 0.181879650 | 0.147737676 | 0.221857848 |
| Lesotho, 2004 | Women | 30-39 | Poorer | 0.180832832 | 0.148572352 | 0.218302208 |
| Lesotho, 2004 | Women | 30-39 | Poorest | 0.142967802 | 0.111643390 | 0.181287531 |
| Lesotho, 2004 | Women | 30-39 | Richer | 0.202644533 | 0.167718478 | 0.242722578 |
| Lesotho, 2004 | Women | 30-39 | Richest | 0.291675183 | 0.241102235 | 0.347991867 |
| Lesotho, 2004 | Women | 40-49 | Middle | 0.174646245 | 0.137201347 | 0.219708240 |
| Lesotho, 2004 | Women | 40-49 | Poorer | 0.198195575 | 0.161244625 | 0.241179392 |
| Lesotho, 2004 | Women | 40-49 | Poorest | 0.135922934 | 0.105153424 | 0.173945893 |
| Lesotho, 2004 | Women | 40-49 | Richer | 0.261718643 | 0.218460618 | 0.310143158 |
| Lesotho, 2004 | Women | 40-49 | Richest | 0.229516603 | 0.184386874 | 0.281874692 |
| Lesotho, 2009 | Men | 18-29 | Middle | 0.227695462 | 0.198354663 | 0.259968903 |
| Lesotho, 2009 | Men | 18-29 | Poorer | 0.208665163 | 0.177252903 | 0.243993332 |
| Lesotho, 2009 | Men | 18-29 | Poorest | 0.140193520 | 0.114995249 | 0.169853629 |
| Lesotho, 2009 | Men | 18-29 | Richer | 0.217883375 | 0.190102209 | 0.248478842 |
| Lesotho, 2009 | Men | 18-29 | Richest | 0.205562480 | 0.174117459 | 0.241029023 |
| Lesotho, 2009 | Men | 30-39 | Middle | 0.192888481 | 0.159800339 | 0.230944647 |
| Lesotho, 2009 | Men | 30-39 | Poorer | 0.182880989 | 0.151381716 | 0.219241267 |
| Lesotho, 2009 | Men | 30-39 | Poorest | 0.186927208 | 0.148428328 | 0.232683312 |
| Lesotho, 2009 | Men | 30-39 | Richer | 0.196854857 | 0.156209298 | 0.245005502 |
| Lesotho, 2009 | Men | 30-39 | Richest | 0.240448465 | 0.197251696 | 0.289691233 |
| Lesotho, 2009 | Men | 40-49 | Middle | 0.168987133 | 0.130236610 | 0.216398824 |
| Lesotho, 2009 | Men | 40-49 | Poorer | 0.201114317 | 0.156643822 | 0.254401421 |
| Lesotho, 2009 | Men | 40-49 | Poorest | 0.194504319 | 0.152791997 | 0.244320177 |
| Lesotho, 2009 | Men | 40-49 | Richer | 0.188256631 | 0.138576304 | 0.250566933 |
| Lesotho, 2009 | Men | 40-49 | Richest | 0.247137603 | 0.189417908 | 0.315597725 |
| Lesotho, 2009 | Men | 50-59 | Middle | 0.205650439 | 0.159732033 | 0.260674007 |
| Lesotho, 2009 | Men | 50-59 | Poorer | 0.256320909 | 0.210468471 | 0.308262492 |
| Lesotho, 2009 | Men | 50-59 | Poorest | 0.140299738 | 0.107226613 | 0.181500102 |
| Lesotho, 2009 | Men | 50-59 | Richer | 0.195791551 | 0.139909849 | 0.267062658 |
| Lesotho, 2009 | Men | 50-59 | Richest | 0.201937363 | 0.148611789 | 0.268366072 |
| Lesotho, 2009 | Women | 18-29 | Middle | 0.174150890 | 0.151855926 | 0.198951305 |
| Lesotho, 2009 | Women | 18-29 | Poorer | 0.164955409 | 0.142322059 | 0.190389127 |
| Lesotho, 2009 | Women | 18-29 | Poorest | 0.140220385 | 0.117911336 | 0.165956225 |
| Lesotho, 2009 | Women | 18-29 | Richer | 0.262859293 | 0.231202388 | 0.297175251 |
| Lesotho, 2009 | Women | 18-29 | Richest | 0.257814025 | 0.224272312 | 0.294467920 |
| Lesotho, 2009 | Women | 30-39 | Middle | 0.174929180 | 0.148304038 | 0.205182794 |
| Lesotho, 2009 | Women | 30-39 | Poorer | 0.141543085 | 0.118404048 | 0.168340591 |
| Lesotho, 2009 | Women | 30-39 | Poorest | 0.133383396 | 0.108714584 | 0.162628512 |
| Lesotho, 2009 | Women | 30-39 | Richer | 0.248297042 | 0.212108860 | 0.288399368 |
| Lesotho, 2009 | Women | 30-39 | Richest | 0.301847298 | 0.259498826 | 0.347860229 |
| Lesotho, 2009 | Women | 40-49 | Middle | 0.204283591 | 0.160105465 | 0.256922900 |
| Lesotho, 2009 | Women | 40-49 | Poorer | 0.170396149 | 0.137564811 | 0.209162720 |
| Lesotho, 2009 | Women | 40-49 | Poorest | 0.153300325 | 0.125237788 | 0.186311678 |
| Lesotho, 2009 | Women | 40-49 | Richer | 0.210664640 | 0.175925313 | 0.250181229 |
| Lesotho, 2009 | Women | 40-49 | Richest | 0.261355297 | 0.211317998 | 0.318456671 |
| Lesotho, 2014 | Men | 18-29 | Middle | 0.210568080 | 0.179981292 | 0.244801136 |
| Lesotho, 2014 | Men | 18-29 | Poorer | 0.182657770 | 0.154020451 | 0.215264806 |
| Lesotho, 2014 | Men | 18-29 | Poorest | 0.111857923 | 0.091751988 | 0.135711410 |
| Lesotho, 2014 | Men | 18-29 | Richer | 0.250152115 | 0.214882476 | 0.289079230 |
| Lesotho, 2014 | Men | 18-29 | Richest | 0.244764112 | 0.204297261 | 0.290321976 |
| Lesotho, 2014 | Men | 30-39 | Middle | 0.162300591 | 0.127040247 | 0.205048767 |
| Lesotho, 2014 | Men | 30-39 | Poorer | 0.201927807 | 0.163164585 | 0.247179946 |
| Lesotho, 2014 | Men | 30-39 | Poorest | 0.154297181 | 0.123525087 | 0.191064018 |
| Lesotho, 2014 | Men | 30-39 | Richer | 0.245738918 | 0.198627154 | 0.299843972 |
| Lesotho, 2014 | Men | 30-39 | Richest | 0.235735504 | 0.193138490 | 0.284415713 |
| Lesotho, 2014 | Men | 40-49 | Middle | 0.210677724 | 0.165422975 | 0.264390768 |
| Lesotho, 2014 | Men | 40-49 | Poorer | 0.154313498 | 0.113965939 | 0.205630278 |
| Lesotho, 2014 | Men | 40-49 | Poorest | 0.206622311 | 0.161081994 | 0.261031495 |
| Lesotho, 2014 | Men | 40-49 | Richer | 0.173745446 | 0.128920248 | 0.230040293 |
| Lesotho, 2014 | Men | 40-49 | Richest | 0.254641021 | 0.196002017 | 0.323758913 |
| Lesotho, 2014 | Men | 50-59 | Middle | 0.240639929 | 0.186574040 | 0.304508054 |
| Lesotho, 2014 | Men | 50-59 | Poorer | 0.203713642 | 0.155217271 | 0.262651311 |
| Lesotho, 2014 | Men | 50-59 | Poorest | 0.152636234 | 0.114939417 | 0.199904049 |
| Lesotho, 2014 | Men | 50-59 | Richer | 0.167576928 | 0.121586665 | 0.226477941 |
| Lesotho, 2014 | Men | 50-59 | Richest | 0.235433267 | 0.183750010 | 0.296375179 |
| Lesotho, 2014 | Women | 18-29 | Middle | 0.180226921 | 0.154109822 | 0.209672998 |
| Lesotho, 2014 | Women | 18-29 | Poorer | 0.178705890 | 0.154456545 | 0.205835547 |
| Lesotho, 2014 | Women | 18-29 | Poorest | 0.150465335 | 0.124337620 | 0.180948866 |
| Lesotho, 2014 | Women | 18-29 | Richer | 0.240949802 | 0.210554255 | 0.274209133 |
| Lesotho, 2014 | Women | 18-29 | Richest | 0.249652052 | 0.211336327 | 0.292339509 |
| Lesotho, 2014 | Women | 30-39 | Middle | 0.186794520 | 0.154756436 | 0.223709767 |
| Lesotho, 2014 | Women | 30-39 | Poorer | 0.151331600 | 0.123572705 | 0.184016893 |
| Lesotho, 2014 | Women | 30-39 | Poorest | 0.137108185 | 0.111860233 | 0.166983405 |
| Lesotho, 2014 | Women | 30-39 | Richer | 0.217274087 | 0.178592013 | 0.261665512 |
| Lesotho, 2014 | Women | 30-39 | Richest | 0.307491608 | 0.264550808 | 0.354047019 |
| Lesotho, 2014 | Women | 40-49 | Middle | 0.199267683 | 0.160754588 | 0.244321503 |
| Lesotho, 2014 | Women | 40-49 | Poorer | 0.154294184 | 0.118951164 | 0.197781050 |
| Lesotho, 2014 | Women | 40-49 | Poorest | 0.138145809 | 0.107107088 | 0.176402259 |
| Lesotho, 2014 | Women | 40-49 | Richer | 0.251179898 | 0.208494062 | 0.299300318 |
| Lesotho, 2014 | Women | 40-49 | Richest | 0.257112426 | 0.209206375 | 0.311665005 |
| Mali, 2006 | Women | 18-29 | Middle | 0.169222052 | 0.140005966 | 0.203095078 |
| Mali, 2006 | Women | 18-29 | Poorer | 0.166841324 | 0.137254779 | 0.201317331 |
| Mali, 2006 | Women | 18-29 | Poorest | 0.155755166 | 0.120816218 | 0.198516683 |
| Mali, 2006 | Women | 18-29 | Richer | 0.229135430 | 0.183452526 | 0.282261810 |
| Mali, 2006 | Women | 18-29 | Richest | 0.279046032 | 0.228673161 | 0.335686027 |
| Mali, 2006 | Women | 30-39 | Middle | 0.187564946 | 0.159096263 | 0.219796290 |
| Mali, 2006 | Women | 30-39 | Poorer | 0.220394468 | 0.185546572 | 0.259700299 |
| Mali, 2006 | Women | 30-39 | Poorest | 0.200102815 | 0.164650482 | 0.240986527 |
| Mali, 2006 | Women | 30-39 | Richer | 0.218972378 | 0.178286035 | 0.265938705 |
| Mali, 2006 | Women | 30-39 | Richest | 0.172965393 | 0.132876986 | 0.222051163 |
| Mali, 2006 | Women | 40-49 | Middle | 0.160910918 | 0.129057396 | 0.198831572 |
| Mali, 2006 | Women | 40-49 | Poorer | 0.220531302 | 0.181991143 | 0.264593127 |
| Mali, 2006 | Women | 40-49 | Poorest | 0.226207900 | 0.183909794 | 0.274956653 |
| Mali, 2006 | Women | 40-49 | Richer | 0.192480115 | 0.149754586 | 0.243898681 |
| Mali, 2006 | Women | 40-49 | Richest | 0.199869766 | 0.148665234 | 0.263256949 |
| Mali, 2012 | Women | 18-29 | Middle | 0.184984612 | 0.162387695 | 0.209937862 |
| Mali, 2012 | Women | 18-29 | Poorer | 0.172770048 | 0.150135298 | 0.198022146 |
| Mali, 2012 | Women | 18-29 | Poorest | 0.188503535 | 0.159870189 | 0.220916695 |
| Mali, 2012 | Women | 18-29 | Richer | 0.215385487 | 0.182096042 | 0.252879079 |
| Mali, 2012 | Women | 18-29 | Richest | 0.238356319 | 0.202958477 | 0.277776278 |
| Mali, 2012 | Women | 30-39 | Middle | 0.182001468 | 0.157658927 | 0.209169194 |
| Mali, 2012 | Women | 30-39 | Poorer | 0.196298758 | 0.167940084 | 0.228133170 |
| Mali, 2012 | Women | 30-39 | Poorest | 0.206090082 | 0.175458180 | 0.240509881 |
| Mali, 2012 | Women | 30-39 | Richer | 0.197099689 | 0.166183253 | 0.232166289 |
| Mali, 2012 | Women | 30-39 | Richest | 0.218510004 | 0.183224929 | 0.258440122 |
| Mali, 2012 | Women | 40-49 | Middle | 0.225321906 | 0.185544513 | 0.270791596 |
| Mali, 2012 | Women | 40-49 | Poorer | 0.186984186 | 0.152643706 | 0.226980834 |
| Mali, 2012 | Women | 40-49 | Poorest | 0.233980325 | 0.189305115 | 0.285485926 |
| Mali, 2012 | Women | 40-49 | Richer | 0.178430745 | 0.143332542 | 0.219917196 |
| Mali, 2012 | Women | 40-49 | Richest | 0.175282839 | 0.138699954 | 0.219060579 |
| Malawi, 2004 | Women | 18-29 | Middle | 0.213279301 | 0.185117915 | 0.244439687 |
| Malawi, 2004 | Women | 18-29 | Poorer | 0.215038197 | 0.185315677 | 0.248076244 |
| Malawi, 2004 | Women | 18-29 | Poorest | 0.180240601 | 0.154207762 | 0.209579234 |
| Malawi, 2004 | Women | 18-29 | Richer | 0.193851865 | 0.166203161 | 0.224859665 |
| Malawi, 2004 | Women | 18-29 | Richest | 0.197590036 | 0.159905793 | 0.241601115 |
| Malawi, 2004 | Women | 30-39 | Middle | 0.229174357 | 0.191472234 | 0.271804599 |
| Malawi, 2004 | Women | 30-39 | Poorer | 0.233911579 | 0.195589313 | 0.277155378 |
| Malawi, 2004 | Women | 30-39 | Poorest | 0.157179532 | 0.126207209 | 0.194064614 |
| Malawi, 2004 | Women | 30-39 | Richer | 0.228341446 | 0.188661092 | 0.273553711 |
| Malawi, 2004 | Women | 30-39 | Richest | 0.151393086 | 0.117626124 | 0.192736198 |
| Malawi, 2004 | Women | 40-49 | Middle | 0.203676587 | 0.163724967 | 0.250457367 |
| Malawi, 2004 | Women | 40-49 | Poorer | 0.178160363 | 0.136173479 | 0.229651487 |
| Malawi, 2004 | Women | 40-49 | Poorest | 0.201530871 | 0.159752870 | 0.250971158 |
| Malawi, 2004 | Women | 40-49 | Richer | 0.227103648 | 0.183295105 | 0.277820900 |
| Malawi, 2004 | Women | 40-49 | Richest | 0.189528530 | 0.142820632 | 0.247108156 |
| Malawi, 2010 | Women | 18-29 | Middle | 0.192577799 | 0.174081603 | 0.212533508 |
| Malawi, 2010 | Women | 18-29 | Poorer | 0.199276126 | 0.179371375 | 0.220795399 |
| Malawi, 2010 | Women | 18-29 | Poorest | 0.175637969 | 0.155635315 | 0.197609763 |
| Malawi, 2010 | Women | 18-29 | Richer | 0.177049670 | 0.159549536 | 0.196021608 |
| Malawi, 2010 | Women | 18-29 | Richest | 0.255458436 | 0.223238203 | 0.290589329 |
| Malawi, 2010 | Women | 30-39 | Middle | 0.187936802 | 0.164431918 | 0.213941329 |
| Malawi, 2010 | Women | 30-39 | Poorer | 0.186103109 | 0.162845534 | 0.211841786 |
| Malawi, 2010 | Women | 30-39 | Poorest | 0.161883626 | 0.140591409 | 0.185703703 |
| Malawi, 2010 | Women | 30-39 | Richer | 0.201960452 | 0.179595228 | 0.226342443 |
| Malawi, 2010 | Women | 30-39 | Richest | 0.262116011 | 0.226165150 | 0.301554615 |
| Malawi, 2010 | Women | 40-49 | Middle | 0.177882260 | 0.151488236 | 0.207749020 |
| Malawi, 2010 | Women | 40-49 | Poorer | 0.232673350 | 0.202204446 | 0.266201478 |
| Malawi, 2010 | Women | 40-49 | Poorest | 0.164329064 | 0.138832177 | 0.193456606 |
| Malawi, 2010 | Women | 40-49 | Richer | 0.216412762 | 0.187533799 | 0.248379349 |
| Malawi, 2010 | Women | 40-49 | Richest | 0.208702565 | 0.176487530 | 0.245048165 |
| Malawi, 2015 | Women | 18-29 | Middle | 0.179633835 | 0.163063500 | 0.197490690 |
| Malawi, 2015 | Women | 18-29 | Poorer | 0.210660239 | 0.191605883 | 0.231067844 |
| Malawi, 2015 | Women | 18-29 | Poorest | 0.199866972 | 0.182762150 | 0.218145330 |
| Malawi, 2015 | Women | 18-29 | Richer | 0.172076703 | 0.154131252 | 0.191638170 |
| Malawi, 2015 | Women | 18-29 | Richest | 0.237762251 | 0.213246974 | 0.264149609 |
| Malawi, 2015 | Women | 30-39 | Middle | 0.194060511 | 0.173998777 | 0.215830927 |
| Malawi, 2015 | Women | 30-39 | Poorer | 0.180527469 | 0.159957806 | 0.203102740 |
| Malawi, 2015 | Women | 30-39 | Poorest | 0.173136426 | 0.154671428 | 0.193301735 |
| Malawi, 2015 | Women | 30-39 | Richer | 0.207438321 | 0.185863845 | 0.230807127 |
| Malawi, 2015 | Women | 30-39 | Richest | 0.244837273 | 0.215040655 | 0.277304038 |
| Malawi, 2015 | Women | 40-49 | Middle | 0.211994388 | 0.182332275 | 0.245035898 |
| Malawi, 2015 | Women | 40-49 | Poorer | 0.157356981 | 0.132859766 | 0.185405322 |
| Malawi, 2015 | Women | 40-49 | Poorest | 0.198968927 | 0.170992242 | 0.230251648 |
| Malawi, 2015 | Women | 40-49 | Richer | 0.227858338 | 0.199110855 | 0.259411980 |
| Malawi, 2015 | Women | 40-49 | Richest | 0.203821366 | 0.174287453 | 0.236923948 |
| Niger, 2006 | Women | 18-29 | Middle | 0.196112402 | 0.169748556 | 0.225458939 |
| Niger, 2006 | Women | 18-29 | Poorer | 0.194749811 | 0.164562055 | 0.228957646 |
| Niger, 2006 | Women | 18-29 | Poorest | 0.172767180 | 0.145210618 | 0.204303262 |
| Niger, 2006 | Women | 18-29 | Richer | 0.199297481 | 0.164533912 | 0.239302244 |
| Niger, 2006 | Women | 18-29 | Richest | 0.237073125 | 0.193097788 | 0.287495022 |
| Niger, 2006 | Women | 30-39 | Middle | 0.216244339 | 0.182016520 | 0.254902873 |
| Niger, 2006 | Women | 30-39 | Poorer | 0.205230517 | 0.169521154 | 0.246231740 |
| Niger, 2006 | Women | 30-39 | Poorest | 0.203574792 | 0.170447206 | 0.241268348 |
| Niger, 2006 | Women | 30-39 | Richer | 0.184477837 | 0.149782984 | 0.225081661 |
| Niger, 2006 | Women | 30-39 | Richest | 0.190472515 | 0.154291060 | 0.232802966 |
| Niger, 2006 | Women | 40-49 | Middle | 0.262005661 | 0.218795614 | 0.310359040 |
| Niger, 2006 | Women | 40-49 | Poorer | 0.189485109 | 0.152011389 | 0.233651421 |
| Niger, 2006 | Women | 40-49 | Poorest | 0.167747256 | 0.135124031 | 0.206367408 |
| Niger, 2006 | Women | 40-49 | Richer | 0.193200343 | 0.154828075 | 0.238400172 |
| Niger, 2006 | Women | 40-49 | Richest | 0.187561630 | 0.147879508 | 0.234956012 |
| Niger, 2012 | Women | 18-29 | Middle | 0.202634679 | 0.174728876 | 0.233735027 |
| Niger, 2012 | Women | 18-29 | Poorer | 0.184487356 | 0.161181204 | 0.210318524 |
| Niger, 2012 | Women | 18-29 | Poorest | 0.172816259 | 0.148590013 | 0.200064233 |
| Niger, 2012 | Women | 18-29 | Richer | 0.221067915 | 0.192984382 | 0.251962276 |
| Niger, 2012 | Women | 18-29 | Richest | 0.218993791 | 0.185248950 | 0.256947020 |
| Niger, 2012 | Women | 30-39 | Middle | 0.189689467 | 0.161464327 | 0.221544991 |
| Niger, 2012 | Women | 30-39 | Poorer | 0.194990873 | 0.165945931 | 0.227731406 |
| Niger, 2012 | Women | 30-39 | Poorest | 0.192185229 | 0.160968032 | 0.227812711 |
| Niger, 2012 | Women | 30-39 | Richer | 0.211335414 | 0.183073664 | 0.242664064 |
| Niger, 2012 | Women | 30-39 | Richest | 0.211799018 | 0.179204868 | 0.248526445 |
| Niger, 2012 | Women | 40-49 | Middle | 0.203368598 | 0.169335063 | 0.242247503 |
| Niger, 2012 | Women | 40-49 | Poorer | 0.211034478 | 0.173753764 | 0.253856555 |
| Niger, 2012 | Women | 40-49 | Poorest | 0.195800984 | 0.156920492 | 0.241554831 |
| Niger, 2012 | Women | 40-49 | Richer | 0.201353640 | 0.162385190 | 0.246916880 |
| Niger, 2012 | Women | 40-49 | Richest | 0.188442299 | 0.151471950 | 0.231969314 |
| Sierra Leone, 2008 | Women | 18-29 | Middle | 0.200721711 | 0.167858016 | 0.238177951 |
| Sierra Leone, 2008 | Women | 18-29 | Poorer | 0.170938999 | 0.143959918 | 0.201782350 |
| Sierra Leone, 2008 | Women | 18-29 | Poorest | 0.180476275 | 0.148795394 | 0.217181464 |
| Sierra Leone, 2008 | Women | 18-29 | Richer | 0.220076142 | 0.186417804 | 0.257885306 |
| Sierra Leone, 2008 | Women | 18-29 | Richest | 0.227786873 | 0.191101695 | 0.269170962 |
| Sierra Leone, 2008 | Women | 30-39 | Middle | 0.198106332 | 0.165735215 | 0.235018973 |
| Sierra Leone, 2008 | Women | 30-39 | Poorer | 0.210074821 | 0.179084301 | 0.244828820 |
| Sierra Leone, 2008 | Women | 30-39 | Poorest | 0.204112755 | 0.168619584 | 0.244876437 |
| Sierra Leone, 2008 | Women | 30-39 | Richer | 0.194977178 | 0.161972651 | 0.232838371 |
| Sierra Leone, 2008 | Women | 30-39 | Richest | 0.192728915 | 0.154492369 | 0.237767679 |
| Sierra Leone, 2008 | Women | 40-49 | Middle | 0.188932426 | 0.148982674 | 0.236616210 |
| Sierra Leone, 2008 | Women | 40-49 | Poorer | 0.239171375 | 0.195918354 | 0.288546712 |
| Sierra Leone, 2008 | Women | 40-49 | Poorest | 0.189493897 | 0.149267418 | 0.237534258 |
| Sierra Leone, 2008 | Women | 40-49 | Richer | 0.195866339 | 0.156791687 | 0.241885475 |
| Sierra Leone, 2008 | Women | 40-49 | Richest | 0.186535964 | 0.145425314 | 0.236058047 |
| Sierra Leone, 2013 | Men | 18-29 | Middle | 0.161312788 | 0.136173500 | 0.190071909 |
| Sierra Leone, 2013 | Men | 18-29 | Poorer | 0.167224683 | 0.141601326 | 0.196423708 |
| Sierra Leone, 2013 | Men | 18-29 | Poorest | 0.149852387 | 0.123311135 | 0.180927365 |
| Sierra Leone, 2013 | Men | 18-29 | Richer | 0.194871376 | 0.165035277 | 0.228624491 |
| Sierra Leone, 2013 | Men | 18-29 | Richest | 0.326738767 | 0.267108911 | 0.392550403 |
| Sierra Leone, 2013 | Men | 30-39 | Middle | 0.193805116 | 0.163629465 | 0.228028398 |
| Sierra Leone, 2013 | Men | 30-39 | Poorer | 0.210347582 | 0.183675579 | 0.239755160 |
| Sierra Leone, 2013 | Men | 30-39 | Poorest | 0.225908363 | 0.192172480 | 0.263633832 |
| Sierra Leone, 2013 | Men | 30-39 | Richer | 0.149793317 | 0.126672566 | 0.176282324 |
| Sierra Leone, 2013 | Men | 30-39 | Richest | 0.220145623 | 0.175722191 | 0.272092363 |
| Sierra Leone, 2013 | Men | 40-49 | Middle | 0.220325517 | 0.192602591 | 0.250799315 |
| Sierra Leone, 2013 | Men | 40-49 | Poorer | 0.207156474 | 0.179179676 | 0.238233685 |
| Sierra Leone, 2013 | Men | 40-49 | Poorest | 0.214517062 | 0.182832153 | 0.250013004 |
| Sierra Leone, 2013 | Men | 40-49 | Richer | 0.172942942 | 0.145043504 | 0.204922610 |
| Sierra Leone, 2013 | Men | 40-49 | Richest | 0.185058005 | 0.147641206 | 0.229405214 |
| Sierra Leone, 2013 | Men | 50-59 | Middle | 0.215229896 | 0.177386003 | 0.258609314 |
| Sierra Leone, 2013 | Men | 50-59 | Poorer | 0.228988802 | 0.191447111 | 0.271420973 |
| Sierra Leone, 2013 | Men | 50-59 | Poorest | 0.182038674 | 0.147149347 | 0.223036944 |
| Sierra Leone, 2013 | Men | 50-59 | Richer | 0.205578955 | 0.167485797 | 0.249737057 |
| Sierra Leone, 2013 | Men | 50-59 | Richest | 0.168163673 | 0.116798172 | 0.236080498 |
| Sierra Leone, 2013 | Women | 18-29 | Middle | 0.166404956 | 0.140513747 | 0.195979087 |
| Sierra Leone, 2013 | Women | 18-29 | Poorer | 0.166296808 | 0.142927746 | 0.192628021 |
| Sierra Leone, 2013 | Women | 18-29 | Poorest | 0.172033543 | 0.142861773 | 0.205732312 |
| Sierra Leone, 2013 | Women | 18-29 | Richer | 0.205141421 | 0.178485385 | 0.234641377 |
| Sierra Leone, 2013 | Women | 18-29 | Richest | 0.290123272 | 0.235105366 | 0.352089642 |
| Sierra Leone, 2013 | Women | 30-39 | Middle | 0.226138098 | 0.194953128 | 0.260696082 |
| Sierra Leone, 2013 | Women | 30-39 | Poorer | 0.207546848 | 0.182285555 | 0.235301519 |
| Sierra Leone, 2013 | Women | 30-39 | Poorest | 0.194955871 | 0.165191171 | 0.228614973 |
| Sierra Leone, 2013 | Women | 30-39 | Richer | 0.173316563 | 0.151659039 | 0.197347404 |
| Sierra Leone, 2013 | Women | 30-39 | Richest | 0.198042620 | 0.157717405 | 0.245670941 |
| Sierra Leone, 2013 | Women | 40-49 | Middle | 0.222637360 | 0.193359094 | 0.254947725 |
| Sierra Leone, 2013 | Women | 40-49 | Poorer | 0.213753298 | 0.184542064 | 0.246192416 |
| Sierra Leone, 2013 | Women | 40-49 | Poorest | 0.187701697 | 0.160434273 | 0.218397946 |
| Sierra Leone, 2013 | Women | 40-49 | Richer | 0.201457013 | 0.170968304 | 0.235836077 |
| Sierra Leone, 2013 | Women | 40-49 | Richest | 0.174450632 | 0.131109163 | 0.228354265 |
| Sierra Leone, 2019 | Men | 18-29 | Middle | 0.164870987 | 0.144025014 | 0.188071246 |
| Sierra Leone, 2019 | Men | 18-29 | Poorer | 0.159805052 | 0.138925807 | 0.183154793 |
| Sierra Leone, 2019 | Men | 18-29 | Poorest | 0.145127292 | 0.125558968 | 0.167162333 |
| Sierra Leone, 2019 | Men | 18-29 | Richer | 0.257500305 | 0.227234253 | 0.290283288 |
| Sierra Leone, 2019 | Men | 18-29 | Richest | 0.272696367 | 0.242400435 | 0.305253122 |
| Sierra Leone, 2019 | Men | 30-39 | Middle | 0.147400918 | 0.126186888 | 0.171481472 |
| Sierra Leone, 2019 | Men | 30-39 | Poorer | 0.184360814 | 0.160639403 | 0.210705795 |
| Sierra Leone, 2019 | Men | 30-39 | Poorest | 0.197119273 | 0.165711858 | 0.232818183 |
| Sierra Leone, 2019 | Men | 30-39 | Richer | 0.198973100 | 0.170793694 | 0.230509390 |
| Sierra Leone, 2019 | Men | 30-39 | Richest | 0.272145895 | 0.235687022 | 0.311942823 |
| Sierra Leone, 2019 | Men | 40-49 | Middle | 0.212688734 | 0.185656735 | 0.242484661 |
| Sierra Leone, 2019 | Men | 40-49 | Poorer | 0.187414476 | 0.161783831 | 0.216059016 |
| Sierra Leone, 2019 | Men | 40-49 | Poorest | 0.241759865 | 0.210496996 | 0.276042443 |
| Sierra Leone, 2019 | Men | 40-49 | Richer | 0.177659073 | 0.148210595 | 0.211505868 |
| Sierra Leone, 2019 | Men | 40-49 | Richest | 0.180477852 | 0.150751795 | 0.214584384 |
| Sierra Leone, 2019 | Men | 50-59 | Middle | 0.208158115 | 0.173887158 | 0.247162621 |
| Sierra Leone, 2019 | Men | 50-59 | Poorer | 0.239872473 | 0.203346774 | 0.280647739 |
| Sierra Leone, 2019 | Men | 50-59 | Poorest | 0.224402840 | 0.188320694 | 0.265140029 |
| Sierra Leone, 2019 | Men | 50-59 | Richer | 0.173148764 | 0.137282099 | 0.216039478 |
| Sierra Leone, 2019 | Men | 50-59 | Richest | 0.154417808 | 0.117616196 | 0.200122894 |
| Sierra Leone, 2019 | Women | 18-29 | Middle | 0.177868871 | 0.157642671 | 0.200073795 |
| Sierra Leone, 2019 | Women | 18-29 | Poorer | 0.159137863 | 0.142167913 | 0.177713800 |
| Sierra Leone, 2019 | Women | 18-29 | Poorest | 0.136356777 | 0.117530218 | 0.157660281 |
| Sierra Leone, 2019 | Women | 18-29 | Richer | 0.247229195 | 0.221890598 | 0.274440760 |
| Sierra Leone, 2019 | Women | 18-29 | Richest | 0.279407295 | 0.252940775 | 0.307503238 |
| Sierra Leone, 2019 | Women | 30-39 | Middle | 0.191160755 | 0.170792329 | 0.213333342 |
| Sierra Leone, 2019 | Women | 30-39 | Poorer | 0.187313411 | 0.166886020 | 0.209612084 |
| Sierra Leone, 2019 | Women | 30-39 | Poorest | 0.224093163 | 0.199647272 | 0.250595126 |
| Sierra Leone, 2019 | Women | 30-39 | Richer | 0.177821295 | 0.154502566 | 0.203811088 |
| Sierra Leone, 2019 | Women | 30-39 | Richest | 0.219611377 | 0.190964394 | 0.251221330 |
| Sierra Leone, 2019 | Women | 40-49 | Middle | 0.209747236 | 0.182219382 | 0.240212176 |
| Sierra Leone, 2019 | Women | 40-49 | Poorer | 0.224228228 | 0.197221236 | 0.253764441 |
| Sierra Leone, 2019 | Women | 40-49 | Poorest | 0.227716319 | 0.199037584 | 0.259190103 |
| Sierra Leone, 2019 | Women | 40-49 | Richer | 0.195082082 | 0.164643503 | 0.229601313 |
| Sierra Leone, 2019 | Women | 40-49 | Richest | 0.143226135 | 0.117903131 | 0.172921765 |
| Zimbabwe, 2005 | Women | 18-29 | Middle | 0.164054878 | 0.138301936 | 0.193526241 |
| Zimbabwe, 2005 | Women | 18-29 | Poorer | 0.173865147 | 0.149758999 | 0.200934558 |
| Zimbabwe, 2005 | Women | 18-29 | Poorest | 0.170985331 | 0.127166484 | 0.225993777 |
| Zimbabwe, 2005 | Women | 18-29 | Richer | 0.225775461 | 0.198329105 | 0.255808066 |
| Zimbabwe, 2005 | Women | 18-29 | Richest | 0.265319184 | 0.223008190 | 0.312429864 |
| Zimbabwe, 2005 | Women | 30-39 | Middle | 0.174595767 | 0.148070020 | 0.204731468 |
| Zimbabwe, 2005 | Women | 30-39 | Poorer | 0.159655431 | 0.137372576 | 0.184778502 |
| Zimbabwe, 2005 | Women | 30-39 | Poorest | 0.185616190 | 0.154572274 | 0.221263139 |
| Zimbabwe, 2005 | Women | 30-39 | Richer | 0.240905175 | 0.210090587 | 0.274667847 |
| Zimbabwe, 2005 | Women | 30-39 | Richest | 0.239227436 | 0.199102204 | 0.284565947 |
| Zimbabwe, 2005 | Women | 40-49 | Middle | 0.217204423 | 0.182767440 | 0.256096674 |
| Zimbabwe, 2005 | Women | 40-49 | Poorer | 0.187750780 | 0.162393237 | 0.216046579 |
| Zimbabwe, 2005 | Women | 40-49 | Poorest | 0.224647386 | 0.184553354 | 0.270561735 |
| Zimbabwe, 2005 | Women | 40-49 | Richer | 0.180773542 | 0.152980411 | 0.212350169 |
| Zimbabwe, 2005 | Women | 40-49 | Richest | 0.189623869 | 0.149993333 | 0.236808225 |
| Zimbabwe, 2010 | Men | 18-29 | Middle | 0.205827329 | 0.181673295 | 0.232281178 |
| Zimbabwe, 2010 | Men | 18-29 | Poorer | 0.167162805 | 0.146843177 | 0.189669095 |
| Zimbabwe, 2010 | Men | 18-29 | Poorest | 0.138598389 | 0.118680121 | 0.161247945 |
| Zimbabwe, 2010 | Men | 18-29 | Richer | 0.247751416 | 0.220399219 | 0.277290739 |
| Zimbabwe, 2010 | Men | 18-29 | Richest | 0.240660061 | 0.207656251 | 0.277075040 |
| Zimbabwe, 2010 | Men | 30-39 | Middle | 0.175356431 | 0.150956517 | 0.202758401 |
| Zimbabwe, 2010 | Men | 30-39 | Poorer | 0.184604311 | 0.159188287 | 0.213050039 |
| Zimbabwe, 2010 | Men | 30-39 | Poorest | 0.194833326 | 0.168103287 | 0.224665834 |
| Zimbabwe, 2010 | Men | 30-39 | Richer | 0.223913333 | 0.198289887 | 0.251807912 |
| Zimbabwe, 2010 | Men | 30-39 | Richest | 0.221292600 | 0.187076594 | 0.259766917 |
| Zimbabwe, 2010 | Men | 40-49 | Middle | 0.193637859 | 0.158960961 | 0.233776738 |
| Zimbabwe, 2010 | Men | 40-49 | Poorer | 0.186456986 | 0.157114083 | 0.219850633 |
| Zimbabwe, 2010 | Men | 40-49 | Poorest | 0.194078465 | 0.161720783 | 0.231125350 |
| Zimbabwe, 2010 | Men | 40-49 | Richer | 0.183472833 | 0.152132684 | 0.219597060 |
| Zimbabwe, 2010 | Men | 40-49 | Richest | 0.242353857 | 0.200178828 | 0.290190693 |
| Zimbabwe, 2010 | Men | 50-59 | Middle | 0.157779838 | 0.120762358 | 0.203517841 |
| Zimbabwe, 2010 | Men | 50-59 | Poorer | 0.163549128 | 0.121498742 | 0.216565350 |
| Zimbabwe, 2010 | Men | 50-59 | Poorest | 0.151516409 | 0.115265988 | 0.196633572 |
| Zimbabwe, 2010 | Men | 50-59 | Richer | 0.246542661 | 0.193514799 | 0.308542275 |
| Zimbabwe, 2010 | Men | 50-59 | Richest | 0.280611965 | 0.223024205 | 0.346439414 |
| Zimbabwe, 2010 | Women | 18-29 | Middle | 0.177618952 | 0.156283603 | 0.201172458 |
| Zimbabwe, 2010 | Women | 18-29 | Poorer | 0.168797358 | 0.152037367 | 0.186997469 |
| Zimbabwe, 2010 | Women | 18-29 | Poorest | 0.167469294 | 0.147252417 | 0.189843893 |
| Zimbabwe, 2010 | Women | 18-29 | Richer | 0.229489915 | 0.206994004 | 0.253648663 |
| Zimbabwe, 2010 | Women | 18-29 | Richest | 0.256624484 | 0.224926059 | 0.291112262 |
| Zimbabwe, 2010 | Women | 30-39 | Middle | 0.199168461 | 0.176287366 | 0.224211014 |
| Zimbabwe, 2010 | Women | 30-39 | Poorer | 0.173440801 | 0.153781170 | 0.195034487 |
| Zimbabwe, 2010 | Women | 30-39 | Poorest | 0.185497633 | 0.162796647 | 0.210567963 |
| Zimbabwe, 2010 | Women | 30-39 | Richer | 0.235100418 | 0.210637982 | 0.261462782 |
| Zimbabwe, 2010 | Women | 30-39 | Richest | 0.206792688 | 0.178695364 | 0.238027536 |
| Zimbabwe, 2010 | Women | 40-49 | Middle | 0.195961415 | 0.171320468 | 0.223191911 |
| Zimbabwe, 2010 | Women | 40-49 | Poorer | 0.190941943 | 0.165732687 | 0.218979237 |
| Zimbabwe, 2010 | Women | 40-49 | Poorest | 0.183719162 | 0.159671427 | 0.210481505 |
| Zimbabwe, 2010 | Women | 40-49 | Richer | 0.196999404 | 0.170425769 | 0.226584805 |
| Zimbabwe, 2010 | Women | 40-49 | Richest | 0.232378076 | 0.197581343 | 0.271231552 |
| Zimbabwe, 2015 | Men | 18-29 | Middle | 0.200176305 | 0.180861413 | 0.220997405 |
| Zimbabwe, 2015 | Men | 18-29 | Poorer | 0.176066845 | 0.158132383 | 0.195562835 |
| Zimbabwe, 2015 | Men | 18-29 | Poorest | 0.142128189 | 0.121990043 | 0.164966133 |
| Zimbabwe, 2015 | Men | 18-29 | Richer | 0.239207748 | 0.213993073 | 0.266386537 |
| Zimbabwe, 2015 | Men | 18-29 | Richest | 0.242420916 | 0.213703271 | 0.273654582 |
| Zimbabwe, 2015 | Men | 30-39 | Middle | 0.157234227 | 0.136371734 | 0.180620821 |
| Zimbabwe, 2015 | Men | 30-39 | Poorer | 0.186143521 | 0.163008106 | 0.211731865 |
| Zimbabwe, 2015 | Men | 30-39 | Poorest | 0.188368222 | 0.163170566 | 0.216450565 |
| Zimbabwe, 2015 | Men | 30-39 | Richer | 0.246825691 | 0.211605462 | 0.285783118 |
| Zimbabwe, 2015 | Men | 30-39 | Richest | 0.221428338 | 0.189808816 | 0.256646674 |
| Zimbabwe, 2015 | Men | 40-49 | Middle | 0.175431218 | 0.147799192 | 0.206974565 |
| Zimbabwe, 2015 | Men | 40-49 | Poorer | 0.173274945 | 0.148274659 | 0.201493274 |
| Zimbabwe, 2015 | Men | 40-49 | Poorest | 0.145167854 | 0.118968623 | 0.175984208 |
| Zimbabwe, 2015 | Men | 40-49 | Richer | 0.211274264 | 0.176698530 | 0.250556651 |
| Zimbabwe, 2015 | Men | 40-49 | Richest | 0.294851720 | 0.250396382 | 0.343582068 |
| Zimbabwe, 2015 | Men | 50-59 | Middle | 0.195593278 | 0.144099270 | 0.259900978 |
| Zimbabwe, 2015 | Men | 50-59 | Poorer | 0.194024875 | 0.147577129 | 0.250790406 |
| Zimbabwe, 2015 | Men | 50-59 | Poorest | 0.149284139 | 0.112001581 | 0.196234613 |
| Zimbabwe, 2015 | Men | 50-59 | Richer | 0.202700597 | 0.157305130 | 0.257198051 |
| Zimbabwe, 2015 | Men | 50-59 | Richest | 0.258397111 | 0.202530563 | 0.323424153 |
| Zimbabwe, 2015 | Women | 18-29 | Middle | 0.158461580 | 0.140530582 | 0.178206099 |
| Zimbabwe, 2015 | Women | 18-29 | Poorer | 0.167108432 | 0.149958988 | 0.185790445 |
| Zimbabwe, 2015 | Women | 18-29 | Poorest | 0.167218292 | 0.144612828 | 0.192561902 |
| Zimbabwe, 2015 | Women | 18-29 | Richer | 0.247718894 | 0.220420497 | 0.277195991 |
| Zimbabwe, 2015 | Women | 18-29 | Richest | 0.259492801 | 0.229469902 | 0.291955418 |
| Zimbabwe, 2015 | Women | 30-39 | Middle | 0.161949097 | 0.140957088 | 0.185392656 |
| Zimbabwe, 2015 | Women | 30-39 | Poorer | 0.161432682 | 0.142942294 | 0.181807525 |
| Zimbabwe, 2015 | Women | 30-39 | Poorest | 0.185049427 | 0.160036629 | 0.212980329 |
| Zimbabwe, 2015 | Women | 30-39 | Richer | 0.240159091 | 0.211439211 | 0.271437197 |
| Zimbabwe, 2015 | Women | 30-39 | Richest | 0.251409703 | 0.221781351 | 0.283553988 |
| Zimbabwe, 2015 | Women | 40-49 | Middle | 0.238833230 | 0.211570312 | 0.268413245 |
| Zimbabwe, 2015 | Women | 40-49 | Poorer | 0.171980460 | 0.148763496 | 0.197978092 |
| Zimbabwe, 2015 | Women | 40-49 | Poorest | 0.158063918 | 0.134599405 | 0.184745717 |
| Zimbabwe, 2015 | Women | 40-49 | Richer | 0.203686610 | 0.175430702 | 0.235195462 |
| Zimbabwe, 2015 | Women | 40-49 | Richest | 0.227435782 | 0.198675855 | 0.259013252 |

#
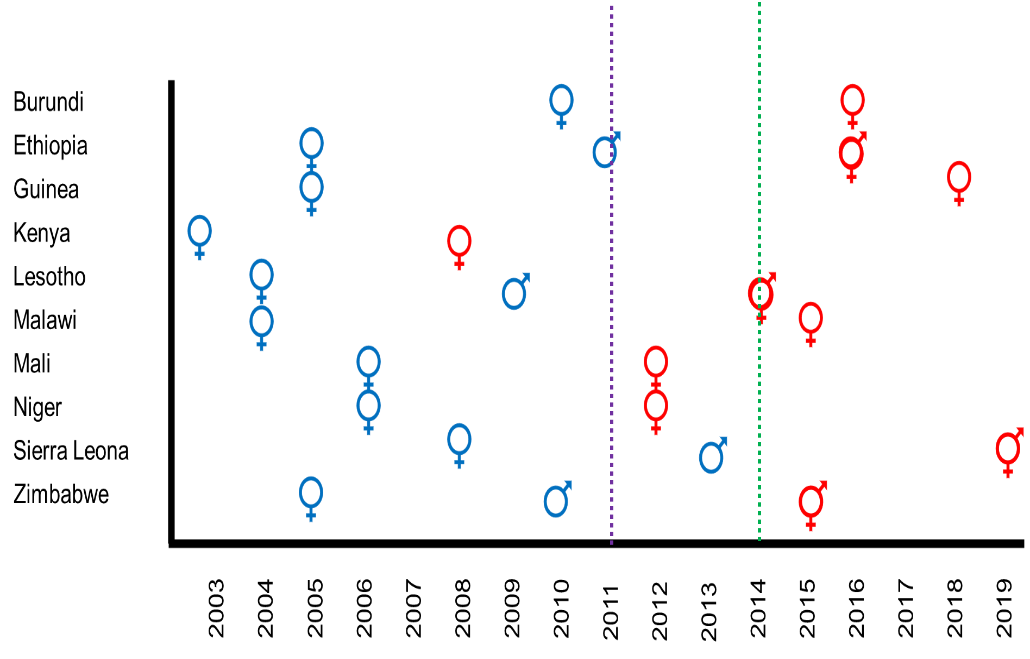
**Table E in S1 text. Distribution of DHS in earliest (blue font) or latest (red font) periods.**

| **Region** | **Country** | **Men** | **Women** |
| --- | --- | --- | --- |
| East | Burundi |  | 2010 2016 |
| East | Ethiopia | 2011 2016 | 2005 2011 2016 |
| West | Guinea |  | 2005 2012 2018 |
| East | Kenya |  | 2003 2008 |
| Southern | Lesotho | 2009 2014 | 2004 2009 2014 |
| East | Malawi |  | 2004 2010 2015 |
| West | Mali |  | 2006 2012 |
| West | Niger |  | 2006 2012 |
| West | Sierra Leona | 2013 2019 | 2008 2013 2019 |
| Southern | Zimbabwe | 2010 2015 | 2005 2010 2015 |

Missing because of the six surveys that were in between the earliest and latest surveys. As shown above, there are six surveys (all for women) that were neither earliest nor latest.

|  | **Absolute number of observations** | |
| --- | --- | --- |
|  | **Men** | **Women** |
| **2011 or before** |  | 33,563 |
| **2012 or later** |  | 55,040 |
| **2013 or before** | 24,827 |  |
| **2014 or later** | 23,653 |  |
| **Missing** |  | 36,730 |

Years and male/female symbols in blue color were included in the earliest period, and years as well as male/female symbols in red color were included in the latest period. Almost organically, the surveys in the earliest and latest periods were divided in 2011 (purple vertical dotted line) for women and in 2014 (green vertical dotted line) for men. For **women (purple vertical dotted line)**, except in Kenya where both surveys were conducted before 2011, all the other countries have one survey before 2011 and another survey in 2012 or after. For **men (green vertical dotted line)**, all other countries have one survey in or before 2013 and another survey in 2014 or after. ***Thus, hereafter, and in the manuscript, we refer to the study periods as 2011 or before and 2012 or later for women, as well as 2013 or before and 2014 or later for men.***

# **Table F in S1 text. Unweighted description of the study sample.**

|  | **Age** | **Sex** | **Height** | **Weight** | **BMI** | **Overweight** | **HIV test** |
| --- | --- | --- | --- | --- | --- | --- | --- |
| **Burundi_2010** | : | : | : | : | : | : | : |
| **(N=3344)** | Mean (SD): 30.0 (9.13) | Men: 0 (0%) | Mean (SD): 52.2 (9.98) | Mean (SD): 156 (6.97) | Mean (SD): 21.5 (3.68) | <25: 2979 (89.1%) | No: 3253 (97.3%) |
|  | Median [Min, Max]: 28.0 [18.0, 49.0] | Women: 3344 (100%) | Median [Min, Max]: 50.5 [15.6, 149] | Median [Min, Max]: 156 [100, 199] | Median [Min, Max]: 20.9 [10.8, 62.1] | 25+: 365 (10.9%) | Yes: 91 (2.7%) |
| **Burundi_2016** | : | : | : | : | : | : | : |
| **(N=6582)** | Mean (SD): 30.3 (8.86) | Men: 0 (0%) | Mean (SD): 51.6 (9.43) | Mean (SD): 156 (6.37) | Mean (SD): 21.2 (3.32) | <25: 5928 (90.1%) | No: 6468 (98.3%) |
|  | Median [Min, Max]: 29.0 [18.0, 49.0] | Women: 6582 (100%) | Median [Min, Max]: 50.1 [27.1, 117] | Median [Min, Max]: 156 [102, 181] | Median [Min, Max]: 20.7 [10.8, 54.8] | 25+: 654 (9.9%) | Yes: 114 (1.7%) |
| **Ethiopia_2005** | : | : | : | : | : | : | : |
| **(N=4631)** | Mean (SD): 29.9 (8.88) | Men: 0 (0%) | Mean (SD): 50.7 (8.68) | Mean (SD): 157 (6.61) | Mean (SD): 20.5 (3.21) | <25: 4334 (93.6%) | No: 4501 (97.2%) |
|  | Median [Min, Max]: 28.0 [18.0, 49.0] | Women: 4631 (100%) | Median [Min, Max]: 49.5 [27.0, 159] | Median [Min, Max]: 157 [103, 200] | Median [Min, Max]: 20.0 [10.1, 66.6] | 25+: 297 (6.4%) | Yes: 130 (2.8%) |
| **Ethiopia_2011** | : | : | : | : | : | : | : |
| **(N=23436)** | Mean (SD): 31.2 (9.91) | Men: 11345 (48.4%) | Mean (SD): 53.2 (9.29) | Mean (SD): 163 (9.00) | Mean (SD): 20.1 (3.08) | <25: 21966 (93.7%) | No: 22919 (97.8%) |
|  | Median [Min, Max]: 30.0 [18.0, 59.0] | Women: 12091 (51.6%) | Median [Min, Max]: 52.1 [21.1, 173] | Median [Min, Max]: 162 [101, 199] | Median [Min, Max]: 19.6 [10.5, 63.8] | 25+: 1470 (6.3%) | Yes: 517 (2.2%) |
| **Ethiopia_2016** | : | : | : | : | : | : | : |
| **(N=21442)** | Mean (SD): 31.4 (9.82) | Men: 9925 (46.3%) | Mean (SD): 54.7 (10.0) | Mean (SD): 163 (8.93) | Mean (SD): 20.6 (3.39) | <25: 19434 (90.6%) | No: 21064 (98.2%) |
|  | Median [Min, Max]: 30.0 [18.0, 59.0] | Women: 11517 (53.7%) | Median [Min, Max]: 53.3 [22.0, 168] | Median [Min, Max]: 163 [103, 196] | Median [Min, Max]: 19.9 [11.3, 65.8] | 25+: 2008 (9.4%) | Yes: 378 (1.8%) |
| **Guinea_2005** | : | : | : | : | : | : | : |
| **(N=2968)** | Mean (SD): 31.7 (9.05) | Men: 0 (0%) | Mean (SD): 55.0 (9.55) | Mean (SD): 159 (6.41) | Mean (SD): 21.8 (3.47) | <25: 2534 (85.4%) | No: 2906 (97.9%) |
|  | Median [Min, Max]: 30.0 [18.0, 49.0] | Women: 2968 (100%) | Median [Min, Max]: 53.6 [30.5, 107] | Median [Min, Max]: 159 [101, 189] | Median [Min, Max]: 21.2 [12.9, 47.8] | 25+: 434 (14.6%) | Yes: 62 (2.1%) |
| **Guinea_2012** | : | : | : | : | : | : | : |
| **(N=3586)** | Mean (SD): 30.7 (9.08) | Men: 0 (0%) | Mean (SD): 57.5 (11.0) | Mean (SD): 160 (6.49) | Mean (SD): 22.6 (4.12) | <25: 2854 (79.6%) | No: 3496 (97.5%) |
|  | Median [Min, Max]: 30.0 [18.0, 49.0] | Women: 3586 (100%) | Median [Min, Max]: 55.9 [27.3, 144] | Median [Min, Max]: 160 [101, 200] | Median [Min, Max]: 21.8 [12.9, 67.9] | 25+: 732 (20.4%) | Yes: 90 (2.5%) |
| **Guinea_2018** | : | : | : | : | : | : | : |
| **(N=4033)** | Mean (SD): 31.1 (9.16) | Men: 0 (0%) | Mean (SD): 59.8 (12.3) | Mean (SD): 159 (7.24) | Mean (SD): 23.7 (4.92) | <25: 2865 (71.0%) | No: 3957 (98.1%) |
|  | Median [Min, Max]: 30.0 [18.0, 49.0] | Women: 4033 (100%) | Median [Min, Max]: 57.6 [21.5, 126] | Median [Min, Max]: 159 [102, 190] | Median [Min, Max]: 22.7 [11.3, 75.7] | 25+: 1168 (29.0%) | Yes: 76 (1.9%) |
| **Kenya_2003** | : | : | : | : | : | : | : |
| **(N=2551)** | Mean (SD): 30.1 (8.72) | Men: 0 (0%) | Mean (SD): 59.1 (12.1) | Mean (SD): 160 (6.32) | Mean (SD): 23.1 (4.41) | <25: 1864 (73.1%) | No: 2310 (90.6%) |
|  | Median [Min, Max]: 29.0 [18.0, 49.0] | Women: 2551 (100%) | Median [Min, Max]: 56.7 [33.6, 165] | Median [Min, Max]: 160 [140, 194] | Median [Min, Max]: 22.2 [12.9, 60.1] | 25+: 687 (26.9%) | Yes: 241 (9.4%) |
| **Kenya_2008** | : | : | : | : | : | : | : |
| **(N=3077)** | Mean (SD): 30.5 (9.03) | Men: 0 (0%) | Mean (SD): 58.8 (11.9) | Mean (SD): 159 (7.28) | Mean (SD): 23.3 (4.79) | <25: 2211 (71.9%) | No: 2796 (90.9%) |
|  | Median [Min, Max]: 29.0 [18.0, 49.0] | Women: 3077 (100%) | Median [Min, Max]: 57.1 [32.1, 159] | Median [Min, Max]: 159 [102, 207] | Median [Min, Max]: 22.4 [10.6, 75.0] | 25+: 866 (28.1%) | Yes: 281 (9.1%) |
| **Lesotho_2004** | : | : | : | : | : | : | : |
| **(N=2352)** | Mean (SD): 30.6 (9.35) | Men: 0 (0%) | Mean (SD): 62.6 (14.0) | Mean (SD): 157 (6.47) | Mean (SD): 25.3 (5.35) | <25: 1331 (56.6%) | No: 1636 (69.6%) |
|  | Median [Min, Max]: 29.0 [18.0, 49.0] | Women: 2352 (100%) | Median [Min, Max]: 59.9 [32.7, 171] | Median [Min, Max]: 157 [133, 194] | Median [Min, Max]: 24.2 [11.2, 53.7] | 25+: 1021 (43.4%) | Yes: 716 (30.4%) |
| **Lesotho_2009** | : | : | : | : | : | : | : |
| **(N=5676)** | Mean (SD): 31.2 (10.4) | Men: 2575 (45.4%) | Mean (SD): 61.8 (13.0) | Mean (SD): 162 (8.45) | Mean (SD): 23.6 (5.16) | <25: 4026 (70.9%) | No: 4212 (74.2%) |
|  | Median [Min, Max]: 29.0 [18.0, 59.0] | Women: 3101 (54.6%) | Median [Min, Max]: 59.3 [32.2, 178] | Median [Min, Max]: 162 [114, 192] | Median [Min, Max]: 22.3 [12.3, 60.5] | 25+: 1650 (29.1%) | Yes: 1464 (25.8%) |
| **Lesotho_2014** | : | : | : | : | : | : | : |
| **(N=5052)** | Mean (SD): 31.5 (10.4) | Men: 2352 (46.6%) | Mean (SD): 63.6 (13.4) | Mean (SD): 162 (8.26) | Mean (SD): 24.2 (5.27) | <25: 3397 (67.2%) | No: 3674 (72.7%) |
|  | Median [Min, Max]: 30.0 [18.0, 59.0] | Women: 2700 (53.4%) | Median [Min, Max]: 61.1 [31.5, 157] | Median [Min, Max]: 162 [121, 188] | Median [Min, Max]: 22.7 [14.5, 63.7] | 25+: 1655 (32.8%) | Yes: 1378 (27.3%) |
| **Malawi_2004** | : | : | : | : | : | : | : |
| **(N=2190)** | Mean (SD): 30.0 (8.75) | Men: 0 (0%) | Mean (SD): 54.0 (9.06) | Mean (SD): 156 (6.27) | Mean (SD): 22.2 (3.39) | <25: 1882 (85.9%) | No: 1819 (83.1%) |
|  | Median [Min, Max]: 28.0 [18.0, 49.0] | Women: 2190 (100%) | Median [Min, Max]: 52.6 [27.5, 153] | Median [Min, Max]: 156 [102, 189] | Median [Min, Max]: 21.6 [12.3, 56.7] | 25+: 308 (14.1%) | Yes: 371 (16.9%) |
| **Malawi_2010** | : | : | : | : | : | : | : |
| **(N=5597)** | Mean (SD): 30.6 (8.70) | Men: 0 (0%) | Mean (SD): 55.1 (9.84) | Mean (SD): 156 (6.34) | Mean (SD): 22.6 (3.73) | <25: 4623 (82.6%) | No: 4801 (85.8%) |
|  | Median [Min, Max]: 29.0 [18.0, 49.0] | Women: 5597 (100%) | Median [Min, Max]: 53.6 [17.4, 158] | Median [Min, Max]: 156 [100, 196] | Median [Min, Max]: 22.0 [10.4, 76.1] | 25+: 974 (17.4%) | Yes: 796 (14.2%) |
| **Malawi_2015** | : | : | : | : | : | : | : |
| **(N=6167)** | Mean (SD): 30.2 (8.62) | Men: 0 (0%) | Mean (SD): 56.6 (10.9) | Mean (SD): 156 (5.87) | Mean (SD): 23.2 (4.20) | <25: 4676 (75.8%) | No: 5384 (87.3%) |
|  | Median [Min, Max]: 29.0 [18.0, 49.0] | Women: 6167 (100%) | Median [Min, Max]: 54.5 [27.0, 163] | Median [Min, Max]: 156 [100, 185] | Median [Min, Max]: 22.3 [10.2, 69.4] | 25+: 1491 (24.2%) | Yes: 783 (12.7%) |
| **Mali_2006** | : | : | : | : | : | : | : |
| **(N=3492)** | Mean (SD): 30.8 (8.97) | Men: 0 (0%) | Mean (SD): 58.7 (11.7) | Mean (SD): 161 (6.55) | Mean (SD): 22.6 (4.19) | <25: 2772 (79.4%) | No: 3436 (98.4%) |
|  | Median [Min, Max]: 30.0 [18.0, 49.0] | Women: 3492 (100%) | Median [Min, Max]: 56.7 [28.9, 135] | Median [Min, Max]: 161 [102, 188] | Median [Min, Max]: 21.7 [11.9, 56.4] | 25+: 720 (20.6%) | Yes: 56 (1.6%) |
| **Mali_2012** | : | : | : | : | : | : | : |
| **(N=4016)** | Mean (SD): 30.4 (8.47) | Men: 0 (0%) | Mean (SD): 60.0 (12.8) | Mean (SD): 162 (6.83) | Mean (SD): 22.8 (4.60) | <25: 3174 (79.0%) | No: 3966 (98.8%) |
|  | Median [Min, Max]: 30.0 [18.0, 49.0] | Women: 4016 (100%) | Median [Min, Max]: 57.6 [33.0, 179] | Median [Min, Max]: 162 [102, 197] | Median [Min, Max]: 21.8 [12.6, 71.1] | 25+: 842 (21.0%) | Yes: 50 (1.2%) |
| **Niger_2006** | : | : | : | : | : | : | : |
| **(N=3297)** | Mean (SD): 30.7 (8.64) | Men: 0 (0%) | Mean (SD): 57.3 (11.8) | Mean (SD): 161 (6.06) | Mean (SD): 22.2 (4.14) | <25: 2653 (80.5%) | No: 3263 (99.0%) |
|  | Median [Min, Max]: 30.0 [18.0, 49.0] | Women: 3297 (100%) | Median [Min, Max]: 55.0 [31.6, 124] | Median [Min, Max]: 161 [125, 187] | Median [Min, Max]: 21.3 [13.5, 51.8] | 25+: 644 (19.5%) | Yes: 34 (1.0%) |
| **Niger_2012** | : | : | : | : | : | : | : |
| **(N=3869)** | Mean (SD): 30.6 (8.37) | Men: 0 (0%) | Mean (SD): 58.6 (12.2) | Mean (SD): 161 (6.30) | Mean (SD): 22.7 (4.49) | <25: 2987 (77.2%) | No: 3845 (99.4%) |
|  | Median [Min, Max]: 30.0 [18.0, 49.0] | Women: 3869 (100%) | Median [Min, Max]: 56.5 [30.0, 169] | Median [Min, Max]: 160 [102, 184] | Median [Min, Max]: 21.9 [13.8, 77.2] | 25+: 882 (22.8%) | Yes: 24 (0.6%) |
| **Sierra Leone_2008** | : | : | : | : | : | : | : |
| **(N=2830)** | Mean (SD): 30.8 (8.30) | Men: 0 (0%) | Mean (SD): 57.1 (11.9) | Mean (SD): 155 (11.8) | Mean (SD): 23.9 (5.75) | <25: 1937 (68.4%) | No: 2773 (98.0%) |
|  | Median [Min, Max]: 30.0 [18.0, 49.0] | Women: 2830 (100%) | Median [Min, Max]: 54.9 [22.6, 150] | Median [Min, Max]: 156 [100, 200] | Median [Min, Max]: 22.9 [10.0, 75.4] | 25+: 893 (31.6%) | Yes: 57 (2.0%) |
| **Sierra Leone_2013** | : | : | : | : | : | : | : |
| **(N=11905)** | Mean (SD): 32.2 (10.2) | Men: 5790 (48.6%) | Mean (SD): 59.0 (10.9) | Mean (SD): 162 (8.11) | Mean (SD): 22.4 (3.87) | <25: 10057 (84.5%) | No: 11713 (98.4%) |
|  | Median [Min, Max]: 31.0 [18.0, 59.0] | Women: 6115 (51.4%) | Median [Min, Max]: 57.9 [23.3, 177] | Median [Min, Max]: 162 [106, 199] | Median [Min, Max]: 21.7 [10.6, 64.8] | 25+: 1848 (15.5%) | Yes: 192 (1.6%) |
| **Sierra Leone_2019** | : | : | : | : | : | : | : |
| **(N=10972)** | Mean (SD): 32.7 (10.5) | Men: 5177 (47.2%) | Mean (SD): 60.1 (10.5) | Mean (SD): 162 (7.98) | Mean (SD): 22.9 (3.84) | <25: 8698 (79.3%) | No: 10765 (98.1%) |
|  | Median [Min, Max]: 31.0 [18.0, 59.0] | Women: 5795 (52.8%) | Median [Min, Max]: 58.8 [31.7, 153] | Median [Min, Max]: 162 [108, 194] | Median [Min, Max]: 22.1 [14.1, 62.8] | 25+: 2274 (20.7%) | Yes: 207 (1.9%) |
| **Zimbabwe_2005** | : | : | : | : | : | : | : |
| **(N=5908)** | Mean (SD): 29.9 (8.89) | Men: 0 (0%) | Mean (SD): 59.9 (11.7) | Mean (SD): 160 (6.12) | Mean (SD): 23.4 (4.28) | <25: 4314 (73.0%) | No: 4498 (76.1%) |
|  | Median [Min, Max]: 28.0 [18.0, 49.0] | Women: 5908 (100%) | Median [Min, Max]: 57.8 [30.6, 159] | Median [Min, Max]: 160 [117, 191] | Median [Min, Max]: 22.5 [11.0, 62.0] | 25+: 1594 (27.0%) | Yes: 1410 (23.9%) |
| **Zimbabwe_2010** | : | : | : | : | : | : | : |
| **(N=11357)** | Mean (SD): 30.5 (9.26) | Men: 5117 (45.1%) | Mean (SD): 62.0 (11.8) | Mean (SD): 165 (8.38) | Mean (SD): 22.9 (4.33) | <25: 8826 (77.7%) | No: 9235 (81.3%) |
|  | Median [Min, Max]: 29.0 [18.0, 54.0] | Women: 6240 (54.9%) | Median [Min, Max]: 60.2 [20.8, 173] | Median [Min, Max]: 165 [106, 199] | Median [Min, Max]: 21.9 [13.2, 62.9] | 25+: 2531 (22.3%) | Yes: 2122 (18.7%) |
| **Zimbabwe_2015** | : | : | : | : | : | : | : |
| **(N=13483)** | Mean (SD): 31.1 (9.20) | Men: 6199 (46.0%) | Mean (SD): 64.7 (12.9) | Mean (SD): 166 (8.54) | Mean (SD): 23.6 (4.69) | <25: 9624 (71.4%) | No: 11168 (82.8%) |
|  | Median [Min, Max]: 30.0 [18.0, 54.0] | Women: 7284 (54.0%) | Median [Min, Max]: 62.5 [25.9, 182] | Median [Min, Max]: 165 [106, 198] | Median [Min, Max]: 22.5 [11.4, 78.0] | 25+: 3859 (28.6%) | Yes: 2315 (17.2%) |
| **Overall** | : | : | : | : | : | : | : |
| **(N=173813)** | Mean (SD): 31.0 (9.49) | Men: 48480 (27.9%) | Mean (SD): 57.7 (11.7) | Mean (SD): 161 (8.43) | Mean (SD): 22.2 (4.27) | <25: 141946 (81.7%) | No: 159858 (92.0%) |
|  | Median [Min, Max]: 30.0 [18.0, 59.0] | Women: 125333 (72.1%) | Median [Min, Max]: 55.8 [15.6, 182] | Median [Min, Max]: 161 [100, 207] | Median [Min, Max]: 21.3 [10.0, 78.0] | 25+: 31867 (18.3%) | Yes: 13955 (8.0%) |

# **Table G in S1 text. Socioeconomic status by country and HIV status.**

| **Country, year** | **Socioeconomic** | **HIV-No** | **HIV-Yes** | **P-value** |
| --- | --- | --- | --- | --- |
| Burundi, 2010 | Poorest | 19.1 | 12.1 | <0.001 |
|  | Poor | 18.7 | 7.7 |  |
|  | Middle | 17.3 | 16.5 |  |
|  | Richer | 17.5 | 5.5 |  |
|  | Richest | 27.5 | 58.2 |  |
| Burundi, 2016 | Poorest | 17.7 | 13.2 | <0.001 |
|  | Poor | 18.5 | 10.5 |  |
|  | Middle | 19.1 | 8.8 |  |
|  | Richer | 19.8 | 11.4 |  |
|  | Richest | 24.9 | 56.1 |  |
| Ethiopia, 2005 | Poorest | 20.8 | 6.2 | <0.001 |
|  | Poor | 16.0 | 5.4 |  |
|  | Middle | 15.5 | 6.9 |  |
|  | Richer | 15.1 | 5.4 |  |
|  | Richest | 32.6 | 76.2 |  |
| Ethiopia, 2011 | Poorest | 21.9 | 6.4 | <0.001 |
|  | Poor | 15.3 | 4.3 |  |
|  | Middle | 14.9 | 6.2 |  |
|  | Richer | 16.1 | 10.4 |  |
|  | Richest | 31.9 | 72.7 |  |
| Ethiopia, 2016 | Poorest | 24.1 | 11.6 | <0.001 |
|  | Poor | 14.2 | 4.5 |  |
|  | Middle | 13.6 | 5.3 |  |
|  | Richer | 14.4 | 8.2 |  |
|  | Richest | 33.7 | 70.4 |  |
| Guinea, 2005 | Poorest | 21.3 | 11.3 | <0.001 |
|  | Poor | 19.4 | 8.1 |  |
|  | Middle | 20.5 | 8.1 |  |
|  | Richer | 21.0 | 40.3 |  |
|  | Richest | 17.8 | 32.3 |  |
| Guinea, 2012 | Poorest | 18.9 | 7.8 | <0.001 |
|  | Poor | 18.9 | 5.6 |  |
|  | Middle | 18.8 | 21.1 |  |
|  | Richer | 23.9 | 26.7 |  |
|  | Richest | 19.5 | 38.9 |  |
| Guinea, 2018 | Poorest | 20.5 | 11.8 | 0.045 |
|  | Poor | 20.7 | 11.8 |  |
|  | Middle | 18.4 | 23.7 |  |
|  | Richer | 19.7 | 27.6 |  |
|  | Richest | 20.7 | 25.0 |  |
| Kenya, 2003 | Poorest | 16.6 | 6.6 | <0.001 |
|  | Poor | 18.0 | 15.8 |  |
|  | Middle | 18.0 | 14.5 |  |
|  | Richer | 19.4 | 23.2 |  |
|  | Richest | 28.1 | 39.8 |  |
| Kenya, 2008 | Poorest | 19.7 | 13.2 | 0.011 |
|  | Poor | 15.2 | 19.9 |  |
|  | Middle | 16.3 | 14.2 |  |
|  | Richer | 19.8 | 18.2 |  |
|  | Richest | 29.0 | 34.5 |  |
| Lesotho, 2004 | Poorest | 18.4 | 12.3 | 0.004 |
|  | Poor | 20.5 | 21.4 |  |
|  | Middle | 18.2 | 17.5 |  |
|  | Richer | 20.1 | 23.0 |  |
|  | Richest | 22.9 | 25.8 |  |
| Lesotho, 2009 | Poorest | 21.6 | 16.2 | <0.001 |
|  | Poor | 20.9 | 20.2 |  |
|  | Middle | 19.1 | 21.4 |  |
|  | Richer | 18.3 | 22.9 |  |
|  | Richest | 20.1 | 19.4 |  |
| Lesotho, 2014 | Poorest | 18.2 | 16.4 | 0.002 |
|  | Poor | 20.1 | 16.6 |  |
|  | Middle | 19.5 | 20.3 |  |
|  | Richer | 19.7 | 23.7 |  |
|  | Richest | 22.5 | 23.0 |  |
| Mali, 2006 | Poorest | 16.5 | 19.6 | 0.112 |
|  | Poor | 19.2 | 12.5 |  |
|  | Middle | 19.7 | 8.9 |  |
|  | Richer | 22.3 | 30.4 |  |
|  | Richest | 22.2 | 28.6 |  |
| Mali, 2012 | Poorest | 18.9 | 14.0 | 0.008 |
|  | Poor | 18.1 | 2.0 |  |
|  | Middle | 17.9 | 20.0 |  |
|  | Richer | 18.9 | 20.0 |  |
|  | Richest | 26.2 | 44.0 |  |
| Malawi, 2004 | Poorest | 18.5 | 15.1 | <0.001 |
|  | Poor | 21.9 | 15.4 |  |
|  | Middle | 22.5 | 21.8 |  |
|  | Richer | 20.6 | 23.5 |  |
|  | Richest | 16.4 | 24.3 |  |
| Malawi, 2010 | Poorest | 18.9 | 13.8 | <0.001 |
|  | Poor | 21.3 | 16.6 |  |
|  | Middle | 20.4 | 17.2 |  |
|  | Richer | 20.9 | 21.7 |  |
|  | Richest | 18.7 | 30.7 |  |
| Malawi, 2015 | Poorest | 17.1 | 15.7 | <0.001 |
|  | Poor | 19.2 | 14.3 |  |
|  | Middle | 18.7 | 17.2 |  |
|  | Richer | 20.1 | 20.1 |  |
|  | Richest | 24.9 | 32.7 |  |
| Niger, 2006 | Poorest | 16.1 | 8.8 | 0.018 |
|  | Poor | 15.8 | 8.8 |  |
|  | Middle | 17.5 | 2.9 |  |
|  | Richer | 18.6 | 26.5 |  |
|  | Richest | 32.1 | 52.9 |  |
| Niger, 2012 | Poorest | 16.4 | 8.3 | 0.052 |
|  | Poor | 16.5 | 8.3 |  |
|  | Middle | 17.5 | 4.2 |  |
|  | Richer | 19.8 | 25.0 |  |
|  | Richest | 29.8 | 54.2 |  |
| Sierra Leone, 2008 | Poorest | 19.0 | 5.3 | 0.031 |
|  | Poor | 17.7 | 21.1 |  |
|  | Middle | 18.2 | 12.3 |  |
|  | Richer | 21.2 | 26.3 |  |
|  | Richest | 23.9 | 35.1 |  |
| Sierra Leone, 2013 | Poorest | 18.9 | 10.4 | <0.001 |
|  | Poor | 17.6 | 10.9 |  |
|  | Middle | 18.3 | 11.5 |  |
|  | Richer | 22.5 | 27.6 |  |
|  | Richest | 22.8 | 39.6 |  |
| Sierra Leone, 2019 | Poorest | 21.5 | 10.1 | <0.001 |
|  | Poor | 19.3 | 13.5 |  |
|  | Middle | 19.7 | 17.4 |  |
|  | Richer | 20.9 | 25.6 |  |
|  | Richest | 18.6 | 33.3 |  |
| Zimbabwe, 2005 | Poorest | 19.5 | 17.2 | <0.001 |
|  | Poor | 18.9 | 19.1 |  |
|  | Middle | 17.9 | 19.5 |  |
|  | Richer | 19.7 | 25.8 |  |
|  | Richest | 24.0 | 18.4 |  |
| Zimbabwe, 2010 | Poorest | 18.9 | 21.0 | <0.001 |
|  | Poor | 17.7 | 17.6 |  |
|  | Middle | 18.4 | 19.8 |  |
|  | Richer | 22.0 | 23.0 |  |
|  | Richest | 23.1 | 18.7 |  |
| Zimbabwe, 2015 | Poorest | 14.3 | 16.6 | <0.001 |
|  | Poor | 15.0 | 14.8 |  |
|  | Middle | 15.9 | 17.1 |  |
|  | Richer | 25.4 | 28.2 |  |
|  | Richest | 29.4 | 23.4 |  |

# **Table H in S1 text. Mean body mass index in the earliest and latest surveys by sex and HIV status; not age-SES standardized (i.e., crude mean estimates).**

| **Period** | **Sex** | **HIV status** | **Sample size** | **Mean BMI** | **Lower 95% confidence interval** | **Upper 95% confidence interval** |
| --- | --- | --- | --- | --- | --- | --- |
| 2011 or before | Women | No | 30395 | 22.35 | 22.25 | 22.44 |
| 2011 or before | Women | Yes | 3168 | 23.04 | 22.84 | 23.23 |
| 2012 or later | Women | No | 50974 | 22.67 | 22.59 | 22.75 |
| 2012 or later | Women | Yes | 4066 | 24.02 | 23.81 | 24.23 |
| 2013 or before | Men | No | 23262 | 20.68 | 20.61 | 20.76 |
| 2013 or before | Men | Yes | 1565 | 21.21 | 21.02 | 21.39 |
| 2014 or later | Men | No | 22113 | 21.07 | 20.98 | 21.17 |
| 2014 or later | Men | Yes | 1540 | 21.46 | 21.26 | 21.66 |

BMI: body mass index (kg/m^2^).

# **Table I in S1 text. Mean body mass index in the earliest and latest surveys by sex and HIV status; age-SES standardized.**

| **Period** | **Sex** | **HIV status** | **Mean BMI** | **Lower 95% confidence interval** | **Upper 95% confidence interval** |
| --- | --- | --- | --- | --- | --- |
| 2011 or before | Women | No | 22.61 | 22.36 | 22.87 |
| 2011 or before | Women | Yes | 22.97 | 22.26 | 23.68 |
| 2012 or later | Women | No | 22.90 | 22.68 | 23.12 |
| 2012 or later | Women | Yes | 23.78 | 23.11 | 24.45 |
| 2013 or before | Men | No | 20.88 | 20.61 | 21.16 |
| 2013 or before | Men | Yes | 21.16 | 20.36 | 21.97 |
| 2014 or later | Men | No | 21.24 | 20.97 | 21.51 |
| 2014 or later | Men | Yes | 21.32 | 20.55 | 22.09 |

BMI: body mass index (kg/m^2^). Underlying results in Figure 1.

# **Table J in S1 text. Prevalence (%) of overweight in the earliest and latest surveys by sex and HIV status; not age-SES standardized (i.e., crude prevalence estimates).**

| **Period** | **Sex** | **HIV status** | **Overweight** | **Sample size** | **Prevalence** | **Lower 95% confidence interval** | **Upper 95% confidence interval** |
| --- | --- | --- | --- | --- | --- | --- | --- |
| 2011 or before | Women | No | <25 | 24234 | 81.07 | 80.23 | 81.88 |
| 2011 or before | Women | No | 25+ | 6161 | 18.93 | 18.12 | 19.77 |
| 2011 or before | Women | Yes | <25 | 2366 | 74.85 | 72.77 | 76.83 |
| 2011 or before | Women | Yes | 25+ | 802 | 25.15 | 23.17 | 27.23 |
| 2012 or later | Women | No | <25 | 39026 | 78.52 | 77.85 | 79.18 |
| 2012 or later | Women | No | 25+ | 11948 | 21.48 | 20.82 | 22.15 |
| 2012 or later | Women | Yes | <25 | 2730 | 66.41 | 64.36 | 68.4 |
| 2012 or later | Women | Yes | 25+ | 1336 | 33.59 | 31.6 | 35.64 |
| 2013 or before | Men | No | <25 | 21619 | 93.54 | 92.98 | 94.05 |
| 2013 or before | Men | No | 25+ | 1643 | 6.46 | 5.95 | 7.02 |
| 2013 or before | Men | Yes | <25 | 1422 | 90.88 | 89.15 | 92.35 |
| 2013 or before | Men | Yes | 25+ | 143 | 9.12 | 7.65 | 10.85 |
| 2014 or later | Men | No | <25 | 19880 | 91.05 | 90.38 | 91.68 |
| 2014 or later | Men | No | 25+ | 2233 | 8.95 | 8.32 | 9.62 |
| 2014 or later | Men | Yes | <25 | 1358 | 88.93 | 86.89 | 90.69 |
| 2014 or later | Men | Yes | 25+ | 182 | 11.07 | 9.31 | 13.11 |

Sample size refers to the absolute number of observations included in the analysis (not weighted population).

# **Table K in S1 text. Prevalence (%) of overweight in the earliest and latest surveys by sex and HIV status; age-SES standardized.**

| **Period** | **Sex** | **HIV status** | **Overweight** | **Prevalence** | **Lower 95% confidence interval** | **Upper 95% confidence interval** |
| --- | --- | --- | --- | --- | --- | --- |
| 2011 or before | Women | No | <25 | 78.34 | 75.99 | 80.52 |
| 2011 or before | Women | No | 25+ | 21.66 | 19.48 | 24.01 |
| 2011 or before | Women | Yes | <25 | 75.10 | 67.61 | 81.22 |
| 2011 or before | Women | Yes | 25+ | 24.90 | 18.78 | 32.39 |
| 2012 or later | Women | No | <25 | 76.16 | 74.22 | 78.00 |
| 2012 or later | Women | No | 25+ | 23.84 | 22.00 | 25.78 |
| 2012 or later | Women | Yes | <25 | 68.58 | 61.37 | 74.90 |
| 2012 or later | Women | Yes | 25+ | 31.42 | 25.10 | 38.63 |
| 2013 or before | Men | No | <25 | 91.59 | 89.05 | 93.53 |
| 2013 or before | Men | No | 25+ | 8.41 | 6.47 | 10.95 |
| 2013 or before | Men | Yes | <25 | 90.90 | 80.56 | 95.52 |
| 2013 or before | Men | Yes | 25+ | 9.10 | 4.48 | 19.44 |
| 2014 or later | Men | No | <25 | 89.22 | 86.65 | 91.27 |
| 2014 or later | Men | No | 25+ | 10.78 | 8.73 | 13.35 |
| 2014 or later | Men | Yes | <25 | 89.66 | 78.83 | 94.75 |
| 2014 or later | Men | Yes | 25+ | 10.85 | 5.51 | 22.22 |

Underlying results in Figure 2.

# **Table L in S1 text. Mean body mass index stratified by HIV status throughout the observation period by sex and country, not age-SES standardized (i.e., crude mean estimates).**

| **Country** | **Year** | **Sex** | **HIV** | **Sample size** | **ART at 50%** | **ART at 75%** | **BMI** | **95% CI, lower** | **95% CI, upper** |
| --- | --- | --- | --- | --- | --- | --- | --- | --- | --- |
| Burundi | 2010 | Women | No | 3253 | 2015 | 2018 | 21.28 | 21.11 | 21.46 |
| Burundi | 2010 | Women | Yes | 91 | 2015 | 2018 | 21.44 | 20.61 | 22.27 |
| Burundi | 2016 | Women | No | 6468 | 2015 | 2018 | 21.06 | 20.95 | 21.17 |
| Burundi | 2016 | Women | Yes | 114 | 2015 | 2018 | 22.25 | 21.22 | 23.27 |
| Ethiopia | 2005 | Women | No | 4501 | 2013 | 2019 | 20.36 | 20.23 | 20.49 |
| Ethiopia | 2005 | Women | Yes | 130 | 2013 | 2019 | 20.35 | 19.38 | 21.33 |
| Ethiopia | 2011 | Men | No | 11167 | 2013 | 2019 | 19.65 | 19.55 | 19.74 |
| Ethiopia | 2011 | Men | Yes | 178 | 2013 | 2019 | 20.94 | 20.21 | 21.66 |
| Ethiopia | 2011 | Women | No | 11752 | 2013 | 2019 | 20.44 | 20.33 | 20.55 |
| Ethiopia | 2011 | Women | Yes | 339 | 2013 | 2019 | 20.40 | 19.83 | 20.98 |
| Ethiopia | 2016 | Men | No | 9812 | 2013 | 2019 | 19.89 | 19.78 | 20.01 |
| Ethiopia | 2016 | Men | Yes | 113 | 2013 | 2019 | 20.67 | 19.67 | 21.67 |
| Ethiopia | 2016 | Women | No | 11252 | 2013 | 2019 | 20.81 | 20.64 | 20.98 |
| Ethiopia | 2016 | Women | Yes | 265 | 2013 | 2019 | 22.68 | 20.41 | 24.94 |
| Malawi | 2004 | Women | No | 1819 | 2014 | 2017 | 22.29 | 22.09 | 22.48 |
| Malawi | 2004 | Women | Yes | 371 | 2014 | 2017 | 22.05 | 21.64 | 22.46 |
| Malawi | 2010 | Women | No | 4801 | 2014 | 2017 | 22.68 | 22.53 | 22.83 |
| Malawi | 2010 | Women | Yes | 796 | 2014 | 2017 | 22.83 | 22.42 | 23.24 |
| Malawi | 2015 | Women | No | 5384 | 2014 | 2017 | 23.04 | 22.90 | 23.17 |
| Malawi | 2015 | Women | Yes | 783 | 2014 | 2017 | 23.30 | 22.91 | 23.69 |
| Kenya | 2003 | Women | No | 2310 | 2014 | 2017 | 23.04 | 22.77 | 23.31 |
| Kenya | 2003 | Women | Yes | 241 | 2014 | 2017 | 22.63 | 21.90 | 23.36 |
| Kenya | 2008 | Women | No | 2796 | 2014 | 2017 | 23.24 | 22.93 | 23.55 |
| Kenya | 2008 | Women | Yes | 281 | 2014 | 2017 | 23.23 | 22.45 | 24.01 |
| Lesotho | 2004 | Women | No | 1636 | 2015 | 2019 | 25.73 | 25.38 | 26.07 |
| Lesotho | 2004 | Women | Yes | 716 | 2015 | 2019 | 24.78 | 24.38 | 25.19 |
| Lesotho | 2009 | Men | No | 2046 | 2015 | 2019 | 21.48 | 21.31 | 21.66 |
| Lesotho | 2009 | Men | Yes | 529 | 2015 | 2019 | 21.44 | 21.09 | 21.78 |
| Lesotho | 2009 | Women | No | 2166 | 2015 | 2019 | 26.14 | 25.82 | 26.47 |
| Lesotho | 2009 | Women | Yes | 935 | 2015 | 2019 | 24.86 | 24.50 | 25.22 |
| Lesotho | 2014 | Men | No | 1847 | 2015 | 2019 | 21.97 | 21.77 | 22.17 |
| Lesotho | 2014 | Men | Yes | 505 | 2015 | 2019 | 21.78 | 21.42 | 22.15 |
| Lesotho | 2014 | Women | No | 1827 | 2015 | 2019 | 26.46 | 26.12 | 26.80 |
| Lesotho | 2014 | Women | Yes | 873 | 2015 | 2019 | 25.43 | 25.03 | 25.83 |
| Zimbabwe | 2005 | Women | No | 4498 | 2013 | 2017 | 23.54 | 23.33 | 23.76 |
| Zimbabwe | 2005 | Women | Yes | 1410 | 2013 | 2017 | 22.78 | 22.52 | 23.05 |
| Zimbabwe | 2010 | Men | No | 4331 | 2013 | 2017 | 21.65 | 21.51 | 21.78 |
| Zimbabwe | 2010 | Men | Yes | 786 | 2013 | 2017 | 21.01 | 20.78 | 21.23 |
| Zimbabwe | 2010 | Women | No | 4904 | 2013 | 2017 | 24.32 | 24.16 | 24.49 |
| Zimbabwe | 2010 | Women | Yes | 1336 | 2013 | 2017 | 23.80 | 23.53 | 24.06 |
| Zimbabwe | 2015 | Men | No | 5344 | 2013 | 2017 | 22.15 | 22.01 | 22.28 |
| Zimbabwe | 2015 | Men | Yes | 855 | 2013 | 2017 | 21.28 | 21.02 | 21.54 |
| Zimbabwe | 2015 | Women | No | 5824 | 2013 | 2017 | 25.01 | 24.84 | 25.19 |
| Zimbabwe | 2015 | Women | Yes | 1460 | 2013 | 2017 | 24.04 | 23.72 | 24.36 |
| Guinea | 2005 | Women | No | 2906 | 2021 | NA | 21.91 | 21.71 | 22.10 |
| Guinea | 2005 | Women | Yes | 62 | 2021 | NA | 22.98 | 21.38 | 24.59 |
| Guinea | 2012 | Women | No | 3496 | 2021 | NA | 22.66 | 22.43 | 22.90 |
| Guinea | 2012 | Women | Yes | 90 | 2021 | NA | 22.64 | 21.53 | 23.75 |
| Guinea | 2018 | Women | No | 3957 | 2021 | NA | 23.72 | 23.49 | 23.94 |
| Guinea | 2018 | Women | Yes | 76 | 2021 | NA | 24.37 | 22.79 | 25.94 |
| Mali | 2006 | Women | No | 3436 | 2020 | NA | 22.44 | 22.11 | 22.77 |
| Mali | 2006 | Women | Yes | 56 | 2020 | NA | 22.74 | 21.34 | 24.13 |
| Mali | 2012 | Women | No | 3966 | 2020 | NA | 22.51 | 22.31 | 22.71 |
| Mali | 2012 | Women | Yes | 50 | 2020 | NA | 23.65 | 22.17 | 25.13 |
| Niger | 2006 | Women | No | 3263 | 2017 | 2021 | 21.61 | 21.39 | 21.83 |
| Niger | 2006 | Women | Yes | 34 | 2017 | 2021 | 22.79 | 21.56 | 24.02 |
| Niger | 2012 | Women | No | 3845 | 2017 | 2021 | 22.29 | 22.06 | 22.51 |
| Niger | 2012 | Women | Yes | 24 | 2017 | 2021 | 23.17 | 20.90 | 25.44 |
| Sierra Leone | 2008 | Women | No | 2773 | 2020 | NA | 23.69 | 23.29 | 24.10 |
| Sierra Leone | 2008 | Women | Yes | 57 | 2020 | NA | 22.62 | 21.15 | 24.09 |
| Sierra Leone | 2013 | Men | No | 5718 | 2020 | NA | 21.69 | 21.54 | 21.84 |
| Sierra Leone | 2013 | Men | Yes | 72 | 2020 | NA | 21.98 | 21.34 | 22.61 |
| Sierra Leone | 2013 | Women | No | 5995 | 2020 | NA | 22.80 | 22.60 | 23.01 |
| Sierra Leone | 2013 | Women | Yes | 120 | 2020 | NA | 23.37 | 22.56 | 24.19 |
| Sierra Leone | 2019 | Men | No | 5110 | 2020 | NA | 21.97 | 21.85 | 22.09 |
| Sierra Leone | 2019 | Men | Yes | 67 | 2020 | NA | 21.84 | 21.22 | 22.45 |
| Sierra Leone | 2019 | Women | No | 5655 | 2020 | NA | 23.82 | 23.64 | 24.00 |
| Sierra Leone | 2019 | Women | Yes | 140 | 2020 | NA | 23.07 | 22.30 | 23.83 |

BMI: body mass index (kg/m^2^).

# **Table M in S1 text. Age-SES standardized mean body mass index stratified by HIV status throughout the observation period by sex and country.**

|  | **Year** | **Sex** | **HIV** | **Age-SES STANDARDIZED** | | |
| --- | --- | --- | --- | --- | --- | --- |
|  |  |  |  | **BMI** | **95% CI, lower** | **95% CI, upper** |
| Burundi | 2010 | Women | No | 21.29 | 20.74 | 21.83 |
| Burundi | 2010 | Women | Yes | 21.00 | 19.33 | 22.67 |
| Burundi | 2016 | Women | No | 21.11 | 20.77 | 21.45 |
| Burundi | 2016 | Women | Yes | 21.08 | 18.68 | 23.48 |
| Ethiopia | 2005 | Women | No | 20.33 | 19.87 | 20.79 |
| Ethiopia | 2005 | Women | Yes | 19.86 | 18.58 | 21.14 |
| Ethiopia | 2011 | Men | No | 19.82 | 19.49 | 20.14 |
| Ethiopia | 2011 | Men | Yes | 20.14 | 18.56 | 21.73 |
| Ethiopia | 2011 | Women | No | 20.50 | 20.18 | 20.81 |
| Ethiopia | 2011 | Women | Yes | 19.76 | 18.30 | 21.22 |
| Ethiopia | 2016 | Men | No | 20.03 | 19.71 | 20.36 |
| Ethiopia | 2016 | Men | Yes | 20.11 | 18.93 | 21.29 |
| Ethiopia | 2016 | Women | No | 20.81 | 20.46 | 21.17 |
| Ethiopia | 2016 | Women | Yes | 20.98 | 19.39 | 22.56 |
| Malawi | 2004 | Women | No | 22.65 | 21.86 | 23.44 |
| Malawi | 2004 | Women | Yes | 21.92 | 20.50 | 23.33 |
| Malawi | 2010 | Women | No | 22.87 | 22.32 | 23.43 |
| Malawi | 2010 | Women | Yes | 22.45 | 21.37 | 23.53 |
| Malawi | 2015 | Women | No | 23.32 | 22.77 | 23.87 |
| Malawi | 2015 | Women | Yes | 23.03 | 21.78 | 24.27 |
| Kenya | 2003 | Women | No | 23.45 | 22.68 | 24.22 |
| Kenya | 2003 | Women | Yes | 22.45 | 19.92 | 24.98 |
| Kenya | 2008 | Women | No | 23.49 | 22.53 | 24.45 |
| Kenya | 2008 | Women | Yes | 23.27 | 21.14 | 25.40 |
| Lesotho | 2004 | Women | No | 26.26 | 25.00 | 27.51 |
| Lesotho | 2004 | Women | Yes | 24.81 | 23.11 | 26.50 |
| Lesotho | 2009 | Men | No | 22.13 | 21.16 | 23.10 |
| Lesotho | 2009 | Men | Yes | 21.39 | 20.01 | 22.76 |
| Lesotho | 2009 | Women | No | 26.64 | 25.54 | 27.74 |
| Lesotho | 2009 | Women | Yes | 24.78 | 23.37 | 26.18 |
| Lesotho | 2014 | Men | No | 22.61 | 21.62 | 23.60 |
| Lesotho | 2014 | Men | Yes | 21.68 | 20.28 | 23.08 |
| Lesotho | 2014 | Women | No | 27.30 | 26.12 | 28.48 |
| Lesotho | 2014 | Women | Yes | 25.27 | 23.76 | 26.78 |
| Zimbabwe | 2005 | Women | No | 24.14 | 23.54 | 24.74 |
| Zimbabwe | 2005 | Women | Yes | 22.86 | 21.95 | 23.77 |
| Zimbabwe | 2010 | Men | No | 22.01 | 21.26 | 22.76 |
| Zimbabwe | 2010 | Men | Yes | 21.05 | 19.96 | 22.14 |
| Zimbabwe | 2010 | Women | No | 24.99 | 24.35 | 25.63 |
| Zimbabwe | 2010 | Women | Yes | 23.88 | 22.88 | 24.89 |
| Zimbabwe | 2015 | Men | No | 22.38 | 21.86 | 22.90 |
| Zimbabwe | 2015 | Men | Yes | 21.21 | 20.23 | 22.19 |
| Zimbabwe | 2015 | Women | No | 25.48 | 24.90 | 26.06 |
| Zimbabwe | 2015 | Women | Yes | 23.96 | 22.99 | 24.93 |
| Guinea | 2005 | Women | No | 22.13 | 21.55 | 22.71 |
| Guinea | 2005 | Women | Yes | 22.16 | 20.43 | 23.89 |
| Guinea | 2012 | Women | No | 22.96 | 22.31 | 23.60 |
| Guinea | 2012 | Women | Yes | 21.60 | 19.30 | 23.91 |
| Guinea | 2018 | Women | No | 24.17 | 23.48 | 24.85 |
| Guinea | 2018 | Women | Yes | 23.44 | 20.91 | 25.98 |
| Mali | 2006 | Women | No | 22.72 | 22.00 | 23.44 |
| Mali | 2006 | Women | Yes | 22.52 | 19.92 | 25.11 |
| Mali | 2012 | Women | No | 22.79 | 22.21 | 23.37 |
| Mali | 2012 | Women | Yes | 23.21 | 20.26 | 26.15 |
| Niger | 2006 | Women | No | 21.82 | 21.27 | 22.38 |
| Niger | 2006 | Women | Yes | 22.55 | 20.87 | 24.24 |
| Niger | 2012 | Women | No | 22.43 | 21.83 | 23.05 |
| Niger | 2012 | Women | Yes | 22.50 | 20.73 | 24.27 |
| Sierra Leone | 2008 | Women | No | 23.97 | 22.94 | 24.99 |
| Sierra Leone | 2008 | Women | Yes | 22.15 | 19.92 | 24.37 |
| Sierra Leone | 2013 | Men | No | 21.82 | 21.34 | 22.30 |
| Sierra Leone | 2013 | Men | Yes | 21.63 | 20.01 | 23.24 |
| Sierra Leone | 2013 | Women | No | 23.06 | 22.53 | 23.60 |
| Sierra Leone | 2013 | Women | Yes | 22.77 | 20.97 | 24.58 |
| Sierra Leone | 2019 | Men | No | 22.10 | 21.66 | 22.53 |
| Sierra Leone | 2019 | Men | Yes | 21.54 | 20.10 | 22.99 |
| Sierra Leone | 2019 | Women | No | 24.19 | 23.60 | 24.78 |
| Sierra Leone | 2019 | Women | Yes | 23.00 | 20.34 | 25.66 |

BMI: body mass index (kg/m^2^).

# **Table N in S1 text. Prevalence of overweight stratified by HIV status throughout the observation period by sex, not age-SES standardized (i.e., crude prevalence estimates).**

| **Country** | **Year** | **Sex** | **HIV** | **Overweight** | **Sample size** | **Prevalence** | **95% CI, lower** | **95% CI, upper** |
| --- | --- | --- | --- | --- | --- | --- | --- | --- |
| Burundi | 2010 | Women | No | <25 | 2906 | 91.71 | 90.60 | 92.71 |
| Burundi | 2010 | Women | No | 25+ | 347 | 8.29 | 7.29 | 9.40 |
| Burundi | 2010 | Women | Yes | <25 | 73 | 86.57 | 77.62 | 92.30 |
| Burundi | 2010 | Women | Yes | 25+ | 18 | 13.43 | 7.70 | 22.38 |
| Burundi | 2016 | Women | No | <25 | 5840 | 91.60 | 90.66 | 92.46 |
| Burundi | 2016 | Women | No | 25+ | 628 | 8.40 | 7.54 | 9.34 |
| Burundi | 2016 | Women | Yes | <25 | 88 | 81.81 | 73.25 | 88.07 |
| Burundi | 2016 | Women | Yes | 25+ | 26 | 18.19 | 11.93 | 26.75 |
| Ethiopia | 2005 | Women | No | <25 | 4214 | 95.59 | 94.62 | 96.38 |
| Ethiopia | 2005 | Women | No | 25+ | 287 | 4.41 | 3.62 | 5.38 |
| Ethiopia | 2005 | Women | Yes | <25 | 120 | 92.70 | 77.78 | 97.88 |
| Ethiopia | 2005 | Women | Yes | 25+ | 10 | 7.30 | 2.12 | 22.22 |
| Ethiopia | 2011 | Men | No | <25 | 10735 | 97.31 | 96.71 | 97.81 |
| Ethiopia | 2011 | Men | No | 25+ | 432 | 2.69 | 2.19 | 3.29 |
| Ethiopia | 2011 | Men | Yes | <25 | 159 | 92.05 | 84.94 | 95.96 |
| Ethiopia | 2011 | Men | Yes | 25+ | 19 | 7.95 | 4.04 | 15.06 |
| Ethiopia | 2011 | Women | No | <25 | 10779 | 93.86 | 92.99 | 94.63 |
| Ethiopia | 2011 | Women | No | 25+ | 973 | 6.14 | 5.37 | 7.01 |
| Ethiopia | 2011 | Women | Yes | <25 | 293 | 90.41 | 85.24 | 93.89 |
| Ethiopia | 2011 | Women | Yes | 25+ | 46 | 9.59 | 6.11 | 14.76 |
| Ethiopia | 2016 | Men | No | <25 | 9249 | 96.36 | 95.57 | 97.01 |
| Ethiopia | 2016 | Men | No | 25+ | 563 | 3.64 | 2.99 | 4.43 |
| Ethiopia | 2016 | Men | Yes | <25 | 105 | 94.24 | 78.27 | 98.67 |
| Ethiopia | 2016 | Men | Yes | 25+ | 8 | 5.76 | 1.33 | 21.73 |
| Ethiopia | 2016 | Women | No | <25 | 9881 | 92.16 | 90.76 | 93.37 |
| Ethiopia | 2016 | Women | No | 25+ | 1371 | 7.84 | 6.63 | 9.24 |
| Ethiopia | 2016 | Women | Yes | <25 | 199 | 76.90 | 62.62 | 86.87 |
| Ethiopia | 2016 | Women | Yes | 25+ | 66 | 23.10 | 13.13 | 37.38 |
| Malawi | 2004 | Women | No | <25 | 1564 | 85.55 | 83.43 | 87.44 |
| Malawi | 2004 | Women | No | 25+ | 255 | 14.45 | 12.56 | 16.57 |
| Malawi | 2004 | Women | Yes | <25 | 318 | 85.47 | 80.78 | 89.17 |
| Malawi | 2004 | Women | Yes | 25+ | 53 | 14.53 | 10.83 | 19.22 |
| Malawi | 2010 | Women | No | <25 | 3981 | 81.64 | 80.08 | 83.10 |
| Malawi | 2010 | Women | No | 25+ | 820 | 18.36 | 16.90 | 19.92 |
| Malawi | 2010 | Women | Yes | <25 | 642 | 79.06 | 74.94 | 82.66 |
| Malawi | 2010 | Women | Yes | 25+ | 154 | 20.94 | 17.34 | 25.06 |
| Malawi | 2015 | Women | No | <25 | 4101 | 77.65 | 76.18 | 79.06 |
| Malawi | 2015 | Women | No | 25+ | 1283 | 22.35 | 20.94 | 23.82 |
| Malawi | 2015 | Women | Yes | <25 | 575 | 71.81 | 67.72 | 75.57 |
| Malawi | 2015 | Women | Yes | 25+ | 208 | 28.19 | 24.43 | 32.28 |
| Kenya | 2003 | Women | No | <25 | 1677 | 73.53 | 70.96 | 75.95 |
| Kenya | 2003 | Women | No | 25+ | 633 | 26.47 | 24.05 | 29.04 |
| Kenya | 2003 | Women | Yes | <25 | 187 | 78.11 | 70.34 | 84.30 |
| Kenya | 2003 | Women | Yes | 25+ | 54 | 21.89 | 15.70 | 29.66 |
| Kenya | 2008 | Women | No | <25 | 1997 | 72.42 | 69.34 | 75.30 |
| Kenya | 2008 | Women | No | 25+ | 799 | 27.58 | 24.70 | 30.66 |
| Kenya | 2008 | Women | Yes | <25 | 214 | 70.22 | 59.42 | 79.15 |
| Kenya | 2008 | Women | Yes | 25+ | 67 | 29.78 | 20.85 | 40.58 |
| Lesotho | 2004 | Women | No | <25 | 916 | 54.24 | 51.10 | 57.35 |
| Lesotho | 2004 | Women | No | 25+ | 720 | 45.76 | 42.65 | 48.90 |
| Lesotho | 2004 | Women | Yes | <25 | 415 | 58.43 | 54.47 | 62.28 |
| Lesotho | 2004 | Women | Yes | 25+ | 301 | 41.57 | 37.72 | 45.53 |
| Lesotho | 2009 | Men | No | <25 | 1813 | 87.66 | 85.85 | 89.27 |
| Lesotho | 2009 | Men | No | 25+ | 233 | 12.34 | 10.73 | 14.15 |
| Lesotho | 2009 | Men | Yes | <25 | 478 | 89.20 | 86.07 | 91.69 |
| Lesotho | 2009 | Men | Yes | 25+ | 51 | 10.80 | 8.31 | 13.93 |
| Lesotho | 2009 | Women | No | <25 | 1173 | 50.90 | 48.17 | 53.62 |
| Lesotho | 2009 | Women | No | 25+ | 993 | 49.10 | 46.38 | 51.83 |
| Lesotho | 2009 | Women | Yes | <25 | 562 | 60.02 | 56.16 | 63.75 |
| Lesotho | 2009 | Women | Yes | 25+ | 373 | 39.98 | 36.25 | 43.84 |
| Lesotho | 2014 | Men | No | <25 | 1569 | 84.75 | 82.68 | 86.61 |
| Lesotho | 2014 | Men | No | 25+ | 278 | 15.25 | 13.39 | 17.32 |
| Lesotho | 2014 | Men | Yes | <25 | 437 | 87.64 | 83.76 | 90.70 |
| Lesotho | 2014 | Men | Yes | 25+ | 68 | 12.36 | 9.30 | 16.24 |
| Lesotho | 2014 | Women | No | <25 | 912 | 49.08 | 46.41 | 51.76 |
| Lesotho | 2014 | Women | No | 25+ | 915 | 50.92 | 48.24 | 53.59 |
| Lesotho | 2014 | Women | Yes | <25 | 479 | 54.60 | 50.68 | 58.47 |
| Lesotho | 2014 | Women | Yes | 25+ | 394 | 45.40 | 41.53 | 49.32 |
| Zimbabwe | 2005 | Women | No | <25 | 3219 | 71.82 | 69.56 | 73.98 |
| Zimbabwe | 2005 | Women | No | 25+ | 1279 | 28.18 | 26.02 | 30.44 |
| Zimbabwe | 2005 | Women | Yes | <25 | 1095 | 78.00 | 74.79 | 80.92 |
| Zimbabwe | 2005 | Women | Yes | 25+ | 315 | 22.00 | 19.08 | 25.21 |
| Zimbabwe | 2010 | Men | No | <25 | 3910 | 89.64 | 88.34 | 90.81 |
| Zimbabwe | 2010 | Men | No | 25+ | 421 | 10.36 | 9.19 | 11.66 |
| Zimbabwe | 2010 | Men | Yes | <25 | 723 | 92.42 | 90.10 | 94.23 |
| Zimbabwe | 2010 | Men | Yes | 25+ | 63 | 7.58 | 5.77 | 9.90 |
| Zimbabwe | 2010 | Women | No | <25 | 3253 | 64.85 | 63.27 | 66.39 |
| Zimbabwe | 2010 | Women | No | 25+ | 1651 | 35.15 | 33.61 | 36.73 |
| Zimbabwe | 2010 | Women | Yes | <25 | 940 | 69.48 | 66.77 | 72.07 |
| Zimbabwe | 2010 | Women | Yes | 25+ | 396 | 30.52 | 27.93 | 33.23 |
| Zimbabwe | 2015 | Men | No | <25 | 4514 | 85.56 | 84.09 | 86.92 |
| Zimbabwe | 2015 | Men | No | 25+ | 830 | 14.44 | 13.08 | 15.91 |
| Zimbabwe | 2015 | Men | Yes | <25 | 758 | 89.26 | 86.56 | 91.47 |
| Zimbabwe | 2015 | Men | Yes | 25+ | 97 | 10.74 | 8.53 | 13.44 |
| Zimbabwe | 2015 | Women | No | <25 | 3381 | 59.80 | 58.13 | 61.46 |
| Zimbabwe | 2015 | Women | No | 25+ | 2443 | 40.20 | 38.54 | 41.87 |
| Zimbabwe | 2015 | Women | Yes | <25 | 971 | 67.20 | 63.56 | 70.65 |
| Zimbabwe | 2015 | Women | Yes | 25+ | 489 | 32.80 | 29.35 | 36.44 |
| Guinea | 2005 | Women | No | <25 | 2485 | 84.81 | 82.81 | 86.62 |
| Guinea | 2005 | Women | No | 25+ | 421 | 15.19 | 13.38 | 17.19 |
| Guinea | 2005 | Women | Yes | <25 | 49 | 71.99 | 56.84 | 83.38 |
| Guinea | 2005 | Women | Yes | 25+ | 13 | 28.01 | 16.62 | 43.16 |
| Guinea | 2012 | Women | No | <25 | 2783 | 78.90 | 76.71 | 80.93 |
| Guinea | 2012 | Women | No | 25+ | 713 | 21.10 | 19.07 | 23.29 |
| Guinea | 2012 | Women | Yes | <25 | 71 | 80.79 | 69.96 | 88.37 |
| Guinea | 2012 | Women | Yes | 25+ | 19 | 19.21 | 11.63 | 30.04 |
| Guinea | 2018 | Women | No | <25 | 2814 | 71.05 | 68.81 | 73.20 |
| Guinea | 2018 | Women | No | 25+ | 1143 | 28.95 | 26.80 | 31.19 |
| Guinea | 2018 | Women | Yes | <25 | 51 | 64.52 | 52.18 | 75.19 |
| Guinea | 2018 | Women | Yes | 25+ | 25 | 35.48 | 24.81 | 47.82 |
| Mali | 2006 | Women | No | <25 | 2726 | 79.84 | 76.41 | 82.89 |
| Mali | 2006 | Women | No | 25+ | 710 | 20.16 | 17.11 | 23.59 |
| Mali | 2006 | Women | Yes | <25 | 46 | 80.36 | 65.28 | 89.90 |
| Mali | 2006 | Women | Yes | 25+ | 10 | 19.64 | 10.10 | 34.72 |
| Mali | 2012 | Women | No | <25 | 3137 | 81.17 | 79.35 | 82.87 |
| Mali | 2012 | Women | No | 25+ | 829 | 18.83 | 17.13 | 20.65 |
| Mali | 2012 | Women | Yes | <25 | 37 | 72.43 | 56.53 | 84.15 |
| Mali | 2012 | Women | Yes | 25+ | 13 | 27.57 | 15.85 | 43.47 |
| Niger | 2006 | Women | No | <25 | 2630 | 86.11 | 84.15 | 87.87 |
| Niger | 2006 | Women | No | 25+ | 633 | 13.89 | 12.13 | 15.85 |
| Niger | 2006 | Women | Yes | <25 | 23 | 77.50 | 58.61 | 89.34 |
| Niger | 2006 | Women | Yes | 25+ | 11 | 22.50 | 10.66 | 41.39 |
| Niger | 2012 | Women | No | <25 | 2970 | 80.66 | 78.60 | 82.56 |
| Niger | 2012 | Women | No | 25+ | 875 | 19.34 | 17.44 | 21.40 |
| Niger | 2012 | Women | Yes | <25 | 17 | 71.98 | 48.66 | 87.44 |
| Niger | 2012 | Women | Yes | 25+ | 7 | 28.02 | 12.56 | 51.34 |
| Sierra Leone | 2008 | Women | No | <25 | 1897 | 70.06 | 66.36 | 73.52 |
| Sierra Leone | 2008 | Women | No | 25+ | 876 | 29.94 | 26.48 | 33.64 |
| Sierra Leone | 2008 | Women | Yes | <25 | 40 | 72.12 | 57.95 | 82.93 |
| Sierra Leone | 2008 | Women | Yes | 25+ | 17 | 27.88 | 17.07 | 42.05 |
| Sierra Leone | 2013 | Men | No | <25 | 5161 | 91.19 | 89.61 | 92.54 |
| Sierra Leone | 2013 | Men | No | 25+ | 557 | 8.81 | 7.46 | 10.39 |
| Sierra Leone | 2013 | Men | Yes | <25 | 62 | 85.98 | 74.65 | 92.74 |
| Sierra Leone | 2013 | Men | Yes | 25+ | 10 | 14.02 | 7.26 | 25.35 |
| Sierra Leone | 2013 | Women | No | <25 | 4742 | 79.85 | 77.88 | 81.68 |
| Sierra Leone | 2013 | Women | No | 25+ | 1253 | 20.15 | 18.32 | 22.12 |
| Sierra Leone | 2013 | Women | Yes | <25 | 92 | 74.52 | 62.89 | 83.46 |
| Sierra Leone | 2013 | Women | Yes | 25+ | 28 | 25.48 | 16.54 | 37.11 |
| Sierra Leone | 2019 | Men | No | <25 | 4548 | 88.52 | 87.24 | 89.69 |
| Sierra Leone | 2019 | Men | No | 25+ | 562 | 11.48 | 10.31 | 12.76 |
| Sierra Leone | 2019 | Men | Yes | <25 | 58 | 89.55 | 79.13 | 95.09 |
| Sierra Leone | 2019 | Men | Yes | 25+ | 9 | 10.45 | 4.91 | 20.87 |
| Sierra Leone | 2019 | Women | No | <25 | 3993 | 69.43 | 67.70 | 71.10 |
| Sierra Leone | 2019 | Women | No | 25+ | 1662 | 30.57 | 28.90 | 32.30 |
| Sierra Leone | 2019 | Women | Yes | <25 | 99 | 72.02 | 62.39 | 79.98 |
| Sierra Leone | 2019 | Women | Yes | 25+ | 41 | 27.98 | 20.02 | 37.61 |

Overweight is the categorization of body mass index (kg/m^2^).

# **Table O in S1 text. Age-SES standardized prevalence of overweight stratified by HIV status throughout the observation period by sex.**

| **Country** | **Year** | **Sex** | **HIV** | **Overweight** | **Age-SES STANDARDIZED** | | |
| --- | --- | --- | --- | --- | --- | --- | --- |
|  |  |  |  |  | **Prevalence** | **95% CI, lower** | **95% CI, upper** |
| Burundi | 2010 | Women | No | <25 | 90.77 | 85.42 | 93.88 |
| Burundi | 2010 | Women | No | 25+ | 9.23 | 6.12 | 14.58 |
| Burundi | 2010 | Women | Yes | <25 | 92.43 | 83.18 | 97.29 |
| Burundi | 2010 | Women | Yes | 25+ | 24.46 | 8.75 | 54.36 |
| Burundi | 2016 | Women | No | <25 | 90.32 | 86.74 | 92.81 |
| Burundi | 2016 | Women | No | 25+ | 9.68 | 7.19 | 13.26 |
| Burundi | 2016 | Women | Yes | <25 | 88.71 | 73.59 | 95.96 |
| Burundi | 2016 | Women | Yes | 25+ | 26.59 | 9.52 | 62.18 |
| Ethiopia | 2005 | Women | No | <25 | 95.18 | 90.73 | 97.27 |
| Ethiopia | 2005 | Women | No | 25+ | 5.17 | 2.93 | 9.96 |
| Ethiopia | 2005 | Women | Yes | <25 | 93.60 | 84.91 | 99.15 |
| Ethiopia | 2005 | Women | Yes | 25+ | 19.35 | 2.57 | 45.64 |
| Ethiopia | 2011 | Men | No | <25 | 96.30 | 93.25 | 97.73 |
| Ethiopia | 2011 | Men | No | 25+ | 4.03 | 2.48 | 7.36 |
| Ethiopia | 2011 | Men | Yes | <25 | 95.08 | 88.44 | 98.81 |
| Ethiopia | 2011 | Men | Yes | 25+ | 14.22 | 3.45 | 33.42 |
| Ethiopia | 2011 | Women | No | <25 | 92.79 | 89.78 | 94.72 |
| Ethiopia | 2011 | Women | No | 25+ | 7.21 | 5.28 | 10.22 |
| Ethiopia | 2011 | Women | Yes | <25 | 95.93 | 89.36 | 98.43 |
| Ethiopia | 2011 | Women | Yes | 25+ | 11.18 | 4.32 | 29.23 |
| Ethiopia | 2016 | Men | No | <25 | 95.41 | 92.17 | 96.90 |
| Ethiopia | 2016 | Men | No | 25+ | 4.59 | 3.10 | 7.83 |
| Ethiopia | 2016 | Men | Yes | <25 | 96.74 | 91.15 | 99.51 |
| Ethiopia | 2016 | Men | Yes | 25+ | 17.36 | 2.63 | 47.11 |
| Ethiopia | 2016 | Women | No | <25 | 91.71 | 88.47 | 93.94 |
| Ethiopia | 2016 | Women | No | 25+ | 8.29 | 6.06 | 11.53 |
| Ethiopia | 2016 | Women | Yes | <25 | 92.24 | 80.43 | 97.00 |
| Ethiopia | 2016 | Women | Yes | 25+ | 15.95 | 6.16 | 40.21 |
| Malawi | 2004 | Women | No | <25 | 81.58 | 71.50 | 88.64 |
| Malawi | 2004 | Women | No | 25+ | 18.42 | 11.36 | 28.50 |
| Malawi | 2004 | Women | Yes | <25 | 85.79 | 65.87 | 94.70 |
| Malawi | 2004 | Women | Yes | 25+ | 16.45 | 6.14 | 39.52 |
| Malawi | 2010 | Women | No | <25 | 79.02 | 72.38 | 84.37 |
| Malawi | 2010 | Women | No | 25+ | 20.98 | 15.63 | 27.62 |
| Malawi | 2010 | Women | Yes | <25 | 82.81 | 67.18 | 90.90 |
| Malawi | 2010 | Women | Yes | 25+ | 17.19 | 9.10 | 32.82 |
| Malawi | 2015 | Women | No | <25 | 74.55 | 68.27 | 79.84 |
| Malawi | 2015 | Women | No | 25+ | 25.45 | 20.16 | 31.73 |
| Malawi | 2015 | Women | Yes | <25 | 74.84 | 57.55 | 85.89 |
| Malawi | 2015 | Women | Yes | 25+ | 25.16 | 14.11 | 42.45 |
| Kenya | 2003 | Women | No | <25 | 69.58 | 60.87 | 76.99 |
| Kenya | 2003 | Women | No | 25+ | 30.42 | 23.01 | 39.13 |
| Kenya | 2003 | Women | Yes | <25 | 78.57 | 47.31 | 92.40 |
| Kenya | 2003 | Women | Yes | 25+ | 21.43 | 7.60 | 52.69 |
| Kenya | 2008 | Women | No | <25 | 70.01 | 60.42 | 78.26 |
| Kenya | 2008 | Women | No | 25+ | 29.99 | 21.74 | 39.58 |
| Kenya | 2008 | Women | Yes | <25 | 71.53 | 47.52 | 88.72 |
| Kenya | 2008 | Women | Yes | 25+ | 32.40 | 12.84 | 59.72 |
| Lesotho | 2004 | Women | No | <25 | 49.47 | 38.87 | 60.48 |
| Lesotho | 2004 | Women | No | 25+ | 50.53 | 39.52 | 61.13 |
| Lesotho | 2004 | Women | Yes | <25 | 58.91 | 42.03 | 73.78 |
| Lesotho | 2004 | Women | Yes | 25+ | 41.09 | 26.22 | 57.97 |
| Lesotho | 2009 | Men | No | <25 | 80.30 | 68.73 | 88.46 |
| Lesotho | 2009 | Men | No | 25+ | 19.70 | 11.54 | 31.27 |
| Lesotho | 2009 | Men | Yes | <25 | 89.13 | 70.05 | 96.32 |
| Lesotho | 2009 | Men | Yes | 25+ | 13.09 | 4.44 | 36.05 |
| Lesotho | 2009 | Women | No | <25 | 46.99 | 37.73 | 56.70 |
| Lesotho | 2009 | Women | No | 25+ | 53.01 | 43.30 | 62.27 |
| Lesotho | 2009 | Women | Yes | <25 | 61.66 | 47.47 | 73.75 |
| Lesotho | 2009 | Women | Yes | 25+ | 38.34 | 26.25 | 52.53 |
| Lesotho | 2014 | Men | No | <25 | 77.58 | 65.01 | 86.41 |
| Lesotho | 2014 | Men | No | 25+ | 22.42 | 13.59 | 34.99 |
| Lesotho | 2014 | Men | Yes | <25 | 87.87 | 67.86 | 95.70 |
| Lesotho | 2014 | Men | Yes | 25+ | 13.55 | 4.80 | 35.90 |
| Lesotho | 2014 | Women | No | <25 | 42.36 | 33.24 | 52.74 |
| Lesotho | 2014 | Women | No | 25+ | 57.64 | 47.26 | 66.76 |
| Lesotho | 2014 | Women | Yes | <25 | 55.70 | 41.20 | 69.17 |
| Lesotho | 2014 | Women | Yes | 25+ | 44.30 | 30.83 | 58.80 |
| Zimbabwe | 2005 | Women | No | <25 | 66.20 | 60.00 | 71.88 |
| Zimbabwe | 2005 | Women | No | 25+ | 33.80 | 28.12 | 40.00 |
| Zimbabwe | 2005 | Women | Yes | <25 | 77.00 | 66.26 | 84.95 |
| Zimbabwe | 2005 | Women | Yes | 25+ | 23.00 | 15.05 | 33.74 |
| Zimbabwe | 2010 | Men | No | <25 | 85.72 | 77.84 | 90.76 |
| Zimbabwe | 2010 | Men | No | 25+ | 14.28 | 9.24 | 22.16 |
| Zimbabwe | 2010 | Men | Yes | <25 | 91.37 | 77.26 | 96.77 |
| Zimbabwe | 2010 | Men | Yes | 25+ | 9.54 | 3.57 | 25.13 |
| Zimbabwe | 2010 | Women | No | <25 | 59.13 | 53.02 | 65.02 |
| Zimbabwe | 2010 | Women | No | 25+ | 40.87 | 34.98 | 46.98 |
| Zimbabwe | 2010 | Women | Yes | <25 | 69.03 | 57.79 | 78.23 |
| Zimbabwe | 2010 | Women | Yes | 25+ | 30.97 | 21.77 | 42.21 |
| Zimbabwe | 2015 | Men | No | <25 | 82.39 | 75.83 | 86.98 |
| Zimbabwe | 2015 | Men | No | 25+ | 18.39 | 13.60 | 25.24 |
| Zimbabwe | 2015 | Men | Yes | <25 | 89.42 | 78.15 | 94.92 |
| Zimbabwe | 2015 | Men | Yes | 25+ | 13.62 | 6.54 | 28.12 |
| Zimbabwe | 2015 | Women | No | <25 | 55.33 | 49.50 | 61.07 |
| Zimbabwe | 2015 | Women | No | 25+ | 44.67 | 38.93 | 50.50 |
| Zimbabwe | 2015 | Women | Yes | <25 | 68.04 | 56.17 | 77.62 |
| Zimbabwe | 2015 | Women | Yes | 25+ | 31.96 | 22.38 | 43.83 |
| Guinea | 2005 | Women | No | <25 | 82.41 | 75.79 | 87.41 |
| Guinea | 2005 | Women | No | 25+ | 17.59 | 12.59 | 24.21 |
| Guinea | 2005 | Women | Yes | <25 | 80.27 | 53.46 | 93.42 |
| Guinea | 2005 | Women | Yes | 25+ | 37.18 | 19.18 | 73.83 |
| Guinea | 2012 | Women | No | <25 | 76.10 | 69.47 | 81.52 |
| Guinea | 2012 | Women | No | 25+ | 23.90 | 18.48 | 30.53 |
| Guinea | 2012 | Women | Yes | <25 | 88.95 | 69.75 | 97.16 |
| Guinea | 2012 | Women | Yes | 25+ | 22.29 | 5.73 | 61.05 |
| Guinea | 2018 | Women | No | <25 | 66.73 | 60.24 | 72.66 |
| Guinea | 2018 | Women | No | 25+ | 33.27 | 27.34 | 39.76 |
| Guinea | 2018 | Women | Yes | <25 | 73.98 | 50.08 | 91.16 |
| Guinea | 2018 | Women | Yes | 25+ | 38.35 | 13.02 | 73.56 |
| Mali | 2006 | Women | No | <25 | 77.09 | 69.12 | 83.58 |
| Mali | 2006 | Women | No | 25+ | 22.91 | 16.42 | 30.88 |
| Mali | 2006 | Women | Yes | <25 | 85.14 | 66.38 | 97.32 |
| Mali | 2006 | Women | Yes | 25+ | 30.44 | 5.50 | 68.86 |
| Mali | 2012 | Women | No | <25 | 78.49 | 72.45 | 83.41 |
| Mali | 2012 | Women | No | 25+ | 21.51 | 16.59 | 27.55 |
| Mali | 2012 | Women | Yes | <25 | 78.73 | 52.32 | 94.55 |
| Mali | 2012 | Women | Yes | 25+ | 34.69 | 8.89 | 77.76 |
| Niger | 2006 | Women | No | <25 | 83.96 | 78.18 | 88.10 |
| Niger | 2006 | Women | No | 25+ | 16.04 | 11.90 | 21.82 |
| Niger | 2006 | Women | Yes | <25 | 80.50 | 60.34 | 95.62 |
| Niger | 2006 | Women | Yes | 25+ | 39.69 | 8.92 | 80.73 |
| Niger | 2012 | Women | No | <25 | 78.69 | 72.17 | 83.75 |
| Niger | 2012 | Women | No | 25+ | 21.31 | 16.25 | 27.83 |
| Niger | 2012 | Women | Yes | <25 | 80.72 | 62.13 | 96.67 |
| Niger | 2012 | Women | Yes | 25+ | 54.92 | 25.20 | 89.53 |
| Sierra Leone | 2008 | Women | No | <25 | 67.84 | 58.36 | 76.03 |
| Sierra Leone | 2008 | Women | No | 25+ | 32.16 | 23.97 | 41.64 |
| Sierra Leone | 2008 | Women | Yes | <25 | 77.54 | 57.22 | 93.15 |
| Sierra Leone | 2008 | Women | Yes | 25+ | 39.68 | 12.10 | 75.59 |
| Sierra Leone | 2013 | Men | No | <25 | 89.64 | 83.71 | 93.52 |
| Sierra Leone | 2013 | Men | No | 25+ | 10.36 | 6.48 | 16.29 |
| Sierra Leone | 2013 | Men | Yes | <25 | 89.17 | 75.91 | 97.19 |
| Sierra Leone | 2013 | Men | Yes | 25+ | 29.58 | 7.66 | 65.82 |
| Sierra Leone | 2013 | Women | No | <25 | 77.34 | 71.63 | 82.19 |
| Sierra Leone | 2013 | Women | No | 25+ | 22.66 | 17.81 | 28.37 |
| Sierra Leone | 2013 | Women | Yes | <25 | 85.06 | 64.87 | 95.11 |
| Sierra Leone | 2013 | Women | Yes | 25+ | 26.70 | 8.74 | 62.81 |
| Sierra Leone | 2019 | Men | No | <25 | 86.77 | 81.02 | 90.96 |
| Sierra Leone | 2019 | Men | No | 25+ | 13.23 | 9.04 | 18.98 |
| Sierra Leone | 2019 | Men | Yes | <25 | 91.57 | 79.12 | 98.41 |
| Sierra Leone | 2019 | Men | Yes | 25+ | 29.04 | 5.46 | 71.90 |
| Sierra Leone | 2019 | Women | No | <25 | 66.31 | 60.60 | 71.69 |
| Sierra Leone | 2019 | Women | No | 25+ | 33.69 | 28.31 | 39.40 |
| Sierra Leone | 2019 | Women | Yes | <25 | 71.25 | 36.14 | 91.80 |
| Sierra Leone | 2019 | Women | Yes | 25+ | 30.67 | 8.75 | 68.10 |

Overweight is the categorization of body mass index (kg/m^2^).

# **Table P in S1 text. Study-level meta-regression of mean body mass index, and proportion of overweight, on antiretroviral therapy coverage stratified by sex.**

| **Group** | **Predictor** | **Crude** | **Model 1** |
| --- | --- | --- | --- |
| **Outcome: crude mean BMI in latest period *minus* crude mean BMI in earliest period** | | | |
| Men (n=4) | ART coverage (10%) | 0.047 (-1.335; 1.429) | Insufficient observations |
| Women (n=10) |  | 0.198 (-0.034; 0.429) | 0.278 (-0.128; 0.683) |
| **Outcome: difference crude mean BMI between earliest and latest period in HIV- *minus* difference crude mean BMI between earliest and latest period in HIV+** | | | |
| Men (n=4) | ART coverage (10%) | 0.003 (-1.544; 1.549) | Insufficient observations |
| Women (n=10) |  | 0.062 (-0.234; 0.358) | 0.119 (-0.359; 0.598) |
| **Outcome: crude overweight prevalence in latest period *minus* crude overweight prevalence in earliest period** | | | |
| Men (n=4) | ART coverage (10%) | 2.602 (-9.697; 14.901) | Insufficient observations |
| Women (n=10) |  | 2.211 (-0.184; 4.606) | 3.332 (-0.523; 7.187) |
| **Outcome: difference crude overweight prevalence between earliest and latest period in HIV- *minus* difference crude overweight prevalence between earliest and latest period in HIV+** | | | |
| Men (n=4) | ART coverage (10%) | -0.988 (-14.940; 12.964) | Insufficient observations |
| Women (n=10) |  | 0.266 (-2.634; 3.165) | 0.012 (-4.314; 4.338) |

ART: antiretroviral therapy. Study-level meta-regression with *metareg* in *Stata*. The outcomes are depicted in the table in rows with grey background. The exposure was ART coverage (by 10 percentage points to make the regression coefficients meaningful, i.e., observed prevalence of ART coverage divided by 10). Both the outcome and the exposure were at the country level and in the same calendar year. Interpretation: for every 10 percentage points increase in ART coverage, the outcome increased by β. The standard error of the differences was calculated as *sqrt(SE_1_^2^ + SE_2_^2^)*.^2^ Results expressed as coefficients (beta or β) and 95% Confidence Intervals. A p-value <0.05 was considered statistically significant. Crude model only included the outcome and the exposure. Model 1, crude model plus region (East, West, and Southern Africa) and income group (low and low-middle). Results in bold were statistically significant.

# **Table Q in S1 text. Mean body mass index throughout the observation period by socioeconomic quintile and HIV status; not age-SES standardized.**

| **Year** | **Country** | **Sex** | **SES quintile** | **HIV** | **Sample size** | **Mean BMI** | **Lower 95% confidence interval** | **Upper 95% confidence interval** |
| --- | --- | --- | --- | --- | --- | --- | --- | --- |
| 2010 | Burundi | Women | Middle | No | 562 | 21.16 | 20.67 | 21.65 |
| 2010 | Burundi | Women | Middle | Yes | 15 | 20.70 | 19.48 | 21.92 |
| 2010 | Burundi | Women | Poorer | No | 609 | 20.77 | 20.60 | 20.95 |
| 2010 | Burundi | Women | Poorer | Yes | 7 | 19.57 | 18.20 | 20.94 |
| 2010 | Burundi | Women | Poorest | No | 620 | 20.57 | 20.26 | 20.88 |
| 2010 | Burundi | Women | Poorest | Yes | 11 | 19.70 | 18.50 | 20.90 |
| 2010 | Burundi | Women | Richer | No | 569 | 21.25 | 20.90 | 21.61 |
| 2010 | Burundi | Women | Richer | Yes | 5 | 21.25 | 19.11 | 23.39 |
| 2010 | Burundi | Women | Richest | No | 893 | 22.75 | 22.41 | 23.09 |
| 2010 | Burundi | Women | Richest | Yes | 53 | 22.85 | 21.46 | 24.24 |
| 2016 | Burundi | Women | Middle | No | 1234 | 20.67 | 20.52 | 20.82 |
| 2016 | Burundi | Women | Middle | Yes | 10 | 20.65 | 19.29 | 22.02 |
| 2016 | Burundi | Women | Poorer | No | 1197 | 20.38 | 20.23 | 20.53 |
| 2016 | Burundi | Women | Poorer | Yes | 12 | 20.04 | 18.73 | 21.35 |
| 2016 | Burundi | Women | Poorest | No | 1143 | 20.08 | 19.93 | 20.24 |
| 2016 | Burundi | Women | Poorest | Yes | 15 | 19.61 | 17.87 | 21.35 |
| 2016 | Burundi | Women | Richer | No | 1281 | 21.17 | 20.99 | 21.35 |
| 2016 | Burundi | Women | Richer | Yes | 13 | 20.53 | 18.25 | 22.82 |
| 2016 | Burundi | Women | Richest | No | 1613 | 22.95 | 22.65 | 23.26 |
| 2016 | Burundi | Women | Richest | Yes | 64 | 24.62 | 23.08 | 26.16 |
| 2005 | Ethiopia | Women | Middle | No | 697 | 20.07 | 19.85 | 20.28 |
| 2005 | Ethiopia | Women | Middle | Yes | 9 | 20.06 | 16.25 | 23.87 |
| 2005 | Ethiopia | Women | Poorer | No | 721 | 20.11 | 19.88 | 20.35 |
| 2005 | Ethiopia | Women | Poorer | Yes | 7 | 18.83 | 17.86 | 19.79 |
| 2005 | Ethiopia | Women | Poorest | No | 936 | 20.06 | 19.79 | 20.33 |
| 2005 | Ethiopia | Women | Poorest | Yes | 8 | 19.56 | 18.89 | 20.22 |
| 2005 | Ethiopia | Women | Richer | No | 679 | 20.10 | 19.90 | 20.30 |
| 2005 | Ethiopia | Women | Richer | Yes | 7 | 18.01 | 17.39 | 18.64 |
| 2005 | Ethiopia | Women | Richest | No | 1468 | 21.32 | 20.98 | 21.66 |
| 2005 | Ethiopia | Women | Richest | Yes | 99 | 20.74 | 19.54 | 21.93 |
| 2011 | Ethiopia | Men | Middle | No | 1746 | 19.44 | 19.30 | 19.59 |
| 2011 | Ethiopia | Men | Middle | Yes | 15 | 20.14 | 19.17 | 21.11 |
| 2011 | Ethiopia | Men | Poorer | No | 1721 | 19.29 | 19.12 | 19.46 |
| 2011 | Ethiopia | Men | Poorer | Yes | 9 | 18.83 | 17.59 | 20.06 |
| 2011 | Ethiopia | Men | Poorest | No | 2316 | 19.12 | 18.99 | 19.25 |
| 2011 | Ethiopia | Men | Poorest | Yes | 14 | 20.46 | 17.54 | 23.39 |
| 2011 | Ethiopia | Men | Richer | No | 1922 | 19.55 | 19.37 | 19.73 |
| 2011 | Ethiopia | Men | Richer | Yes | 14 | 20.20 | 18.41 | 21.98 |
| 2011 | Ethiopia | Men | Richest | No | 3462 | 20.61 | 20.38 | 20.83 |
| 2011 | Ethiopia | Men | Richest | Yes | 126 | 21.42 | 20.42 | 22.43 |
| 2011 | Ethiopia | Women | Middle | No | 1661 | 20.04 | 19.88 | 20.20 |
| 2011 | Ethiopia | Women | Middle | Yes | 17 | 18.68 | 17.51 | 19.85 |
| 2011 | Ethiopia | Women | Poorer | No | 1783 | 19.89 | 19.70 | 20.07 |
| 2011 | Ethiopia | Women | Poorer | Yes | 13 | 19.69 | 18.28 | 21.11 |
| 2011 | Ethiopia | Women | Poorest | No | 2710 | 19.89 | 19.73 | 20.06 |
| 2011 | Ethiopia | Women | Poorest | Yes | 19 | 20.76 | 18.77 | 22.76 |
| 2011 | Ethiopia | Women | Richer | No | 1757 | 20.13 | 19.96 | 20.31 |
| 2011 | Ethiopia | Women | Richer | Yes | 40 | 19.30 | 17.87 | 20.73 |
| 2011 | Ethiopia | Women | Richest | No | 3841 | 21.82 | 21.58 | 22.05 |
| 2011 | Ethiopia | Women | Richest | Yes | 250 | 20.89 | 20.07 | 21.71 |
| 2016 | Ethiopia | Men | Middle | No | 1383 | 19.61 | 19.45 | 19.77 |
| 2016 | Ethiopia | Men | Middle | Yes | 7 | 18.87 | 17.63 | 20.12 |
| 2016 | Ethiopia | Men | Poorer | No | 1473 | 19.37 | 19.23 | 19.51 |
| 2016 | Ethiopia | Men | Poorer | Yes | 6 | 22.22 | 20.63 | 23.81 |
| 2016 | Ethiopia | Men | Poorest | No | 2269 | 19.36 | 19.21 | 19.52 |
| 2016 | Ethiopia | Men | Poorest | Yes | 17 | 19.55 | 19.17 | 19.92 |
| 2016 | Ethiopia | Men | Richer | No | 1529 | 19.63 | 19.48 | 19.78 |
| 2016 | Ethiopia | Men | Richer | Yes | 8 | 19.55 | 18.05 | 21.06 |
| 2016 | Ethiopia | Men | Richest | No | 3158 | 21.18 | 20.94 | 21.42 |
| 2016 | Ethiopia | Men | Richest | Yes | 75 | 21.24 | 19.85 | 22.63 |
| 2016 | Ethiopia | Women | Middle | No | 1487 | 20.21 | 20.06 | 20.36 |
| 2016 | Ethiopia | Women | Middle | Yes | 13 | 20.96 | 19.08 | 22.85 |
| 2016 | Ethiopia | Women | Poorer | No | 1525 | 20.27 | 20.11 | 20.44 |
| 2016 | Ethiopia | Women | Poorer | Yes | 11 | 20.50 | 18.82 | 22.17 |
| 2016 | Ethiopia | Women | Poorest | No | 2807 | 20.02 | 19.86 | 20.18 |
| 2016 | Ethiopia | Women | Poorest | Yes | 27 | 18.88 | 18.04 | 19.72 |
| 2016 | Ethiopia | Women | Richer | No | 1498 | 20.49 | 20.31 | 20.67 |
| 2016 | Ethiopia | Women | Richer | Yes | 23 | 20.64 | 18.91 | 22.37 |
| 2016 | Ethiopia | Women | Richest | No | 3935 | 22.42 | 21.97 | 22.87 |
| 2016 | Ethiopia | Women | Richest | Yes | 191 | 23.94 | 20.80 | 27.09 |
| 2005 | Guinea | Women | Middle | No | 596 | 21.42 | 21.11 | 21.72 |
| 2005 | Guinea | Women | Middle | Yes | 5 | 20.67 | 18.17 | 23.16 |
| 2005 | Guinea | Women | Poorer | No | 565 | 21.18 | 20.89 | 21.46 |
| 2005 | Guinea | Women | Poorer | Yes | 5 | 18.88 | 15.90 | 21.87 |
| 2005 | Guinea | Women | Poorest | No | 619 | 21.19 | 20.92 | 21.47 |
| 2005 | Guinea | Women | Poorest | Yes | 7 | 20.18 | 17.99 | 22.37 |
| 2005 | Guinea | Women | Richer | No | 609 | 22.33 | 21.89 | 22.78 |
| 2005 | Guinea | Women | Richer | Yes | 25 | 22.40 | 20.54 | 24.27 |
| 2005 | Guinea | Women | Richest | No | 517 | 23.45 | 22.99 | 23.91 |
| 2005 | Guinea | Women | Richest | Yes | 20 | 25.46 | 23.39 | 27.52 |
| 2012 | Guinea | Women | Middle | No | 658 | 22.16 | 21.85 | 22.47 |
| 2012 | Guinea | Women | Middle | Yes | 19 | 23.05 | 19.53 | 26.57 |
| 2012 | Guinea | Women | Poorer | No | 659 | 21.52 | 21.28 | 21.76 |
| 2012 | Guinea | Women | Poorer | Yes | 5 | 20.68 | 20.14 | 21.22 |
| 2012 | Guinea | Women | Poorest | No | 661 | 21.08 | 20.74 | 21.41 |
| 2012 | Guinea | Women | Poorest | Yes | 7 | 19.69 | 19.00 | 20.38 |
| 2012 | Guinea | Women | Richer | No | 835 | 23.43 | 23.03 | 23.83 |
| 2012 | Guinea | Women | Richer | Yes | 24 | 22.37 | 21.19 | 23.56 |
| 2012 | Guinea | Women | Richest | No | 683 | 24.74 | 24.33 | 25.16 |
| 2012 | Guinea | Women | Richest | Yes | 35 | 23.31 | 21.51 | 25.10 |
| 2018 | Guinea | Women | Middle | No | 728 | 23.29 | 22.90 | 23.68 |
| 2018 | Guinea | Women | Middle | Yes | 18 | 23.26 | 21.67 | 24.85 |
| 2018 | Guinea | Women | Poorer | No | 820 | 22.86 | 22.50 | 23.22 |
| 2018 | Guinea | Women | Poorer | Yes | 9 | 22.87 | 21.19 | 24.55 |
| 2018 | Guinea | Women | Poorest | No | 811 | 22.07 | 21.74 | 22.41 |
| 2018 | Guinea | Women | Poorest | Yes | 9 | 20.99 | 19.95 | 22.04 |
| 2018 | Guinea | Women | Richer | No | 779 | 24.98 | 24.45 | 25.51 |
| 2018 | Guinea | Women | Richer | Yes | 21 | 24.14 | 21.49 | 26.80 |
| 2018 | Guinea | Women | Richest | No | 819 | 25.34 | 24.93 | 25.76 |
| 2018 | Guinea | Women | Richest | Yes | 19 | 27.27 | 23.08 | 31.46 |
| 2003 | Kenya | Women | Middle | No | 415 | 22.70 | 22.25 | 23.16 |
| 2003 | Kenya | Women | Middle | Yes | 35 | 21.89 | 20.77 | 23.00 |
| 2003 | Kenya | Women | Poorer | No | 415 | 21.83 | 21.45 | 22.20 |
| 2003 | Kenya | Women | Poorer | Yes | 38 | 21.71 | 20.59 | 22.84 |
| 2003 | Kenya | Women | Poorest | No | 383 | 21.00 | 20.54 | 21.45 |
| 2003 | Kenya | Women | Poorest | Yes | 16 | 19.98 | 17.91 | 22.05 |
| 2003 | Kenya | Women | Richer | No | 447 | 23.78 | 23.31 | 24.25 |
| 2003 | Kenya | Women | Richer | Yes | 56 | 23.06 | 21.17 | 24.96 |
| 2003 | Kenya | Women | Richest | No | 650 | 25.12 | 24.63 | 25.61 |
| 2003 | Kenya | Women | Richest | Yes | 96 | 23.73 | 22.61 | 24.85 |
| 2008 | Kenya | Women | Middle | No | 456 | 22.90 | 22.30 | 23.50 |
| 2008 | Kenya | Women | Middle | Yes | 40 | 22.79 | 21.05 | 24.53 |
| 2008 | Kenya | Women | Poorer | No | 426 | 21.95 | 21.46 | 22.43 |
| 2008 | Kenya | Women | Poorer | Yes | 56 | 21.27 | 20.26 | 22.28 |
| 2008 | Kenya | Women | Poorest | No | 551 | 21.18 | 20.70 | 21.65 |
| 2008 | Kenya | Women | Poorest | Yes | 37 | 20.63 | 19.56 | 21.70 |
| 2008 | Kenya | Women | Richer | No | 553 | 23.56 | 23.06 | 24.06 |
| 2008 | Kenya | Women | Richer | Yes | 51 | 24.66 | 23.01 | 26.30 |
| 2008 | Kenya | Women | Richest | No | 810 | 25.41 | 24.92 | 25.90 |
| 2008 | Kenya | Women | Richest | Yes | 97 | 24.80 | 23.55 | 26.05 |
| 2004 | Lesotho | Women | Middle | No | 297 | 25.40 | 24.61 | 26.20 |
| 2004 | Lesotho | Women | Middle | Yes | 125 | 24.43 | 23.38 | 25.49 |
| 2004 | Lesotho | Women | Poorer | No | 336 | 24.34 | 23.73 | 24.94 |
| 2004 | Lesotho | Women | Poorer | Yes | 153 | 23.73 | 23.00 | 24.46 |
| 2004 | Lesotho | Women | Poorest | No | 301 | 23.86 | 23.35 | 24.36 |
| 2004 | Lesotho | Women | Poorest | Yes | 88 | 23.44 | 22.42 | 24.46 |
| 2004 | Lesotho | Women | Richer | No | 328 | 25.90 | 25.19 | 26.60 |
| 2004 | Lesotho | Women | Richer | Yes | 165 | 25.13 | 24.12 | 26.14 |
| 2004 | Lesotho | Women | Richest | No | 374 | 28.10 | 27.32 | 28.87 |
| 2004 | Lesotho | Women | Richest | Yes | 185 | 26.05 | 25.35 | 26.76 |
| 2009 | Lesotho | Men | Middle | No | 416 | 21.07 | 20.71 | 21.42 |
| 2009 | Lesotho | Men | Middle | Yes | 121 | 20.77 | 20.22 | 21.32 |
| 2009 | Lesotho | Men | Poorer | No | 465 | 20.84 | 20.55 | 21.12 |
| 2009 | Lesotho | Men | Poorer | Yes | 116 | 20.44 | 19.97 | 20.90 |
| 2009 | Lesotho | Men | Poorest | No | 443 | 20.94 | 20.64 | 21.24 |
| 2009 | Lesotho | Men | Poorest | Yes | 95 | 21.19 | 20.54 | 21.84 |
| 2009 | Lesotho | Men | Richer | No | 356 | 21.70 | 21.23 | 22.17 |
| 2009 | Lesotho | Men | Richer | Yes | 101 | 21.89 | 20.76 | 23.02 |
| 2009 | Lesotho | Men | Richest | No | 366 | 22.70 | 22.28 | 23.12 |
| 2009 | Lesotho | Men | Richest | Yes | 96 | 22.73 | 21.94 | 23.53 |
| 2009 | Lesotho | Women | Middle | No | 390 | 25.48 | 24.81 | 26.14 |
| 2009 | Lesotho | Women | Middle | Yes | 192 | 23.97 | 23.11 | 24.82 |
| 2009 | Lesotho | Women | Poorer | No | 415 | 24.97 | 24.36 | 25.59 |
| 2009 | Lesotho | Women | Poorer | Yes | 179 | 24.59 | 23.79 | 25.39 |
| 2009 | Lesotho | Women | Poorest | No | 465 | 23.73 | 23.24 | 24.23 |
| 2009 | Lesotho | Women | Poorest | Yes | 142 | 23.28 | 22.52 | 24.04 |
| 2009 | Lesotho | Women | Richer | No | 414 | 26.12 | 25.51 | 26.73 |
| 2009 | Lesotho | Women | Richer | Yes | 234 | 25.02 | 24.42 | 25.62 |
| 2009 | Lesotho | Women | Richest | No | 482 | 28.57 | 27.87 | 29.27 |
| 2009 | Lesotho | Women | Richest | Yes | 188 | 26.24 | 25.44 | 27.05 |
| 2014 | Lesotho | Men | Middle | No | 380 | 21.46 | 21.13 | 21.80 |
| 2014 | Lesotho | Men | Middle | Yes | 104 | 21.39 | 20.70 | 22.09 |
| 2014 | Lesotho | Men | Poorer | No | 383 | 21.12 | 20.79 | 21.45 |
| 2014 | Lesotho | Men | Poorer | Yes | 84 | 21.28 | 20.62 | 21.94 |
| 2014 | Lesotho | Men | Poorest | No | 332 | 21.31 | 20.96 | 21.66 |
| 2014 | Lesotho | Men | Poorest | Yes | 89 | 20.93 | 20.23 | 21.63 |
| 2014 | Lesotho | Men | Richer | No | 360 | 21.81 | 21.39 | 22.23 |
| 2014 | Lesotho | Men | Richer | Yes | 114 | 21.28 | 20.81 | 21.74 |
| 2014 | Lesotho | Men | Richest | No | 392 | 23.64 | 23.11 | 24.17 |
| 2014 | Lesotho | Men | Richest | Yes | 114 | 23.36 | 22.27 | 24.45 |
| 2014 | Lesotho | Women | Middle | No | 337 | 26.23 | 25.59 | 26.86 |
| 2014 | Lesotho | Women | Middle | Yes | 176 | 25.07 | 24.24 | 25.90 |
| 2014 | Lesotho | Women | Poorer | No | 355 | 25.50 | 24.87 | 26.13 |
| 2014 | Lesotho | Women | Poorer | Yes | 144 | 24.64 | 23.61 | 25.67 |
| 2014 | Lesotho | Women | Poorest | No | 337 | 23.55 | 23.11 | 23.98 |
| 2014 | Lesotho | Women | Poorest | Yes | 137 | 23.33 | 22.62 | 24.04 |
| 2014 | Lesotho | Women | Richer | No | 365 | 28.05 | 27.31 | 28.80 |
| 2014 | Lesotho | Women | Richer | Yes | 213 | 25.75 | 24.94 | 26.56 |
| 2014 | Lesotho | Women | Richest | No | 433 | 27.68 | 26.89 | 28.46 |
| 2014 | Lesotho | Women | Richest | Yes | 203 | 26.75 | 25.94 | 27.56 |
| 2006 | Mali | Women | Middle | No | 678 | 21.83 | 21.49 | 22.18 |
| 2006 | Mali | Women | Middle | Yes | 5 | 21.32 | 19.97 | 22.66 |
| 2006 | Mali | Women | Poorer | No | 659 | 21.51 | 21.25 | 21.76 |
| 2006 | Mali | Women | Poorer | Yes | 7 | 22.67 | 18.68 | 26.66 |
| 2006 | Mali | Women | Poorest | No | 568 | 21.08 | 20.80 | 21.35 |
| 2006 | Mali | Women | Poorest | Yes | 11 | 22.13 | 18.81 | 25.46 |
| 2006 | Mali | Women | Richer | No | 767 | 22.75 | 22.05 | 23.45 |
| 2006 | Mali | Women | Richer | Yes | 17 | 22.25 | 20.13 | 24.37 |
| 2006 | Mali | Women | Richest | No | 764 | 24.48 | 24.07 | 24.90 |
| 2006 | Mali | Women | Richest | Yes | 16 | 24.06 | 21.51 | 26.62 |
| 2012 | Mali | Women | Middle | No | 711 | 21.65 | 21.37 | 21.93 |
| 2012 | Mali | Women | Middle | Yes | 10 | 21.64 | 20.22 | 23.06 |
| 2012 | Mali | Women | Poorer | No | 717 | 21.73 | 21.45 | 22.01 |
| 2012 | Mali | Women | Poorer | Yes | 1 | 23.51 | 23.51 | 23.51 |
| 2012 | Mali | Women | Poorest | No | 749 | 21.53 | 21.27 | 21.79 |
| 2012 | Mali | Women | Poorest | Yes | 7 | 22.05 | 18.73 | 25.37 |
| 2012 | Mali | Women | Richer | No | 749 | 22.75 | 22.40 | 23.11 |
| 2012 | Mali | Women | Richer | Yes | 10 | 24.41 | 20.88 | 27.94 |
| 2012 | Mali | Women | Richest | No | 1040 | 24.62 | 24.23 | 25.01 |
| 2012 | Mali | Women | Richest | Yes | 22 | 25.20 | 22.36 | 28.05 |
| 2004 | Malawi | Women | Middle | No | 410 | 21.75 | 21.40 | 22.10 |
| 2004 | Malawi | Women | Middle | Yes | 81 | 21.20 | 20.56 | 21.83 |
| 2004 | Malawi | Women | Poorer | No | 399 | 21.83 | 21.54 | 22.12 |
| 2004 | Malawi | Women | Poorer | Yes | 57 | 20.61 | 19.92 | 21.31 |
| 2004 | Malawi | Women | Poorest | No | 337 | 21.77 | 21.42 | 22.13 |
| 2004 | Malawi | Women | Poorest | Yes | 56 | 21.78 | 20.22 | 23.34 |
| 2004 | Malawi | Women | Richer | No | 374 | 22.44 | 22.01 | 22.87 |
| 2004 | Malawi | Women | Richer | Yes | 87 | 22.11 | 21.30 | 22.91 |
| 2004 | Malawi | Women | Richest | No | 299 | 23.97 | 23.34 | 24.59 |
| 2004 | Malawi | Women | Richest | Yes | 90 | 23.67 | 22.94 | 24.41 |
| 2010 | Malawi | Women | Middle | No | 977 | 22.32 | 22.07 | 22.56 |
| 2010 | Malawi | Women | Middle | Yes | 137 | 21.56 | 20.92 | 22.20 |
| 2010 | Malawi | Women | Poorer | No | 1020 | 22.23 | 21.90 | 22.57 |
| 2010 | Malawi | Women | Poorer | Yes | 132 | 21.97 | 21.46 | 22.49 |
| 2010 | Malawi | Women | Poorest | No | 907 | 21.86 | 21.63 | 22.10 |
| 2010 | Malawi | Women | Poorest | Yes | 110 | 21.31 | 20.79 | 21.84 |
| 2010 | Malawi | Women | Richer | No | 1001 | 22.68 | 22.44 | 22.92 |
| 2010 | Malawi | Women | Richer | Yes | 173 | 22.83 | 22.18 | 23.49 |
| 2010 | Malawi | Women | Richest | No | 896 | 24.05 | 23.63 | 24.47 |
| 2010 | Malawi | Women | Richest | Yes | 244 | 24.14 | 23.36 | 24.93 |
| 2015 | Malawi | Women | Middle | No | 1008 | 22.66 | 22.42 | 22.90 |
| 2015 | Malawi | Women | Middle | Yes | 135 | 22.70 | 21.90 | 23.51 |
| 2015 | Malawi | Women | Poorer | No | 1034 | 22.28 | 22.07 | 22.50 |
| 2015 | Malawi | Women | Poorer | Yes | 112 | 22.21 | 21.41 | 23.02 |
| 2015 | Malawi | Women | Poorest | No | 918 | 21.93 | 21.65 | 22.20 |
| 2015 | Malawi | Women | Poorest | Yes | 123 | 21.73 | 21.13 | 22.32 |
| 2015 | Malawi | Women | Richer | No | 1081 | 23.24 | 22.90 | 23.57 |
| 2015 | Malawi | Women | Richer | Yes | 157 | 23.67 | 22.75 | 24.60 |
| 2015 | Malawi | Women | Richest | No | 1343 | 24.85 | 24.52 | 25.18 |
| 2015 | Malawi | Women | Richest | Yes | 256 | 24.68 | 23.87 | 25.48 |
| 2006 | Niger | Women | Middle | No | 570 | 20.84 | 20.55 | 21.13 |
| 2006 | Niger | Women | Middle | Yes | 1 | 22.38 | 22.38 | 22.38 |
| 2006 | Niger | Women | Poorer | No | 515 | 20.76 | 20.49 | 21.04 |
| 2006 | Niger | Women | Poorer | Yes | 3 | 19.68 | 17.52 | 21.85 |
| 2006 | Niger | Women | Poorest | No | 524 | 20.87 | 20.61 | 21.13 |
| 2006 | Niger | Women | Poorest | Yes | 3 | 20.04 | 17.15 | 22.92 |
| 2006 | Niger | Women | Richer | No | 608 | 21.40 | 21.05 | 21.75 |
| 2006 | Niger | Women | Richer | Yes | 9 | 23.18 | 20.88 | 25.48 |
| 2006 | Niger | Women | Richest | No | 1046 | 24.04 | 23.63 | 24.45 |
| 2006 | Niger | Women | Richest | Yes | 18 | 24.11 | 22.21 | 26.00 |
| 2012 | Niger | Women | Middle | No | 672 | 21.50 | 21.19 | 21.80 |
| 2012 | Niger | Women | Middle | Yes | 1 | 15.74 | 15.74 | 15.74 |
| 2012 | Niger | Women | Poorer | No | 634 | 21.40 | 21.08 | 21.72 |
| 2012 | Niger | Women | Poorer | Yes | 2 | 19.05 | 13.73 | 24.36 |
| 2012 | Niger | Women | Poorest | No | 632 | 21.71 | 21.04 | 22.37 |
| 2012 | Niger | Women | Poorest | Yes | 2 | 20.41 | 20.16 | 20.67 |
| 2012 | Niger | Women | Richer | No | 761 | 21.98 | 21.64 | 22.32 |
| 2012 | Niger | Women | Richer | Yes | 6 | 25.45 | 21.78 | 29.12 |
| 2012 | Niger | Women | Richest | No | 1146 | 24.68 | 24.33 | 25.02 |
| 2012 | Niger | Women | Richest | Yes | 13 | 24.21 | 20.93 | 27.50 |
| 2008 | Sierra Leone | Women | Middle | No | 505 | 23.32 | 22.72 | 23.91 |
| 2008 | Sierra Leone | Women | Middle | Yes | 7 | 21.54 | 19.93 | 23.15 |
| 2008 | Sierra Leone | Women | Poorer | No | 490 | 22.59 | 22.01 | 23.18 |
| 2008 | Sierra Leone | Women | Poorer | Yes | 12 | 23.80 | 19.94 | 27.66 |
| 2008 | Sierra Leone | Women | Poorest | No | 527 | 23.25 | 22.50 | 24.01 |
| 2008 | Sierra Leone | Women | Poorest | Yes | 3 | 18.67 | 18.14 | 19.20 |
| 2008 | Sierra Leone | Women | Richer | No | 589 | 23.96 | 22.99 | 24.93 |
| 2008 | Sierra Leone | Women | Richer | Yes | 15 | 21.98 | 19.20 | 24.76 |
| 2008 | Sierra Leone | Women | Richest | No | 662 | 25.25 | 24.64 | 25.86 |
| 2008 | Sierra Leone | Women | Richest | Yes | 20 | 23.56 | 21.31 | 25.81 |
| 2013 | Sierra Leone | Men | Middle | No | 1030 | 21.64 | 21.36 | 21.91 |
| 2013 | Sierra Leone | Men | Middle | Yes | 8 | 22.33 | 21.30 | 23.36 |
| 2013 | Sierra Leone | Men | Poorer | No | 1029 | 21.38 | 21.10 | 21.66 |
| 2013 | Sierra Leone | Men | Poorer | Yes | 8 | 20.23 | 19.55 | 20.91 |
| 2013 | Sierra Leone | Men | Poorest | No | 1088 | 21.36 | 21.14 | 21.58 |
| 2013 | Sierra Leone | Men | Poorest | Yes | 9 | 20.76 | 19.70 | 21.81 |
| 2013 | Sierra Leone | Men | Richer | No | 1229 | 21.69 | 21.45 | 21.93 |
| 2013 | Sierra Leone | Men | Richer | Yes | 17 | 22.11 | 20.51 | 23.71 |
| 2013 | Sierra Leone | Men | Richest | No | 1342 | 22.22 | 21.93 | 22.51 |
| 2013 | Sierra Leone | Men | Richest | Yes | 30 | 22.54 | 21.54 | 23.54 |
| 2013 | Sierra Leone | Women | Middle | No | 1108 | 22.27 | 21.99 | 22.56 |
| 2013 | Sierra Leone | Women | Middle | Yes | 14 | 23.46 | 21.36 | 25.56 |
| 2013 | Sierra Leone | Women | Poorer | No | 1029 | 22.01 | 21.73 | 22.28 |
| 2013 | Sierra Leone | Women | Poorer | Yes | 13 | 21.22 | 19.49 | 22.95 |
| 2013 | Sierra Leone | Women | Poorest | No | 1122 | 21.91 | 21.59 | 22.23 |
| 2013 | Sierra Leone | Women | Poorest | Yes | 11 | 21.52 | 19.95 | 23.09 |
| 2013 | Sierra Leone | Women | Richer | No | 1407 | 23.02 | 22.71 | 23.34 |
| 2013 | Sierra Leone | Women | Richer | Yes | 36 | 23.29 | 22.30 | 24.29 |
| 2013 | Sierra Leone | Women | Richest | No | 1329 | 24.42 | 24.00 | 24.83 |
| 2013 | Sierra Leone | Women | Richest | Yes | 46 | 24.59 | 23.12 | 26.06 |
| 2019 | Sierra Leone | Men | Middle | No | 978 | 21.75 | 21.53 | 21.98 |
| 2019 | Sierra Leone | Men | Middle | Yes | 14 | 21.35 | 19.58 | 23.11 |
| 2019 | Sierra Leone | Men | Poorer | No | 989 | 21.43 | 21.29 | 21.57 |
| 2019 | Sierra Leone | Men | Poorer | Yes | 9 | 21.63 | 19.19 | 24.07 |
| 2019 | Sierra Leone | Men | Poorest | No | 1129 | 21.43 | 21.22 | 21.64 |
| 2019 | Sierra Leone | Men | Poorest | Yes | 7 | 20.38 | 19.26 | 21.49 |
| 2019 | Sierra Leone | Men | Richer | No | 1054 | 22.03 | 21.82 | 22.25 |
| 2019 | Sierra Leone | Men | Richer | Yes | 14 | 21.30 | 20.45 | 22.15 |
| 2019 | Sierra Leone | Men | Richest | No | 960 | 22.94 | 22.66 | 23.23 |
| 2019 | Sierra Leone | Men | Richest | Yes | 23 | 22.79 | 22.02 | 23.57 |
| 2019 | Sierra Leone | Women | Middle | No | 1147 | 23.33 | 23.02 | 23.64 |
| 2019 | Sierra Leone | Women | Middle | Yes | 22 | 23.07 | 20.81 | 25.32 |
| 2019 | Sierra Leone | Women | Poorer | No | 1086 | 22.55 | 22.24 | 22.86 |
| 2019 | Sierra Leone | Women | Poorer | Yes | 19 | 21.63 | 20.38 | 22.88 |
| 2019 | Sierra Leone | Women | Poorest | No | 1185 | 22.65 | 22.40 | 22.90 |
| 2019 | Sierra Leone | Women | Poorest | Yes | 14 | 22.49 | 20.79 | 24.19 |
| 2019 | Sierra Leone | Women | Richer | No | 1199 | 24.43 | 24.11 | 24.76 |
| 2019 | Sierra Leone | Women | Richer | Yes | 39 | 23.19 | 21.68 | 24.69 |
| 2019 | Sierra Leone | Women | Richest | No | 1038 | 25.62 | 25.22 | 26.03 |
| 2019 | Sierra Leone | Women | Richest | Yes | 46 | 23.58 | 22.18 | 24.99 |
| 2005 | Zimbabwe | Women | Middle | No | 806 | 23.08 | 22.79 | 23.36 |
| 2005 | Zimbabwe | Women | Middle | Yes | 275 | 21.95 | 21.57 | 22.32 |
| 2005 | Zimbabwe | Women | Poorer | No | 850 | 22.56 | 22.25 | 22.88 |
| 2005 | Zimbabwe | Women | Poorer | Yes | 269 | 21.89 | 21.50 | 22.29 |
| 2005 | Zimbabwe | Women | Poorest | No | 878 | 22.05 | 21.80 | 22.30 |
| 2005 | Zimbabwe | Women | Poorest | Yes | 242 | 21.47 | 21.06 | 21.88 |
| 2005 | Zimbabwe | Women | Richer | No | 884 | 24.28 | 23.89 | 24.68 |
| 2005 | Zimbabwe | Women | Richer | Yes | 364 | 23.27 | 22.81 | 23.73 |
| 2005 | Zimbabwe | Women | Richest | No | 1080 | 25.03 | 24.76 | 25.31 |
| 2005 | Zimbabwe | Women | Richest | Yes | 260 | 24.72 | 24.12 | 25.32 |
| 2010 | Zimbabwe | Men | Middle | No | 838 | 21.25 | 21.04 | 21.45 |
| 2010 | Zimbabwe | Men | Middle | Yes | 151 | 20.47 | 20.04 | 20.90 |
| 2010 | Zimbabwe | Men | Poorer | No | 771 | 20.99 | 20.78 | 21.21 |
| 2010 | Zimbabwe | Men | Poorer | Yes | 150 | 20.49 | 20.08 | 20.91 |
| 2010 | Zimbabwe | Men | Poorest | No | 787 | 20.97 | 20.72 | 21.22 |
| 2010 | Zimbabwe | Men | Poorest | Yes | 179 | 20.43 | 20.02 | 20.85 |
| 2010 | Zimbabwe | Men | Richer | No | 968 | 21.87 | 21.67 | 22.07 |
| 2010 | Zimbabwe | Men | Richer | Yes | 165 | 21.41 | 20.81 | 22.01 |
| 2010 | Zimbabwe | Men | Richest | No | 967 | 22.66 | 22.26 | 23.05 |
| 2010 | Zimbabwe | Men | Richest | Yes | 141 | 22.06 | 21.49 | 22.63 |
| 2010 | Zimbabwe | Women | Middle | No | 862 | 24.01 | 23.69 | 24.33 |
| 2010 | Zimbabwe | Women | Middle | Yes | 269 | 23.82 | 23.22 | 24.43 |
| 2010 | Zimbabwe | Women | Poorer | No | 863 | 23.46 | 23.16 | 23.77 |
| 2010 | Zimbabwe | Women | Poorer | Yes | 223 | 22.81 | 22.26 | 23.36 |
| 2010 | Zimbabwe | Women | Poorest | No | 955 | 22.77 | 22.44 | 23.09 |
| 2010 | Zimbabwe | Women | Poorest | Yes | 267 | 22.18 | 21.74 | 22.61 |
| 2010 | Zimbabwe | Women | Richer | No | 1062 | 24.87 | 24.53 | 25.20 |
| 2010 | Zimbabwe | Women | Richer | Yes | 322 | 24.19 | 23.69 | 24.69 |
| 2010 | Zimbabwe | Women | Richest | No | 1162 | 25.80 | 25.43 | 26.17 |
| 2010 | Zimbabwe | Women | Richest | Yes | 255 | 25.47 | 24.81 | 26.12 |
| 2015 | Zimbabwe | Men | Middle | No | 898 | 21.50 | 21.31 | 21.69 |
| 2015 | Zimbabwe | Men | Middle | Yes | 137 | 21.04 | 20.62 | 21.46 |
| 2015 | Zimbabwe | Men | Poorer | No | 834 | 21.21 | 21.03 | 21.39 |
| 2015 | Zimbabwe | Men | Poorer | Yes | 148 | 20.77 | 20.30 | 21.25 |
| 2015 | Zimbabwe | Men | Poorest | No | 756 | 21.19 | 21.01 | 21.37 |
| 2015 | Zimbabwe | Men | Poorest | Yes | 137 | 20.15 | 19.81 | 20.50 |
| 2015 | Zimbabwe | Men | Richer | No | 1352 | 22.36 | 22.17 | 22.56 |
| 2015 | Zimbabwe | Men | Richer | Yes | 219 | 21.47 | 20.98 | 21.95 |
| 2015 | Zimbabwe | Men | Richest | No | 1504 | 23.65 | 23.33 | 23.97 |
| 2015 | Zimbabwe | Men | Richest | Yes | 214 | 22.75 | 21.99 | 23.50 |
| 2015 | Zimbabwe | Women | Middle | No | 877 | 24.53 | 24.22 | 24.83 |
| 2015 | Zimbabwe | Women | Middle | Yes | 258 | 23.61 | 23.03 | 24.18 |
| 2015 | Zimbabwe | Women | Poorer | No | 844 | 23.71 | 23.42 | 24.00 |
| 2015 | Zimbabwe | Women | Poorer | Yes | 194 | 22.74 | 22.18 | 23.30 |
| 2015 | Zimbabwe | Women | Poorest | No | 835 | 23.17 | 22.93 | 23.41 |
| 2015 | Zimbabwe | Women | Poorest | Yes | 248 | 22.45 | 21.95 | 22.95 |
| 2015 | Zimbabwe | Women | Richer | No | 1485 | 25.65 | 25.33 | 25.98 |
| 2015 | Zimbabwe | Women | Richer | Yes | 433 | 24.49 | 23.82 | 25.16 |
| 2015 | Zimbabwe | Women | Richest | No | 1783 | 26.78 | 26.42 | 27.14 |
| 2015 | Zimbabwe | Women | Richest | Yes | 327 | 26.44 | 25.73 | 27.16 |

SES: socioeconomic status.

# **Table R in S1 text. Prevalence of overweight throughout the observation period by socioeconomic quintile and HIV status; not age-SES standardized.**

| **Year** | **Country** | **Sex** | **SES quantile** | **HIV** | **BMI group** | **Sample size** | **Prevalence** | **95% CI, lower** | **95% CI, upper** |
| --- | --- | --- | --- | --- | --- | --- | --- | --- | --- |
| 2010 | Burundi | Women | Middle | No | <25 | 526 | 93.43 | 90.52 | 95.50 |
| 2010 | Burundi | Women | Middle | No | 25+ | 36 | 6.57 | 4.50 | 9.48 |
| 2010 | Burundi | Women | Middle | Yes | <25 | 15 | 100.00 | 100.00 | 100.00 |
| 2010 | Burundi | Women | Poorer | No | <25 | 588 | 96.69 | 94.86 | 97.89 |
| 2010 | Burundi | Women | Poorer | No | 25+ | 21 | 3.31 | 2.11 | 5.14 |
| 2010 | Burundi | Women | Poorer | Yes | <25 | 7 | 100.00 | 100.00 | 100.00 |
| 2010 | Burundi | Women | Poorest | No | <25 | 598 | 96.42 | 94.49 | 97.69 |
| 2010 | Burundi | Women | Poorest | No | 25+ | 22 | 3.58 | 2.31 | 5.51 |
| 2010 | Burundi | Women | Poorest | Yes | <25 | 10 | 94.01 | 69.00 | 99.10 |
| 2010 | Burundi | Women | Poorest | Yes | 25+ | 1 | 5.99 | 0.90 | 31.00 |
| 2010 | Burundi | Women | Richer | No | <25 | 527 | 92.62 | 90.16 | 94.50 |
| 2010 | Burundi | Women | Richer | No | 25+ | 42 | 7.38 | 5.50 | 9.84 |
| 2010 | Burundi | Women | Richer | Yes | <25 | 5 | 100.00 | 100.00 | 100.00 |
| 2010 | Burundi | Women | Richest | No | <25 | 667 | 78.72 | 75.45 | 81.66 |
| 2010 | Burundi | Women | Richest | No | 25+ | 226 | 21.28 | 18.34 | 24.55 |
| 2010 | Burundi | Women | Richest | Yes | <25 | 36 | 73.55 | 58.33 | 84.68 |
| 2010 | Burundi | Women | Richest | Yes | 25+ | 17 | 26.45 | 15.32 | 41.67 |
| 2016 | Burundi | Women | Middle | No | <25 | 1183 | 95.68 | 94.06 | 96.87 |
| 2016 | Burundi | Women | Middle | No | 25+ | 51 | 4.32 | 3.13 | 5.94 |
| 2016 | Burundi | Women | Middle | Yes | <25 | 10 | 100.00 | 100.00 | 100.00 |
| 2016 | Burundi | Women | Poorer | No | <25 | 1160 | 97.21 | 96.05 | 98.03 |
| 2016 | Burundi | Women | Poorer | No | 25+ | 37 | 2.79 | 1.97 | 3.95 |
| 2016 | Burundi | Women | Poorer | Yes | <25 | 11 | 95.78 | 73.66 | 99.46 |
| 2016 | Burundi | Women | Poorer | Yes | 25+ | 1 | 4.22 | 0.54 | 26.34 |
| 2016 | Burundi | Women | Poorest | No | <25 | 1118 | 97.66 | 96.53 | 98.43 |
| 2016 | Burundi | Women | Poorest | No | 25+ | 25 | 2.34 | 1.57 | 3.47 |
| 2016 | Burundi | Women | Poorest | Yes | <25 | 15 | 100.00 | 100.00 | 100.00 |
| 2016 | Burundi | Women | Richer | No | <25 | 1183 | 92.08 | 89.81 | 93.87 |
| 2016 | Burundi | Women | Richer | No | 25+ | 98 | 7.92 | 6.13 | 10.19 |
| 2016 | Burundi | Women | Richer | Yes | <25 | 11 | 85.87 | 53.15 | 97.02 |
| 2016 | Burundi | Women | Richer | Yes | 25+ | 2 | 14.13 | 2.98 | 46.85 |
| 2016 | Burundi | Women | Richest | No | <25 | 1196 | 75.76 | 72.75 | 78.54 |
| 2016 | Burundi | Women | Richest | No | 25+ | 417 | 24.24 | 21.46 | 27.25 |
| 2016 | Burundi | Women | Richest | Yes | <25 | 41 | 65.69 | 52.40 | 76.90 |
| 2016 | Burundi | Women | Richest | Yes | 25+ | 23 | 34.31 | 23.10 | 47.60 |
| 2005 | Ethiopia | Women | Middle | No | <25 | 687 | 98.28 | 96.68 | 99.11 |
| 2005 | Ethiopia | Women | Middle | No | 25+ | 10 | 1.72 | 0.89 | 3.32 |
| 2005 | Ethiopia | Women | Middle | Yes | <25 | 8 | 82.22 | 28.96 | 98.13 |
| 2005 | Ethiopia | Women | Middle | Yes | 25+ | 1 | 17.78 | 1.87 | 71.04 |
| 2005 | Ethiopia | Women | Poorer | No | <25 | 701 | 96.24 | 93.94 | 97.69 |
| 2005 | Ethiopia | Women | Poorer | No | 25+ | 20 | 3.76 | 2.31 | 6.06 |
| 2005 | Ethiopia | Women | Poorer | Yes | <25 | 7 | 100.00 | 100.00 | 100.00 |
| 2005 | Ethiopia | Women | Poorest | No | <25 | 917 | 98.07 | 96.34 | 98.99 |
| 2005 | Ethiopia | Women | Poorest | No | 25+ | 19 | 1.93 | 1.01 | 3.66 |
| 2005 | Ethiopia | Women | Poorest | Yes | <25 | 8 | 100.00 | 100.00 | 100.00 |
| 2005 | Ethiopia | Women | Richer | No | <25 | 667 | 98.14 | 96.36 | 99.06 |
| 2005 | Ethiopia | Women | Richer | No | 25+ | 12 | 1.86 | 0.94 | 3.64 |
| 2005 | Ethiopia | Women | Richer | Yes | <25 | 7 | 100.00 | 100.00 | 100.00 |
| 2005 | Ethiopia | Women | Richest | No | <25 | 1242 | 88.38 | 85.06 | 91.05 |
| 2005 | Ethiopia | Women | Richest | No | 25+ | 226 | 11.62 | 8.95 | 14.94 |
| 2005 | Ethiopia | Women | Richest | Yes | <25 | 90 | 91.60 | 71.84 | 97.90 |
| 2005 | Ethiopia | Women | Richest | Yes | 25+ | 9 | 8.40 | 2.10 | 28.16 |
| 2011 | Ethiopia | Men | Middle | No | <25 | 1734 | 99.35 | 98.70 | 99.68 |
| 2011 | Ethiopia | Men | Middle | No | 25+ | 12 | 0.65 | 0.32 | 1.30 |
| 2011 | Ethiopia | Men | Middle | Yes | <25 | 15 | 100.00 | 100.00 | 100.00 |
| 2011 | Ethiopia | Men | Poorer | No | <25 | 1713 | 99.68 | 99.19 | 99.88 |
| 2011 | Ethiopia | Men | Poorer | No | 25+ | 8 | 0.32 | 0.12 | 0.81 |
| 2011 | Ethiopia | Men | Poorer | Yes | <25 | 9 | 100.00 | 100.00 | 100.00 |
| 2011 | Ethiopia | Men | Poorest | No | <25 | 2307 | 99.22 | 98.38 | 99.62 |
| 2011 | Ethiopia | Men | Poorest | No | 25+ | 9 | 0.78 | 0.38 | 1.62 |
| 2011 | Ethiopia | Men | Poorest | Yes | <25 | 13 | 86.50 | 39.70 | 98.42 |
| 2011 | Ethiopia | Men | Poorest | Yes | 25+ | 1 | 13.50 | 1.58 | 60.30 |
| 2011 | Ethiopia | Men | Richer | No | <25 | 1893 | 98.69 | 97.93 | 99.18 |
| 2011 | Ethiopia | Men | Richer | No | 25+ | 29 | 1.31 | 0.82 | 2.07 |
| 2011 | Ethiopia | Men | Richer | Yes | <25 | 14 | 100.00 | 100.00 | 100.00 |
| 2011 | Ethiopia | Men | Richest | No | <25 | 3088 | 90.89 | 88.66 | 92.72 |
| 2011 | Ethiopia | Men | Richest | No | 25+ | 374 | 9.11 | 7.28 | 11.34 |
| 2011 | Ethiopia | Men | Richest | Yes | <25 | 108 | 88.87 | 78.49 | 94.59 |
| 2011 | Ethiopia | Men | Richest | Yes | 25+ | 18 | 11.13 | 5.41 | 21.51 |
| 2011 | Ethiopia | Women | Middle | No | <25 | 1624 | 97.71 | 96.61 | 98.47 |
| 2011 | Ethiopia | Women | Middle | No | 25+ | 37 | 2.29 | 1.53 | 3.39 |
| 2011 | Ethiopia | Women | Middle | Yes | <25 | 17 | 100.00 | 100.00 | 100.00 |
| 2011 | Ethiopia | Women | Poorer | No | <25 | 1746 | 97.36 | 95.98 | 98.28 |
| 2011 | Ethiopia | Women | Poorer | No | 25+ | 37 | 2.64 | 1.72 | 4.02 |
| 2011 | Ethiopia | Women | Poorer | Yes | <25 | 13 | 100.00 | 100.00 | 100.00 |
| 2011 | Ethiopia | Women | Poorest | No | <25 | 2662 | 97.92 | 97.02 | 98.56 |
| 2011 | Ethiopia | Women | Poorest | No | 25+ | 48 | 2.08 | 1.44 | 2.98 |
| 2011 | Ethiopia | Women | Poorest | Yes | <25 | 19 | 100.00 | 100.00 | 100.00 |
| 2011 | Ethiopia | Women | Richer | No | <25 | 1696 | 97.32 | 96.17 | 98.13 |
| 2011 | Ethiopia | Women | Richer | No | 25+ | 61 | 2.68 | 1.87 | 3.83 |
| 2011 | Ethiopia | Women | Richer | Yes | <25 | 38 | 96.06 | 80.66 | 99.30 |
| 2011 | Ethiopia | Women | Richer | Yes | 25+ | 2 | 3.94 | 0.70 | 19.34 |
| 2011 | Ethiopia | Women | Richest | No | <25 | 3051 | 82.49 | 79.83 | 84.87 |
| 2011 | Ethiopia | Women | Richest | No | 25+ | 790 | 17.51 | 15.13 | 20.17 |
| 2011 | Ethiopia | Women | Richest | Yes | <25 | 206 | 86.68 | 79.08 | 91.80 |
| 2011 | Ethiopia | Women | Richest | Yes | 25+ | 44 | 13.32 | 8.20 | 20.92 |
| 2016 | Ethiopia | Men | Middle | No | <25 | 1372 | 99.19 | 98.34 | 99.61 |
| 2016 | Ethiopia | Men | Middle | No | 25+ | 11 | 0.81 | 0.39 | 1.66 |
| 2016 | Ethiopia | Men | Middle | Yes | <25 | 7 | 100.00 | 100.00 | 100.00 |
| 2016 | Ethiopia | Men | Poorer | No | <25 | 1460 | 98.90 | 97.86 | 99.44 |
| 2016 | Ethiopia | Men | Poorer | No | 25+ | 13 | 1.10 | 0.56 | 2.14 |
| 2016 | Ethiopia | Men | Poorer | Yes | <25 | 6 | 100.00 | 100.00 | 100.00 |
| 2016 | Ethiopia | Men | Poorest | No | <25 | 2243 | 98.75 | 97.73 | 99.32 |
| 2016 | Ethiopia | Men | Poorest | No | 25+ | 26 | 1.25 | 0.68 | 2.27 |
| 2016 | Ethiopia | Men | Poorest | Yes | <25 | 17 | 100.00 | 100.00 | 100.00 |
| 2016 | Ethiopia | Men | Richer | No | <25 | 1509 | 99.22 | 98.53 | 99.59 |
| 2016 | Ethiopia | Men | Richer | No | 25+ | 20 | 0.78 | 0.41 | 1.47 |
| 2016 | Ethiopia | Men | Richer | Yes | <25 | 8 | 100.00 | 100.00 | 100.00 |
| 2016 | Ethiopia | Men | Richest | No | <25 | 2665 | 87.50 | 85.51 | 89.25 |
| 2016 | Ethiopia | Men | Richest | No | 25+ | 493 | 12.50 | 10.75 | 14.49 |
| 2016 | Ethiopia | Men | Richest | Yes | <25 | 67 | 91.19 | 68.80 | 97.98 |
| 2016 | Ethiopia | Men | Richest | Yes | 25+ | 8 | 8.81 | 2.02 | 31.20 |
| 2016 | Ethiopia | Women | Middle | No | <25 | 1435 | 97.32 | 96.25 | 98.08 |
| 2016 | Ethiopia | Women | Middle | No | 25+ | 52 | 2.68 | 1.92 | 3.75 |
| 2016 | Ethiopia | Women | Middle | Yes | <25 | 12 | 99.47 | 95.77 | 99.94 |
| 2016 | Ethiopia | Women | Middle | Yes | 25+ | 1 | 0.53 | 0.06 | 4.23 |
| 2016 | Ethiopia | Women | Poorer | No | <25 | 1474 | 97.24 | 95.98 | 98.12 |
| 2016 | Ethiopia | Women | Poorer | No | 25+ | 51 | 2.76 | 1.88 | 4.02 |
| 2016 | Ethiopia | Women | Poorer | Yes | <25 | 11 | 100.00 | 100.00 | 100.00 |
| 2016 | Ethiopia | Women | Poorest | No | <25 | 2688 | 96.90 | 95.78 | 97.73 |
| 2016 | Ethiopia | Women | Poorest | No | 25+ | 119 | 3.10 | 2.27 | 4.22 |
| 2016 | Ethiopia | Women | Poorest | Yes | <25 | 26 | 99.68 | 97.46 | 99.96 |
| 2016 | Ethiopia | Women | Poorest | Yes | 25+ | 1 | 0.32 | 0.04 | 2.54 |
| 2016 | Ethiopia | Women | Richer | No | <25 | 1401 | 95.47 | 93.83 | 96.69 |
| 2016 | Ethiopia | Women | Richer | No | 25+ | 97 | 4.53 | 3.31 | 6.17 |
| 2016 | Ethiopia | Women | Richer | Yes | <25 | 20 | 89.86 | 54.08 | 98.52 |
| 2016 | Ethiopia | Women | Richer | Yes | 25+ | 3 | 10.14 | 1.48 | 45.92 |
| 2016 | Ethiopia | Women | Richest | No | <25 | 2883 | 78.88 | 75.27 | 82.08 |
| 2016 | Ethiopia | Women | Richest | No | 25+ | 1052 | 21.12 | 17.92 | 24.73 |
| 2016 | Ethiopia | Women | Richest | Yes | <25 | 130 | 67.02 | 49.62 | 80.74 |
| 2016 | Ethiopia | Women | Richest | Yes | 25+ | 61 | 32.98 | 19.26 | 50.38 |
| 2005 | Guinea | Women | Middle | No | <25 | 536 | 90.61 | 87.79 | 92.84 |
| 2005 | Guinea | Women | Middle | No | 25+ | 60 | 9.39 | 7.16 | 12.21 |
| 2005 | Guinea | Women | Middle | Yes | <25 | 4 | 78.13 | 27.87 | 97.06 |
| 2005 | Guinea | Women | Middle | Yes | 25+ | 1 | 21.87 | 2.94 | 72.13 |
| 2005 | Guinea | Women | Poorer | No | <25 | 523 | 91.67 | 88.32 | 94.13 |
| 2005 | Guinea | Women | Poorer | No | 25+ | 42 | 8.33 | 5.87 | 11.68 |
| 2005 | Guinea | Women | Poorer | Yes | <25 | 5 | 100.00 | 100.00 | 100.00 |
| 2005 | Guinea | Women | Poorest | No | <25 | 570 | 92.03 | 89.50 | 93.98 |
| 2005 | Guinea | Women | Poorest | No | 25+ | 49 | 7.97 | 6.02 | 10.50 |
| 2005 | Guinea | Women | Poorest | Yes | <25 | 6 | 90.88 | 52.36 | 98.91 |
| 2005 | Guinea | Women | Poorest | Yes | 25+ | 1 | 9.12 | 1.09 | 47.64 |
| 2005 | Guinea | Women | Richer | No | <25 | 497 | 80.92 | 76.73 | 84.50 |
| 2005 | Guinea | Women | Richer | No | 25+ | 112 | 19.08 | 15.50 | 23.27 |
| 2005 | Guinea | Women | Richer | Yes | <25 | 22 | 82.23 | 53.04 | 94.99 |
| 2005 | Guinea | Women | Richer | Yes | 25+ | 3 | 17.77 | 5.01 | 46.96 |
| 2005 | Guinea | Women | Richest | No | <25 | 359 | 68.37 | 63.89 | 72.53 |
| 2005 | Guinea | Women | Richest | No | 25+ | 158 | 31.63 | 27.47 | 36.11 |
| 2005 | Guinea | Women | Richest | Yes | <25 | 12 | 51.28 | 32.21 | 69.98 |
| 2005 | Guinea | Women | Richest | Yes | 25+ | 8 | 48.72 | 30.02 | 67.79 |
| 2012 | Guinea | Women | Middle | No | <25 | 555 | 83.27 | 79.38 | 86.54 |
| 2012 | Guinea | Women | Middle | No | 25+ | 103 | 16.73 | 13.46 | 20.62 |
| 2012 | Guinea | Women | Middle | Yes | <25 | 17 | 87.59 | 59.83 | 97.10 |
| 2012 | Guinea | Women | Middle | Yes | 25+ | 2 | 12.41 | 2.90 | 40.17 |
| 2012 | Guinea | Women | Poorer | No | <25 | 588 | 88.88 | 86.32 | 91.02 |
| 2012 | Guinea | Women | Poorer | No | 25+ | 71 | 11.12 | 8.98 | 13.68 |
| 2012 | Guinea | Women | Poorer | Yes | <25 | 5 | 100.00 | 100.00 | 100.00 |
| 2012 | Guinea | Women | Poorest | No | <25 | 619 | 93.93 | 91.14 | 95.88 |
| 2012 | Guinea | Women | Poorest | No | 25+ | 42 | 6.07 | 4.12 | 8.86 |
| 2012 | Guinea | Women | Poorest | Yes | <25 | 7 | 100.00 | 100.00 | 100.00 |
| 2012 | Guinea | Women | Richer | No | <25 | 599 | 71.77 | 67.76 | 75.47 |
| 2012 | Guinea | Women | Richer | No | 25+ | 236 | 28.23 | 24.53 | 32.24 |
| 2012 | Guinea | Women | Richer | Yes | <25 | 21 | 90.64 | 71.62 | 97.38 |
| 2012 | Guinea | Women | Richer | Yes | 25+ | 3 | 9.36 | 2.62 | 28.38 |
| 2012 | Guinea | Women | Richest | No | <25 | 422 | 60.24 | 55.87 | 64.46 |
| 2012 | Guinea | Women | Richest | No | 25+ | 261 | 39.76 | 35.54 | 44.13 |
| 2012 | Guinea | Women | Richest | Yes | <25 | 21 | 67.87 | 48.46 | 82.60 |
| 2012 | Guinea | Women | Richest | Yes | 25+ | 14 | 32.13 | 17.40 | 51.54 |
| 2018 | Guinea | Women | Middle | No | <25 | 549 | 75.72 | 71.27 | 79.67 |
| 2018 | Guinea | Women | Middle | No | 25+ | 179 | 24.28 | 20.33 | 28.73 |
| 2018 | Guinea | Women | Middle | Yes | <25 | 12 | 68.12 | 44.70 | 84.96 |
| 2018 | Guinea | Women | Middle | Yes | 25+ | 6 | 31.88 | 15.04 | 55.30 |
| 2018 | Guinea | Women | Poorer | No | <25 | 651 | 78.74 | 75.33 | 81.79 |
| 2018 | Guinea | Women | Poorer | No | 25+ | 169 | 21.26 | 18.21 | 24.67 |
| 2018 | Guinea | Women | Poorer | Yes | <25 | 7 | 76.29 | 38.91 | 94.21 |
| 2018 | Guinea | Women | Poorer | Yes | 25+ | 2 | 23.71 | 5.79 | 61.09 |
| 2018 | Guinea | Women | Poorest | No | <25 | 699 | 86.85 | 83.23 | 89.79 |
| 2018 | Guinea | Women | Poorest | No | 25+ | 112 | 13.15 | 10.21 | 16.77 |
| 2018 | Guinea | Women | Poorest | Yes | <25 | 9 | 100.00 | 100.00 | 100.00 |
| 2018 | Guinea | Women | Richer | No | <25 | 465 | 60.25 | 55.70 | 64.62 |
| 2018 | Guinea | Women | Richer | No | 25+ | 314 | 39.75 | 35.38 | 44.30 |
| 2018 | Guinea | Women | Richer | Yes | <25 | 15 | 68.76 | 43.14 | 86.46 |
| 2018 | Guinea | Women | Richer | Yes | 25+ | 6 | 31.24 | 13.54 | 56.86 |
| 2018 | Guinea | Women | Richest | No | <25 | 450 | 54.45 | 49.97 | 58.87 |
| 2018 | Guinea | Women | Richest | No | 25+ | 369 | 45.55 | 41.13 | 50.03 |
| 2018 | Guinea | Women | Richest | Yes | <25 | 8 | 40.60 | 20.17 | 64.90 |
| 2018 | Guinea | Women | Richest | Yes | 25+ | 11 | 59.40 | 35.10 | 79.83 |
| 2003 | Kenya | Women | Middle | No | <25 | 322 | 78.84 | 74.26 | 82.79 |
| 2003 | Kenya | Women | Middle | No | 25+ | 93 | 21.16 | 17.21 | 25.74 |
| 2003 | Kenya | Women | Middle | Yes | <25 | 29 | 83.87 | 68.18 | 92.66 |
| 2003 | Kenya | Women | Middle | Yes | 25+ | 6 | 16.13 | 7.34 | 31.82 |
| 2003 | Kenya | Women | Poorer | No | <25 | 351 | 85.23 | 80.58 | 88.91 |
| 2003 | Kenya | Women | Poorer | No | 25+ | 64 | 14.77 | 11.09 | 19.42 |
| 2003 | Kenya | Women | Poorer | Yes | <25 | 34 | 88.81 | 72.99 | 95.88 |
| 2003 | Kenya | Women | Poorer | Yes | 25+ | 4 | 11.19 | 4.12 | 27.01 |
| 2003 | Kenya | Women | Poorest | No | <25 | 344 | 88.96 | 84.27 | 92.38 |
| 2003 | Kenya | Women | Poorest | No | 25+ | 39 | 11.04 | 7.62 | 15.73 |
| 2003 | Kenya | Women | Poorest | Yes | <25 | 14 | 89.76 | 66.12 | 97.52 |
| 2003 | Kenya | Women | Poorest | Yes | 25+ | 2 | 10.24 | 2.48 | 33.88 |
| 2003 | Kenya | Women | Richer | No | <25 | 299 | 66.59 | 61.57 | 71.26 |
| 2003 | Kenya | Women | Richer | No | 25+ | 148 | 33.41 | 28.74 | 38.43 |
| 2003 | Kenya | Women | Richer | Yes | <25 | 44 | 81.08 | 67.28 | 89.93 |
| 2003 | Kenya | Women | Richer | Yes | 25+ | 12 | 18.92 | 10.07 | 32.72 |
| 2003 | Kenya | Women | Richest | No | <25 | 361 | 54.66 | 49.90 | 59.34 |
| 2003 | Kenya | Women | Richest | No | 25+ | 289 | 45.34 | 40.66 | 50.10 |
| 2003 | Kenya | Women | Richest | Yes | <25 | 66 | 64.77 | 49.80 | 77.31 |
| 2003 | Kenya | Women | Richest | Yes | 25+ | 30 | 35.23 | 22.69 | 50.20 |
| 2008 | Kenya | Women | Middle | No | <25 | 341 | 77.12 | 70.87 | 82.36 |
| 2008 | Kenya | Women | Middle | No | 25+ | 115 | 22.88 | 17.64 | 29.13 |
| 2008 | Kenya | Women | Middle | Yes | <25 | 33 | 73.01 | 48.94 | 88.42 |
| 2008 | Kenya | Women | Middle | Yes | 25+ | 7 | 26.99 | 11.58 | 51.06 |
| 2008 | Kenya | Women | Poorer | No | <25 | 357 | 86.39 | 81.28 | 90.28 |
| 2008 | Kenya | Women | Poorer | No | 25+ | 69 | 13.61 | 9.72 | 18.72 |
| 2008 | Kenya | Women | Poorer | Yes | <25 | 50 | 91.41 | 80.64 | 96.45 |
| 2008 | Kenya | Women | Poorer | Yes | 25+ | 6 | 8.59 | 3.55 | 19.36 |
| 2008 | Kenya | Women | Poorest | No | <25 | 492 | 87.98 | 83.59 | 91.33 |
| 2008 | Kenya | Women | Poorest | No | 25+ | 59 | 12.02 | 8.67 | 16.41 |
| 2008 | Kenya | Women | Poorest | Yes | <25 | 35 | 94.66 | 76.68 | 98.96 |
| 2008 | Kenya | Women | Poorest | Yes | 25+ | 2 | 5.34 | 1.04 | 23.32 |
| 2008 | Kenya | Women | Richer | No | <25 | 358 | 65.77 | 60.09 | 71.04 |
| 2008 | Kenya | Women | Richer | No | 25+ | 195 | 34.23 | 28.96 | 39.91 |
| 2008 | Kenya | Women | Richer | Yes | <25 | 38 | 56.14 | 35.51 | 74.85 |
| 2008 | Kenya | Women | Richer | Yes | 25+ | 13 | 43.86 | 25.15 | 64.49 |
| 2008 | Kenya | Women | Richest | No | <25 | 449 | 55.58 | 49.25 | 61.74 |
| 2008 | Kenya | Women | Richest | No | 25+ | 361 | 44.42 | 38.26 | 50.75 |
| 2008 | Kenya | Women | Richest | Yes | <25 | 58 | 54.66 | 35.19 | 72.80 |
| 2008 | Kenya | Women | Richest | Yes | 25+ | 39 | 45.34 | 27.20 | 64.81 |
| 2004 | Lesotho | Women | Middle | No | <25 | 167 | 57.29 | 49.79 | 64.46 |
| 2004 | Lesotho | Women | Middle | No | 25+ | 130 | 42.71 | 35.54 | 50.21 |
| 2004 | Lesotho | Women | Middle | Yes | <25 | 82 | 65.11 | 55.33 | 73.76 |
| 2004 | Lesotho | Women | Middle | Yes | 25+ | 43 | 34.89 | 26.24 | 44.67 |
| 2004 | Lesotho | Women | Poorer | No | <25 | 229 | 66.22 | 60.10 | 71.84 |
| 2004 | Lesotho | Women | Poorer | No | 25+ | 107 | 33.78 | 28.16 | 39.90 |
| 2004 | Lesotho | Women | Poorer | Yes | <25 | 99 | 64.80 | 55.66 | 72.96 |
| 2004 | Lesotho | Women | Poorer | Yes | 25+ | 54 | 35.20 | 27.04 | 44.34 |
| 2004 | Lesotho | Women | Poorest | No | <25 | 208 | 68.55 | 62.03 | 74.41 |
| 2004 | Lesotho | Women | Poorest | No | 25+ | 93 | 31.45 | 25.59 | 37.97 |
| 2004 | Lesotho | Women | Poorest | Yes | <25 | 62 | 71.46 | 60.14 | 80.59 |
| 2004 | Lesotho | Women | Poorest | Yes | 25+ | 26 | 28.54 | 19.41 | 39.86 |
| 2004 | Lesotho | Women | Richer | No | <25 | 166 | 49.66 | 43.06 | 56.27 |
| 2004 | Lesotho | Women | Richer | No | 25+ | 162 | 50.34 | 43.73 | 56.94 |
| 2004 | Lesotho | Women | Richer | Yes | <25 | 94 | 57.71 | 48.69 | 66.24 |
| 2004 | Lesotho | Women | Richer | Yes | 25+ | 71 | 42.29 | 33.76 | 51.31 |
| 2004 | Lesotho | Women | Richest | No | <25 | 146 | 37.70 | 31.93 | 43.85 |
| 2004 | Lesotho | Women | Richest | No | 25+ | 228 | 62.30 | 56.15 | 68.07 |
| 2004 | Lesotho | Women | Richest | Yes | <25 | 78 | 44.61 | 36.45 | 53.08 |
| 2004 | Lesotho | Women | Richest | Yes | 25+ | 107 | 55.39 | 46.92 | 63.55 |
| 2009 | Lesotho | Men | Middle | No | <25 | 379 | 90.92 | 87.30 | 93.59 |
| 2009 | Lesotho | Men | Middle | No | 25+ | 37 | 9.08 | 6.41 | 12.70 |
| 2009 | Lesotho | Men | Middle | Yes | <25 | 111 | 90.54 | 83.51 | 94.76 |
| 2009 | Lesotho | Men | Middle | Yes | 25+ | 10 | 9.46 | 5.24 | 16.49 |
| 2009 | Lesotho | Men | Poorer | No | <25 | 439 | 95.34 | 92.68 | 97.07 |
| 2009 | Lesotho | Men | Poorer | No | 25+ | 26 | 4.66 | 2.93 | 7.32 |
| 2009 | Lesotho | Men | Poorer | Yes | <25 | 112 | 95.39 | 86.80 | 98.49 |
| 2009 | Lesotho | Men | Poorer | Yes | 25+ | 4 | 4.61 | 1.51 | 13.20 |
| 2009 | Lesotho | Men | Poorest | No | <25 | 418 | 94.44 | 91.46 | 96.41 |
| 2009 | Lesotho | Men | Poorest | No | 25+ | 25 | 5.56 | 3.59 | 8.54 |
| 2009 | Lesotho | Men | Poorest | Yes | <25 | 90 | 95.24 | 89.20 | 97.98 |
| 2009 | Lesotho | Men | Poorest | Yes | 25+ | 5 | 4.76 | 2.02 | 10.80 |
| 2009 | Lesotho | Men | Richer | No | <25 | 308 | 85.44 | 80.00 | 89.60 |
| 2009 | Lesotho | Men | Richer | No | 25+ | 48 | 14.56 | 10.40 | 20.00 |
| 2009 | Lesotho | Men | Richer | Yes | <25 | 87 | 85.27 | 75.95 | 91.39 |
| 2009 | Lesotho | Men | Richer | Yes | 25+ | 14 | 14.73 | 8.61 | 24.05 |
| 2009 | Lesotho | Men | Richest | No | <25 | 269 | 74.21 | 69.08 | 78.75 |
| 2009 | Lesotho | Men | Richest | No | 25+ | 97 | 25.79 | 21.25 | 30.92 |
| 2009 | Lesotho | Men | Richest | Yes | <25 | 78 | 82.53 | 73.03 | 89.18 |
| 2009 | Lesotho | Men | Richest | Yes | 25+ | 18 | 17.47 | 10.82 | 26.97 |
| 2009 | Lesotho | Women | Middle | No | <25 | 205 | 50.91 | 44.49 | 57.30 |
| 2009 | Lesotho | Women | Middle | No | 25+ | 185 | 49.09 | 42.70 | 55.51 |
| 2009 | Lesotho | Women | Middle | Yes | <25 | 127 | 71.31 | 62.96 | 78.42 |
| 2009 | Lesotho | Women | Middle | Yes | 25+ | 65 | 28.69 | 21.58 | 37.04 |
| 2009 | Lesotho | Women | Poorer | No | <25 | 246 | 57.36 | 51.19 | 63.30 |
| 2009 | Lesotho | Women | Poorer | No | 25+ | 169 | 42.64 | 36.70 | 48.81 |
| 2009 | Lesotho | Women | Poorer | Yes | <25 | 107 | 62.50 | 54.22 | 70.11 |
| 2009 | Lesotho | Women | Poorer | Yes | 25+ | 72 | 37.50 | 29.89 | 45.78 |
| 2009 | Lesotho | Women | Poorest | No | <25 | 346 | 71.15 | 65.47 | 76.24 |
| 2009 | Lesotho | Women | Poorest | No | 25+ | 119 | 28.85 | 23.76 | 34.53 |
| 2009 | Lesotho | Women | Poorest | Yes | <25 | 106 | 73.00 | 64.23 | 80.29 |
| 2009 | Lesotho | Women | Poorest | Yes | 25+ | 36 | 27.00 | 19.71 | 35.77 |
| 2009 | Lesotho | Women | Richer | No | <25 | 199 | 50.24 | 43.77 | 56.70 |
| 2009 | Lesotho | Women | Richer | No | 25+ | 215 | 49.76 | 43.30 | 56.23 |
| 2009 | Lesotho | Women | Richer | Yes | <25 | 132 | 55.43 | 48.63 | 62.03 |
| 2009 | Lesotho | Women | Richer | Yes | 25+ | 102 | 44.57 | 37.97 | 51.37 |
| 2009 | Lesotho | Women | Richest | No | <25 | 177 | 36.55 | 32.18 | 41.15 |
| 2009 | Lesotho | Women | Richest | No | 25+ | 305 | 63.45 | 58.85 | 67.82 |
| 2009 | Lesotho | Women | Richest | Yes | <25 | 90 | 49.30 | 41.88 | 56.76 |
| 2009 | Lesotho | Women | Richest | Yes | 25+ | 98 | 50.70 | 43.24 | 58.12 |
| 2014 | Lesotho | Men | Middle | No | <25 | 326 | 86.65 | 82.53 | 89.92 |
| 2014 | Lesotho | Men | Middle | No | 25+ | 54 | 13.35 | 10.08 | 17.47 |
| 2014 | Lesotho | Men | Middle | Yes | <25 | 90 | 87.95 | 79.28 | 93.30 |
| 2014 | Lesotho | Men | Middle | Yes | 25+ | 14 | 12.05 | 6.70 | 20.72 |
| 2014 | Lesotho | Men | Poorer | No | <25 | 359 | 93.73 | 90.21 | 96.04 |
| 2014 | Lesotho | Men | Poorer | No | 25+ | 24 | 6.27 | 3.96 | 9.79 |
| 2014 | Lesotho | Men | Poorer | Yes | <25 | 80 | 95.25 | 85.95 | 98.50 |
| 2014 | Lesotho | Men | Poorer | Yes | 25+ | 4 | 4.75 | 1.50 | 14.05 |
| 2014 | Lesotho | Men | Poorest | No | <25 | 310 | 92.77 | 88.09 | 95.70 |
| 2014 | Lesotho | Men | Poorest | No | 25+ | 22 | 7.23 | 4.30 | 11.91 |
| 2014 | Lesotho | Men | Poorest | Yes | <25 | 80 | 89.65 | 79.36 | 95.12 |
| 2014 | Lesotho | Men | Poorest | Yes | 25+ | 9 | 10.35 | 4.88 | 20.64 |
| 2014 | Lesotho | Men | Richer | No | <25 | 303 | 85.20 | 80.24 | 89.09 |
| 2014 | Lesotho | Men | Richer | No | 25+ | 57 | 14.80 | 10.91 | 19.76 |
| 2014 | Lesotho | Men | Richer | Yes | <25 | 103 | 92.48 | 86.03 | 96.09 |
| 2014 | Lesotho | Men | Richer | Yes | 25+ | 11 | 7.52 | 3.91 | 13.97 |
| 2014 | Lesotho | Men | Richest | No | <25 | 271 | 70.66 | 64.95 | 75.78 |
| 2014 | Lesotho | Men | Richest | No | 25+ | 121 | 29.34 | 24.22 | 35.05 |
| 2014 | Lesotho | Men | Richest | Yes | <25 | 84 | 76.84 | 66.18 | 84.91 |
| 2014 | Lesotho | Men | Richest | Yes | 25+ | 30 | 23.16 | 15.09 | 33.82 |
| 2014 | Lesotho | Women | Middle | No | <25 | 163 | 48.08 | 41.73 | 54.49 |
| 2014 | Lesotho | Women | Middle | No | 25+ | 174 | 51.92 | 45.51 | 58.27 |
| 2014 | Lesotho | Women | Middle | Yes | <25 | 93 | 55.20 | 46.01 | 64.05 |
| 2014 | Lesotho | Women | Middle | Yes | 25+ | 83 | 44.80 | 35.95 | 53.99 |
| 2014 | Lesotho | Women | Poorer | No | <25 | 200 | 55.32 | 49.36 | 61.14 |
| 2014 | Lesotho | Women | Poorer | No | 25+ | 155 | 44.68 | 38.86 | 50.64 |
| 2014 | Lesotho | Women | Poorer | Yes | <25 | 91 | 62.52 | 52.95 | 71.20 |
| 2014 | Lesotho | Women | Poorer | Yes | 25+ | 53 | 37.48 | 28.80 | 47.05 |
| 2014 | Lesotho | Women | Poorest | No | <25 | 240 | 71.94 | 66.40 | 76.88 |
| 2014 | Lesotho | Women | Poorest | No | 25+ | 97 | 28.06 | 23.12 | 33.60 |
| 2014 | Lesotho | Women | Poorest | Yes | <25 | 98 | 71.57 | 62.75 | 78.99 |
| 2014 | Lesotho | Women | Poorest | Yes | 25+ | 39 | 28.43 | 21.01 | 37.25 |
| 2014 | Lesotho | Women | Richer | No | <25 | 144 | 38.56 | 33.04 | 44.40 |
| 2014 | Lesotho | Women | Richer | No | 25+ | 221 | 61.44 | 55.60 | 66.96 |
| 2014 | Lesotho | Women | Richer | Yes | <25 | 111 | 54.43 | 46.85 | 61.82 |
| 2014 | Lesotho | Women | Richer | Yes | 25+ | 102 | 45.57 | 38.18 | 53.15 |
| 2014 | Lesotho | Women | Richest | No | <25 | 165 | 40.72 | 35.42 | 46.25 |
| 2014 | Lesotho | Women | Richest | No | 25+ | 268 | 59.28 | 53.75 | 64.58 |
| 2014 | Lesotho | Women | Richest | Yes | <25 | 86 | 42.37 | 34.94 | 50.18 |
| 2014 | Lesotho | Women | Richest | Yes | 25+ | 117 | 57.63 | 49.82 | 65.06 |
| 2006 | Mali | Women | Middle | No | <25 | 575 | 86.46 | 82.67 | 89.52 |
| 2006 | Mali | Women | Middle | No | 25+ | 103 | 13.54 | 10.48 | 17.33 |
| 2006 | Mali | Women | Middle | Yes | <25 | 5 | 100.00 | 100.00 | 100.00 |
| 2006 | Mali | Women | Poorer | No | <25 | 583 | 88.84 | 85.56 | 91.44 |
| 2006 | Mali | Women | Poorer | No | 25+ | 76 | 11.16 | 8.56 | 14.44 |
| 2006 | Mali | Women | Poorer | Yes | <25 | 5 | 78.71 | 31.99 | 96.67 |
| 2006 | Mali | Women | Poorer | Yes | 25+ | 2 | 21.29 | 3.33 | 68.01 |
| 2006 | Mali | Women | Poorest | No | <25 | 523 | 92.76 | 89.83 | 94.89 |
| 2006 | Mali | Women | Poorest | No | 25+ | 45 | 7.24 | 5.11 | 10.17 |
| 2006 | Mali | Women | Poorest | Yes | <25 | 9 | 76.08 | 36.75 | 94.57 |
| 2006 | Mali | Women | Poorest | Yes | 25+ | 2 | 23.92 | 5.43 | 63.25 |
| 2006 | Mali | Women | Richer | No | <25 | 584 | 73.02 | 63.30 | 80.94 |
| 2006 | Mali | Women | Richer | No | 25+ | 183 | 26.98 | 19.06 | 36.70 |
| 2006 | Mali | Women | Richer | Yes | <25 | 15 | 89.75 | 55.96 | 98.37 |
| 2006 | Mali | Women | Richer | Yes | 25+ | 2 | 10.25 | 1.63 | 44.04 |
| 2006 | Mali | Women | Richest | No | <25 | 461 | 63.19 | 59.56 | 66.67 |
| 2006 | Mali | Women | Richest | No | 25+ | 303 | 36.81 | 33.33 | 40.44 |
| 2006 | Mali | Women | Richest | Yes | <25 | 12 | 70.23 | 36.68 | 90.57 |
| 2006 | Mali | Women | Richest | Yes | 25+ | 4 | 29.77 | 9.43 | 63.32 |
| 2012 | Mali | Women | Middle | No | <25 | 627 | 88.70 | 85.78 | 91.08 |
| 2012 | Mali | Women | Middle | No | 25+ | 84 | 11.30 | 8.92 | 14.22 |
| 2012 | Mali | Women | Middle | Yes | <25 | 9 | 94.97 | 69.91 | 99.35 |
| 2012 | Mali | Women | Middle | Yes | 25+ | 1 | 5.03 | 0.65 | 30.09 |
| 2012 | Mali | Women | Poorer | No | <25 | 627 | 88.53 | 85.47 | 91.01 |
| 2012 | Mali | Women | Poorer | No | 25+ | 90 | 11.47 | 8.99 | 14.53 |
| 2012 | Mali | Women | Poorer | Yes | <25 | 1 | 100.00 | 100.00 | 100.00 |
| 2012 | Mali | Women | Poorest | No | <25 | 659 | 88.22 | 85.27 | 90.64 |
| 2012 | Mali | Women | Poorest | No | 25+ | 90 | 11.78 | 9.36 | 14.73 |
| 2012 | Mali | Women | Poorest | Yes | <25 | 6 | 72.71 | 23.82 | 95.78 |
| 2012 | Mali | Women | Poorest | Yes | 25+ | 1 | 27.29 | 4.22 | 76.18 |
| 2012 | Mali | Women | Richer | No | <25 | 589 | 79.51 | 76.22 | 82.45 |
| 2012 | Mali | Women | Richer | No | 25+ | 160 | 20.49 | 17.55 | 23.78 |
| 2012 | Mali | Women | Richer | Yes | <25 | 8 | 79.01 | 42.59 | 95.03 |
| 2012 | Mali | Women | Richer | Yes | 25+ | 2 | 20.99 | 4.97 | 57.41 |
| 2012 | Mali | Women | Richest | No | <25 | 635 | 63.20 | 59.79 | 66.49 |
| 2012 | Mali | Women | Richest | No | 25+ | 405 | 36.80 | 33.51 | 40.21 |
| 2012 | Mali | Women | Richest | Yes | <25 | 13 | 53.52 | 30.90 | 74.78 |
| 2012 | Mali | Women | Richest | Yes | 25+ | 9 | 46.48 | 25.22 | 69.10 |
| 2004 | Malawi | Women | Middle | No | <25 | 372 | 90.18 | 86.10 | 93.16 |
| 2004 | Malawi | Women | Middle | No | 25+ | 38 | 9.82 | 6.84 | 13.90 |
| 2004 | Malawi | Women | Middle | Yes | <25 | 76 | 95.50 | 89.05 | 98.23 |
| 2004 | Malawi | Women | Middle | Yes | 25+ | 5 | 4.50 | 1.77 | 10.95 |
| 2004 | Malawi | Women | Poorer | No | <25 | 358 | 90.77 | 87.20 | 93.41 |
| 2004 | Malawi | Women | Poorer | No | 25+ | 41 | 9.23 | 6.59 | 12.80 |
| 2004 | Malawi | Women | Poorer | Yes | <25 | 53 | 92.95 | 78.59 | 97.93 |
| 2004 | Malawi | Women | Poorer | Yes | 25+ | 4 | 7.05 | 2.07 | 21.41 |
| 2004 | Malawi | Women | Poorest | No | <25 | 309 | 89.95 | 84.75 | 93.51 |
| 2004 | Malawi | Women | Poorest | No | 25+ | 28 | 10.05 | 6.49 | 15.25 |
| 2004 | Malawi | Women | Poorest | Yes | <25 | 50 | 88.98 | 76.12 | 95.34 |
| 2004 | Malawi | Women | Poorest | Yes | 25+ | 6 | 11.02 | 4.66 | 23.88 |
| 2004 | Malawi | Women | Richer | No | <25 | 314 | 84.02 | 79.50 | 87.70 |
| 2004 | Malawi | Women | Richer | No | 25+ | 60 | 15.98 | 12.30 | 20.50 |
| 2004 | Malawi | Women | Richer | Yes | <25 | 74 | 84.19 | 72.31 | 91.57 |
| 2004 | Malawi | Women | Richer | Yes | 25+ | 13 | 15.81 | 8.43 | 27.69 |
| 2004 | Malawi | Women | Richest | No | <25 | 211 | 69.69 | 62.97 | 75.66 |
| 2004 | Malawi | Women | Richest | No | 25+ | 88 | 30.31 | 24.34 | 37.03 |
| 2004 | Malawi | Women | Richest | Yes | <25 | 65 | 72.50 | 60.12 | 82.18 |
| 2004 | Malawi | Women | Richest | Yes | 25+ | 25 | 27.50 | 17.82 | 39.88 |
| 2010 | Malawi | Women | Middle | No | <25 | 837 | 84.10 | 80.73 | 86.98 |
| 2010 | Malawi | Women | Middle | No | 25+ | 140 | 15.90 | 13.02 | 19.27 |
| 2010 | Malawi | Women | Middle | Yes | <25 | 126 | 92.09 | 83.44 | 96.42 |
| 2010 | Malawi | Women | Middle | Yes | 25+ | 11 | 7.91 | 3.58 | 16.56 |
| 2010 | Malawi | Women | Poorer | No | <25 | 898 | 86.73 | 83.65 | 89.30 |
| 2010 | Malawi | Women | Poorer | No | 25+ | 122 | 13.27 | 10.70 | 16.35 |
| 2010 | Malawi | Women | Poorer | Yes | <25 | 112 | 84.48 | 76.35 | 90.17 |
| 2010 | Malawi | Women | Poorer | Yes | 25+ | 20 | 15.52 | 9.83 | 23.65 |
| 2010 | Malawi | Women | Poorest | No | <25 | 819 | 89.59 | 86.87 | 91.79 |
| 2010 | Malawi | Women | Poorest | No | 25+ | 88 | 10.41 | 8.21 | 13.13 |
| 2010 | Malawi | Women | Poorest | Yes | <25 | 102 | 92.62 | 84.70 | 96.60 |
| 2010 | Malawi | Women | Poorest | Yes | 25+ | 8 | 7.38 | 3.40 | 15.30 |
| 2010 | Malawi | Women | Richer | No | <25 | 804 | 81.65 | 78.68 | 84.30 |
| 2010 | Malawi | Women | Richer | No | 25+ | 197 | 18.35 | 15.70 | 21.32 |
| 2010 | Malawi | Women | Richer | Yes | <25 | 137 | 79.70 | 71.98 | 85.72 |
| 2010 | Malawi | Women | Richer | Yes | 25+ | 36 | 20.30 | 14.28 | 28.02 |
| 2010 | Malawi | Women | Richest | No | <25 | 623 | 68.47 | 64.54 | 72.14 |
| 2010 | Malawi | Women | Richest | No | 25+ | 273 | 31.53 | 27.86 | 35.46 |
| 2010 | Malawi | Women | Richest | Yes | <25 | 165 | 67.06 | 59.63 | 73.73 |
| 2010 | Malawi | Women | Richest | Yes | 25+ | 79 | 32.94 | 26.27 | 40.37 |
| 2015 | Malawi | Women | Middle | No | <25 | 819 | 82.32 | 79.24 | 85.03 |
| 2015 | Malawi | Women | Middle | No | 25+ | 189 | 17.68 | 14.97 | 20.76 |
| 2015 | Malawi | Women | Middle | Yes | <25 | 107 | 75.20 | 64.59 | 83.45 |
| 2015 | Malawi | Women | Middle | Yes | 25+ | 28 | 24.80 | 16.55 | 35.41 |
| 2015 | Malawi | Women | Poorer | No | <25 | 890 | 86.39 | 83.73 | 88.68 |
| 2015 | Malawi | Women | Poorer | No | 25+ | 144 | 13.61 | 11.32 | 16.27 |
| 2015 | Malawi | Women | Poorer | Yes | <25 | 98 | 87.68 | 76.24 | 94.04 |
| 2015 | Malawi | Women | Poorer | Yes | 25+ | 14 | 12.32 | 5.96 | 23.76 |
| 2015 | Malawi | Women | Poorest | No | <25 | 816 | 87.21 | 83.63 | 90.09 |
| 2015 | Malawi | Women | Poorest | No | 25+ | 102 | 12.79 | 9.91 | 16.37 |
| 2015 | Malawi | Women | Poorest | Yes | <25 | 105 | 85.15 | 76.68 | 90.90 |
| 2015 | Malawi | Women | Poorest | Yes | 25+ | 18 | 14.85 | 9.10 | 23.32 |
| 2015 | Malawi | Women | Richer | No | <25 | 794 | 75.12 | 71.79 | 78.17 |
| 2015 | Malawi | Women | Richer | No | 25+ | 287 | 24.88 | 21.83 | 28.21 |
| 2015 | Malawi | Women | Richer | Yes | <25 | 117 | 70.67 | 61.04 | 78.75 |
| 2015 | Malawi | Women | Richer | Yes | 25+ | 40 | 29.33 | 21.25 | 38.96 |
| 2015 | Malawi | Women | Richest | No | <25 | 782 | 59.54 | 56.19 | 62.81 |
| 2015 | Malawi | Women | Richest | No | 25+ | 561 | 40.46 | 37.19 | 43.81 |
| 2015 | Malawi | Women | Richest | Yes | <25 | 148 | 56.92 | 48.63 | 64.83 |
| 2015 | Malawi | Women | Richest | Yes | 25+ | 108 | 43.08 | 35.17 | 51.37 |
| 2006 | Niger | Women | Middle | No | <25 | 517 | 91.99 | 89.41 | 93.99 |
| 2006 | Niger | Women | Middle | No | 25+ | 53 | 8.01 | 6.01 | 10.59 |
| 2006 | Niger | Women | Middle | Yes | <25 | 1 | 100.00 | 100.00 | 100.00 |
| 2006 | Niger | Women | Poorer | No | <25 | 484 | 94.58 | 92.17 | 96.28 |
| 2006 | Niger | Women | Poorer | No | 25+ | 31 | 5.42 | 3.72 | 7.83 |
| 2006 | Niger | Women | Poorer | Yes | <25 | 3 | 100.00 | 100.00 | 100.00 |
| 2006 | Niger | Women | Poorest | No | <25 | 496 | 95.42 | 92.99 | 97.04 |
| 2006 | Niger | Women | Poorest | No | 25+ | 28 | 4.58 | 2.96 | 7.01 |
| 2006 | Niger | Women | Poorest | Yes | <25 | 3 | 100.00 | 100.00 | 100.00 |
| 2006 | Niger | Women | Richer | No | <25 | 506 | 87.52 | 84.06 | 90.31 |
| 2006 | Niger | Women | Richer | No | 25+ | 102 | 12.48 | 9.69 | 15.94 |
| 2006 | Niger | Women | Richer | Yes | <25 | 6 | 74.98 | 45.14 | 91.60 |
| 2006 | Niger | Women | Richer | Yes | 25+ | 3 | 25.02 | 8.40 | 54.86 |
| 2006 | Niger | Women | Richest | No | <25 | 627 | 62.77 | 58.74 | 66.62 |
| 2006 | Niger | Women | Richest | No | 25+ | 419 | 37.23 | 33.38 | 41.26 |
| 2006 | Niger | Women | Richest | Yes | <25 | 10 | 62.65 | 30.44 | 86.54 |
| 2006 | Niger | Women | Richest | Yes | 25+ | 8 | 37.35 | 13.46 | 69.56 |
| 2012 | Niger | Women | Middle | No | <25 | 587 | 88.28 | 85.07 | 90.88 |
| 2012 | Niger | Women | Middle | No | 25+ | 85 | 11.72 | 9.12 | 14.93 |
| 2012 | Niger | Women | Middle | Yes | <25 | 1 | 100.00 | 100.00 | 100.00 |
| 2012 | Niger | Women | Poorer | No | <25 | 549 | 87.29 | 83.84 | 90.08 |
| 2012 | Niger | Women | Poorer | No | 25+ | 85 | 12.71 | 9.92 | 16.16 |
| 2012 | Niger | Women | Poorer | Yes | <25 | 2 | 100.00 | 100.00 | 100.00 |
| 2012 | Niger | Women | Poorest | No | <25 | 556 | 87.43 | 83.49 | 90.53 |
| 2012 | Niger | Women | Poorest | No | 25+ | 76 | 12.57 | 9.47 | 16.51 |
| 2012 | Niger | Women | Poorest | Yes | <25 | 2 | 100.00 | 100.00 | 100.00 |
| 2012 | Niger | Women | Richer | No | <25 | 617 | 83.26 | 79.55 | 86.40 |
| 2012 | Niger | Women | Richer | No | 25+ | 144 | 16.74 | 13.60 | 20.45 |
| 2012 | Niger | Women | Richer | Yes | <25 | 3 | 63.50 | 21.84 | 91.55 |
| 2012 | Niger | Women | Richer | Yes | 25+ | 3 | 36.50 | 8.45 | 78.16 |
| 2012 | Niger | Women | Richest | No | <25 | 661 | 58.66 | 55.21 | 62.02 |
| 2012 | Niger | Women | Richest | No | 25+ | 485 | 41.34 | 37.98 | 44.79 |
| 2012 | Niger | Women | Richest | Yes | <25 | 9 | 60.85 | 30.96 | 84.34 |
| 2012 | Niger | Women | Richest | Yes | 25+ | 4 | 39.15 | 15.66 | 69.04 |
| 2008 | Sierra Leone | Women | Middle | No | <25 | 358 | 73.78 | 67.97 | 78.87 |
| 2008 | Sierra Leone | Women | Middle | No | 25+ | 147 | 26.22 | 21.13 | 32.03 |
| 2008 | Sierra Leone | Women | Middle | Yes | <25 | 7 | 100.00 | 100.00 | 100.00 |
| 2008 | Sierra Leone | Women | Poorer | No | <25 | 375 | 79.44 | 74.04 | 83.96 |
| 2008 | Sierra Leone | Women | Poorer | No | 25+ | 115 | 20.56 | 16.04 | 25.96 |
| 2008 | Sierra Leone | Women | Poorer | Yes | <25 | 6 | 56.09 | 26.93 | 81.57 |
| 2008 | Sierra Leone | Women | Poorer | Yes | 25+ | 6 | 43.91 | 18.43 | 73.07 |
| 2008 | Sierra Leone | Women | Poorest | No | <25 | 371 | 73.84 | 66.96 | 79.72 |
| 2008 | Sierra Leone | Women | Poorest | No | 25+ | 156 | 26.16 | 20.28 | 33.04 |
| 2008 | Sierra Leone | Women | Poorest | Yes | <25 | 3 | 100.00 | 100.00 | 100.00 |
| 2008 | Sierra Leone | Women | Richer | No | <25 | 412 | 68.08 | 59.77 | 75.38 |
| 2008 | Sierra Leone | Women | Richer | No | 25+ | 177 | 31.92 | 24.62 | 40.23 |
| 2008 | Sierra Leone | Women | Richer | Yes | <25 | 10 | 74.54 | 45.11 | 91.25 |
| 2008 | Sierra Leone | Women | Richer | Yes | 25+ | 5 | 25.46 | 8.75 | 54.89 |
| 2008 | Sierra Leone | Women | Richest | No | <25 | 381 | 56.03 | 50.09 | 61.80 |
| 2008 | Sierra Leone | Women | Richest | No | 25+ | 281 | 43.97 | 38.20 | 49.91 |
| 2008 | Sierra Leone | Women | Richest | Yes | <25 | 14 | 64.97 | 41.71 | 82.78 |
| 2008 | Sierra Leone | Women | Richest | Yes | 25+ | 6 | 35.03 | 17.22 | 58.29 |
| 2013 | Sierra Leone | Men | Middle | No | <25 | 940 | 91.77 | 88.81 | 94.00 |
| 2013 | Sierra Leone | Men | Middle | No | 25+ | 90 | 8.23 | 6.00 | 11.19 |
| 2013 | Sierra Leone | Men | Middle | Yes | <25 | 8 | 100.00 | 100.00 | 100.00 |
| 2013 | Sierra Leone | Men | Poorer | No | <25 | 955 | 92.79 | 89.85 | 94.93 |
| 2013 | Sierra Leone | Men | Poorer | No | 25+ | 74 | 7.21 | 5.07 | 10.15 |
| 2013 | Sierra Leone | Men | Poorer | Yes | <25 | 8 | 100.00 | 100.00 | 100.00 |
| 2013 | Sierra Leone | Men | Poorest | No | <25 | 1018 | 93.49 | 90.92 | 95.37 |
| 2013 | Sierra Leone | Men | Poorest | No | 25+ | 70 | 6.51 | 4.63 | 9.08 |
| 2013 | Sierra Leone | Men | Poorest | Yes | <25 | 9 | 100.00 | 100.00 | 100.00 |
| 2013 | Sierra Leone | Men | Richer | No | <25 | 1108 | 91.52 | 89.31 | 93.30 |
| 2013 | Sierra Leone | Men | Richer | No | 25+ | 121 | 8.48 | 6.70 | 10.69 |
| 2013 | Sierra Leone | Men | Richer | Yes | <25 | 14 | 87.69 | 64.58 | 96.53 |
| 2013 | Sierra Leone | Men | Richer | Yes | 25+ | 3 | 12.31 | 3.47 | 35.42 |
| 2013 | Sierra Leone | Men | Richest | No | <25 | 1140 | 87.46 | 84.00 | 90.26 |
| 2013 | Sierra Leone | Men | Richest | No | 25+ | 202 | 12.54 | 9.74 | 16.00 |
| 2013 | Sierra Leone | Men | Richest | Yes | <25 | 23 | 76.70 | 56.61 | 89.25 |
| 2013 | Sierra Leone | Men | Richest | Yes | 25+ | 7 | 23.30 | 10.75 | 43.39 |
| 2013 | Sierra Leone | Women | Middle | No | <25 | 927 | 84.67 | 81.65 | 87.26 |
| 2013 | Sierra Leone | Women | Middle | No | 25+ | 181 | 15.33 | 12.74 | 18.35 |
| 2013 | Sierra Leone | Women | Middle | Yes | <25 | 12 | 82.76 | 46.97 | 96.30 |
| 2013 | Sierra Leone | Women | Middle | Yes | 25+ | 2 | 17.24 | 3.70 | 53.03 |
| 2013 | Sierra Leone | Women | Poorer | No | <25 | 868 | 85.17 | 82.05 | 87.84 |
| 2013 | Sierra Leone | Women | Poorer | No | 25+ | 161 | 14.83 | 12.16 | 17.95 |
| 2013 | Sierra Leone | Women | Poorer | Yes | <25 | 13 | 100.00 | 100.00 | 100.00 |
| 2013 | Sierra Leone | Women | Poorest | No | <25 | 991 | 88.65 | 86.17 | 90.73 |
| 2013 | Sierra Leone | Women | Poorest | No | 25+ | 131 | 11.35 | 9.27 | 13.83 |
| 2013 | Sierra Leone | Women | Poorest | Yes | <25 | 10 | 96.83 | 78.87 | 99.60 |
| 2013 | Sierra Leone | Women | Poorest | Yes | 25+ | 1 | 3.17 | 0.40 | 21.13 |
| 2013 | Sierra Leone | Women | Richer | No | <25 | 1077 | 77.09 | 73.40 | 80.41 |
| 2013 | Sierra Leone | Women | Richer | No | 25+ | 330 | 22.91 | 19.59 | 26.60 |
| 2013 | Sierra Leone | Women | Richer | Yes | <25 | 25 | 63.72 | 42.53 | 80.65 |
| 2013 | Sierra Leone | Women | Richer | Yes | 25+ | 11 | 36.28 | 19.35 | 57.47 |
| 2013 | Sierra Leone | Women | Richest | No | <25 | 879 | 66.82 | 63.11 | 70.34 |
| 2013 | Sierra Leone | Women | Richest | No | 25+ | 450 | 33.18 | 29.66 | 36.89 |
| 2013 | Sierra Leone | Women | Richest | Yes | <25 | 32 | 64.22 | 44.08 | 80.34 |
| 2013 | Sierra Leone | Women | Richest | Yes | 25+ | 14 | 35.78 | 19.66 | 55.92 |
| 2019 | Sierra Leone | Men | Middle | No | <25 | 893 | 91.59 | 89.53 | 93.28 |
| 2019 | Sierra Leone | Men | Middle | No | 25+ | 85 | 8.41 | 6.72 | 10.47 |
| 2019 | Sierra Leone | Men | Middle | Yes | <25 | 14 | 100.00 | 100.00 | 100.00 |
| 2019 | Sierra Leone | Men | Poorer | No | <25 | 930 | 94.10 | 92.07 | 95.63 |
| 2019 | Sierra Leone | Men | Poorer | No | 25+ | 59 | 5.90 | 4.37 | 7.93 |
| 2019 | Sierra Leone | Men | Poorer | Yes | <25 | 7 | 76.54 | 36.04 | 94.97 |
| 2019 | Sierra Leone | Men | Poorer | Yes | 25+ | 2 | 23.46 | 5.03 | 63.96 |
| 2019 | Sierra Leone | Men | Poorest | No | <25 | 1056 | 94.22 | 92.29 | 95.69 |
| 2019 | Sierra Leone | Men | Poorest | No | 25+ | 73 | 5.78 | 4.31 | 7.71 |
| 2019 | Sierra Leone | Men | Poorest | Yes | <25 | 6 | 95.98 | 70.96 | 99.57 |
| 2019 | Sierra Leone | Men | Poorest | Yes | 25+ | 1 | 4.02 | 0.43 | 29.04 |
| 2019 | Sierra Leone | Men | Richer | No | <25 | 916 | 87.13 | 84.57 | 89.32 |
| 2019 | Sierra Leone | Men | Richer | No | 25+ | 138 | 12.87 | 10.68 | 15.43 |
| 2019 | Sierra Leone | Men | Richer | Yes | <25 | 14 | 100.00 | 100.00 | 100.00 |
| 2019 | Sierra Leone | Men | Richest | No | <25 | 753 | 78.52 | 74.51 | 82.05 |
| 2019 | Sierra Leone | Men | Richest | No | 25+ | 207 | 21.48 | 17.95 | 25.49 |
| 2019 | Sierra Leone | Men | Richest | Yes | <25 | 17 | 80.35 | 59.32 | 91.97 |
| 2019 | Sierra Leone | Men | Richest | Yes | 25+ | 6 | 19.65 | 8.03 | 40.68 |
| 2019 | Sierra Leone | Women | Middle | No | <25 | 855 | 72.99 | 69.36 | 76.33 |
| 2019 | Sierra Leone | Women | Middle | No | 25+ | 292 | 27.01 | 23.67 | 30.64 |
| 2019 | Sierra Leone | Women | Middle | Yes | <25 | 17 | 71.34 | 43.91 | 88.79 |
| 2019 | Sierra Leone | Women | Middle | Yes | 25+ | 5 | 28.66 | 11.21 | 56.09 |
| 2019 | Sierra Leone | Women | Poorer | No | <25 | 878 | 81.04 | 78.03 | 83.73 |
| 2019 | Sierra Leone | Women | Poorer | No | 25+ | 208 | 18.96 | 16.27 | 21.97 |
| 2019 | Sierra Leone | Women | Poorer | Yes | <25 | 17 | 87.56 | 60.10 | 97.05 |
| 2019 | Sierra Leone | Women | Poorer | Yes | 25+ | 2 | 12.44 | 2.95 | 39.90 |
| 2019 | Sierra Leone | Women | Poorest | No | <25 | 963 | 81.39 | 78.13 | 84.26 |
| 2019 | Sierra Leone | Women | Poorest | No | 25+ | 222 | 18.61 | 15.74 | 21.87 |
| 2019 | Sierra Leone | Women | Poorest | Yes | <25 | 11 | 81.25 | 48.69 | 95.19 |
| 2019 | Sierra Leone | Women | Poorest | Yes | 25+ | 3 | 18.75 | 4.81 | 51.31 |
| 2019 | Sierra Leone | Women | Richer | No | <25 | 738 | 63.30 | 59.88 | 66.59 |
| 2019 | Sierra Leone | Women | Richer | No | 25+ | 461 | 36.70 | 33.41 | 40.12 |
| 2019 | Sierra Leone | Women | Richer | Yes | <25 | 27 | 69.36 | 50.78 | 83.24 |
| 2019 | Sierra Leone | Women | Richer | Yes | 25+ | 12 | 30.64 | 16.76 | 49.22 |
| 2019 | Sierra Leone | Women | Richest | No | <25 | 559 | 53.23 | 49.91 | 56.53 |
| 2019 | Sierra Leone | Women | Richest | No | 25+ | 479 | 46.77 | 43.47 | 50.09 |
| 2019 | Sierra Leone | Women | Richest | Yes | <25 | 27 | 66.95 | 49.28 | 80.85 |
| 2019 | Sierra Leone | Women | Richest | Yes | 25+ | 19 | 33.05 | 19.15 | 50.72 |
| 2005 | Zimbabwe | Women | Middle | No | <25 | 608 | 76.03 | 72.53 | 79.21 |
| 2005 | Zimbabwe | Women | Middle | No | 25+ | 198 | 23.97 | 20.79 | 27.47 |
| 2005 | Zimbabwe | Women | Middle | Yes | <25 | 237 | 87.85 | 82.47 | 91.74 |
| 2005 | Zimbabwe | Women | Middle | Yes | 25+ | 38 | 12.15 | 8.26 | 17.53 |
| 2005 | Zimbabwe | Women | Poorer | No | <25 | 682 | 81.95 | 78.78 | 84.73 |
| 2005 | Zimbabwe | Women | Poorer | No | 25+ | 168 | 18.05 | 15.27 | 21.22 |
| 2005 | Zimbabwe | Women | Poorer | Yes | <25 | 227 | 84.85 | 79.59 | 88.94 |
| 2005 | Zimbabwe | Women | Poorer | Yes | 25+ | 42 | 15.15 | 11.06 | 20.41 |
| 2005 | Zimbabwe | Women | Poorest | No | <25 | 738 | 85.85 | 82.27 | 88.80 |
| 2005 | Zimbabwe | Women | Poorest | No | 25+ | 140 | 14.15 | 11.20 | 17.73 |
| 2005 | Zimbabwe | Women | Poorest | Yes | <25 | 215 | 89.01 | 83.86 | 92.66 |
| 2005 | Zimbabwe | Women | Poorest | Yes | 25+ | 27 | 10.99 | 7.34 | 16.14 |
| 2005 | Zimbabwe | Women | Richer | No | <25 | 571 | 64.24 | 60.34 | 67.97 |
| 2005 | Zimbabwe | Women | Richer | No | 25+ | 313 | 35.76 | 32.03 | 39.66 |
| 2005 | Zimbabwe | Women | Richer | Yes | <25 | 261 | 73.77 | 68.71 | 78.27 |
| 2005 | Zimbabwe | Women | Richer | Yes | 25+ | 103 | 26.23 | 21.73 | 31.29 |
| 2005 | Zimbabwe | Women | Richest | No | <25 | 620 | 57.80 | 55.04 | 60.50 |
| 2005 | Zimbabwe | Women | Richest | No | 25+ | 460 | 42.20 | 39.50 | 44.96 |
| 2005 | Zimbabwe | Women | Richest | Yes | <25 | 155 | 59.55 | 52.89 | 65.88 |
| 2005 | Zimbabwe | Women | Richest | Yes | 25+ | 105 | 40.45 | 34.12 | 47.11 |
| 2010 | Zimbabwe | Men | Middle | No | <25 | 788 | 94.07 | 92.08 | 95.59 |
| 2010 | Zimbabwe | Men | Middle | No | 25+ | 50 | 5.93 | 4.41 | 7.92 |
| 2010 | Zimbabwe | Men | Middle | Yes | <25 | 144 | 95.76 | 91.05 | 98.05 |
| 2010 | Zimbabwe | Men | Middle | Yes | 25+ | 7 | 4.24 | 1.95 | 8.95 |
| 2010 | Zimbabwe | Men | Poorer | No | <25 | 732 | 94.83 | 92.75 | 96.33 |
| 2010 | Zimbabwe | Men | Poorer | No | 25+ | 39 | 5.17 | 3.67 | 7.25 |
| 2010 | Zimbabwe | Men | Poorer | Yes | <25 | 144 | 95.98 | 91.19 | 98.22 |
| 2010 | Zimbabwe | Men | Poorer | Yes | 25+ | 6 | 4.02 | 1.78 | 8.81 |
| 2010 | Zimbabwe | Men | Poorest | No | <25 | 763 | 97.31 | 95.69 | 98.33 |
| 2010 | Zimbabwe | Men | Poorest | No | 25+ | 24 | 2.69 | 1.67 | 4.31 |
| 2010 | Zimbabwe | Men | Poorest | Yes | <25 | 174 | 98.48 | 96.12 | 99.41 |
| 2010 | Zimbabwe | Men | Poorest | Yes | 25+ | 5 | 1.52 | 0.59 | 3.88 |
| 2010 | Zimbabwe | Men | Richer | No | <25 | 858 | 88.04 | 85.19 | 90.40 |
| 2010 | Zimbabwe | Men | Richer | No | 25+ | 110 | 11.96 | 9.60 | 14.81 |
| 2010 | Zimbabwe | Men | Richer | Yes | <25 | 145 | 88.30 | 81.64 | 92.76 |
| 2010 | Zimbabwe | Men | Richer | Yes | 25+ | 20 | 11.70 | 7.24 | 18.36 |
| 2010 | Zimbabwe | Men | Richest | No | <25 | 769 | 78.96 | 75.06 | 82.39 |
| 2010 | Zimbabwe | Men | Richest | No | 25+ | 198 | 21.04 | 17.61 | 24.94 |
| 2010 | Zimbabwe | Men | Richest | Yes | <25 | 116 | 84.93 | 76.76 | 90.58 |
| 2010 | Zimbabwe | Men | Richest | Yes | 25+ | 25 | 15.07 | 9.42 | 23.24 |
| 2010 | Zimbabwe | Women | Middle | No | <25 | 592 | 67.50 | 63.99 | 70.83 |
| 2010 | Zimbabwe | Women | Middle | No | 25+ | 270 | 32.50 | 29.17 | 36.01 |
| 2010 | Zimbabwe | Women | Middle | Yes | <25 | 190 | 69.71 | 63.27 | 75.45 |
| 2010 | Zimbabwe | Women | Middle | Yes | 25+ | 79 | 30.29 | 24.55 | 36.73 |
| 2010 | Zimbabwe | Women | Poorer | No | <25 | 627 | 71.73 | 68.45 | 74.79 |
| 2010 | Zimbabwe | Women | Poorer | No | 25+ | 236 | 28.27 | 25.21 | 31.55 |
| 2010 | Zimbabwe | Women | Poorer | Yes | <25 | 177 | 81.65 | 75.70 | 86.39 |
| 2010 | Zimbabwe | Women | Poorer | Yes | 25+ | 46 | 18.35 | 13.61 | 24.30 |
| 2010 | Zimbabwe | Women | Poorest | No | <25 | 762 | 78.69 | 75.71 | 81.40 |
| 2010 | Zimbabwe | Women | Poorest | No | 25+ | 193 | 21.31 | 18.60 | 24.29 |
| 2010 | Zimbabwe | Women | Poorest | Yes | <25 | 222 | 83.12 | 77.76 | 87.40 |
| 2010 | Zimbabwe | Women | Poorest | Yes | 25+ | 45 | 16.88 | 12.60 | 22.24 |
| 2010 | Zimbabwe | Women | Richer | No | <25 | 640 | 59.04 | 55.82 | 62.19 |
| 2010 | Zimbabwe | Women | Richer | No | 25+ | 422 | 40.96 | 37.81 | 44.18 |
| 2010 | Zimbabwe | Women | Richer | Yes | <25 | 211 | 64.94 | 59.34 | 70.16 |
| 2010 | Zimbabwe | Women | Richer | Yes | 25+ | 111 | 35.06 | 29.84 | 40.66 |
| 2010 | Zimbabwe | Women | Richest | No | <25 | 632 | 53.25 | 49.87 | 56.60 |
| 2010 | Zimbabwe | Women | Richest | No | 25+ | 530 | 46.75 | 43.40 | 50.13 |
| 2010 | Zimbabwe | Women | Richest | Yes | <25 | 140 | 53.44 | 46.26 | 60.49 |
| 2010 | Zimbabwe | Women | Richest | Yes | 25+ | 115 | 46.56 | 39.51 | 53.74 |
| 2015 | Zimbabwe | Men | Middle | No | <25 | 821 | 92.32 | 90.35 | 93.92 |
| 2015 | Zimbabwe | Men | Middle | No | 25+ | 77 | 7.68 | 6.08 | 9.65 |
| 2015 | Zimbabwe | Men | Middle | Yes | <25 | 128 | 94.09 | 88.61 | 97.02 |
| 2015 | Zimbabwe | Men | Middle | Yes | 25+ | 9 | 5.91 | 2.98 | 11.39 |
| 2015 | Zimbabwe | Men | Poorer | No | <25 | 792 | 95.06 | 93.23 | 96.42 |
| 2015 | Zimbabwe | Men | Poorer | No | 25+ | 42 | 4.94 | 3.58 | 6.77 |
| 2015 | Zimbabwe | Men | Poorer | Yes | <25 | 142 | 95.05 | 89.19 | 97.81 |
| 2015 | Zimbabwe | Men | Poorer | Yes | 25+ | 6 | 4.95 | 2.19 | 10.81 |
| 2015 | Zimbabwe | Men | Poorest | No | <25 | 718 | 94.57 | 92.57 | 96.06 |
| 2015 | Zimbabwe | Men | Poorest | No | 25+ | 38 | 5.43 | 3.94 | 7.43 |
| 2015 | Zimbabwe | Men | Poorest | Yes | <25 | 132 | 96.60 | 91.70 | 98.65 |
| 2015 | Zimbabwe | Men | Poorest | Yes | 25+ | 5 | 3.40 | 1.35 | 8.30 |
| 2015 | Zimbabwe | Men | Richer | No | <25 | 1122 | 83.70 | 81.10 | 86.01 |
| 2015 | Zimbabwe | Men | Richer | No | 25+ | 230 | 16.30 | 13.99 | 18.90 |
| 2015 | Zimbabwe | Men | Richer | Yes | <25 | 194 | 88.53 | 82.35 | 92.73 |
| 2015 | Zimbabwe | Men | Richer | Yes | 25+ | 25 | 11.47 | 7.27 | 17.65 |
| 2015 | Zimbabwe | Men | Richest | No | <25 | 1061 | 70.34 | 66.78 | 73.68 |
| 2015 | Zimbabwe | Men | Richest | No | 25+ | 443 | 29.66 | 26.32 | 33.22 |
| 2015 | Zimbabwe | Men | Richest | Yes | <25 | 162 | 73.87 | 65.84 | 80.57 |
| 2015 | Zimbabwe | Men | Richest | Yes | 25+ | 52 | 26.13 | 19.43 | 34.16 |
| 2015 | Zimbabwe | Women | Middle | No | <25 | 533 | 61.39 | 58.12 | 64.56 |
| 2015 | Zimbabwe | Women | Middle | No | 25+ | 344 | 38.61 | 35.44 | 41.88 |
| 2015 | Zimbabwe | Women | Middle | Yes | <25 | 185 | 70.10 | 62.88 | 76.45 |
| 2015 | Zimbabwe | Women | Middle | Yes | 25+ | 73 | 29.90 | 23.55 | 37.12 |
| 2015 | Zimbabwe | Women | Poorer | No | <25 | 585 | 69.96 | 66.69 | 73.03 |
| 2015 | Zimbabwe | Women | Poorer | No | 25+ | 259 | 30.04 | 26.97 | 33.31 |
| 2015 | Zimbabwe | Women | Poorer | Yes | <25 | 153 | 79.55 | 72.43 | 85.20 |
| 2015 | Zimbabwe | Women | Poorer | Yes | 25+ | 41 | 20.45 | 14.80 | 27.57 |
| 2015 | Zimbabwe | Women | Poorest | No | <25 | 642 | 76.78 | 73.68 | 79.61 |
| 2015 | Zimbabwe | Women | Poorest | No | 25+ | 193 | 23.22 | 20.39 | 26.32 |
| 2015 | Zimbabwe | Women | Poorest | Yes | <25 | 200 | 80.54 | 74.59 | 85.37 |
| 2015 | Zimbabwe | Women | Poorest | Yes | 25+ | 48 | 19.46 | 14.63 | 25.41 |
| 2015 | Zimbabwe | Women | Richer | No | <25 | 781 | 53.28 | 50.00 | 56.54 |
| 2015 | Zimbabwe | Women | Richer | No | 25+ | 704 | 46.72 | 43.46 | 50.00 |
| 2015 | Zimbabwe | Women | Richer | Yes | <25 | 269 | 63.04 | 55.88 | 69.67 |
| 2015 | Zimbabwe | Women | Richer | Yes | 25+ | 164 | 36.96 | 30.33 | 44.12 |
| 2015 | Zimbabwe | Women | Richest | No | <25 | 840 | 47.16 | 44.25 | 50.08 |
| 2015 | Zimbabwe | Women | Richest | No | 25+ | 943 | 52.84 | 49.92 | 55.75 |
| 2015 | Zimbabwe | Women | Richest | Yes | <25 | 164 | 47.19 | 39.93 | 54.58 |
| 2015 | Zimbabwe | Women | Richest | Yes | 25+ | 163 | 52.81 | 45.42 | 60.07 |

SES: socioeconomic status.

# **Table S in S1 text. Multilevel association between HIV status (independent variable) and body mass index as well as overweight (dependent variable), adjusted by socioeconomic status, age, and sex, overall and stratified by study period (early vs late surveys).**

| **Period** | **Predictors** | **β / Odds Ratio (95% Confidence Interval) adjusted by age and sex** |
| --- | --- | --- |
| **Outcome: body mass index** | | |
| Overall | HIV (no [ref] vs yes) | -0.73 (-0.79; -0.65) |
|  | Socioeconomic status (poorest [ref]) |  |
|  | Poorer | 0.34 (0.29; 0.40) |
|  | Middle | 0.69 (0.63; 0.75) |
|  | Richer | 1.42 (1.37; 1.48) |
|  | Richest | 2.93 (2.87; 2.98) |
|  | Age (years) | 0.07 (0.07; 0.08) |
|  | Sex (men [ref]) |  |
|  | Women | 1.97 (1.92; 2.01) |
| Early | HIV (no [ref] vs yes) | -0.65 (-0.77; -0.54) |
|  | Socioeconomic status (poorest [ref]) |  |
|  | Poorer | 0.20 (0.11; 0.30) |
|  | Middle | 0.55 (0.46; 0.65) |
|  | Richer | 1.08 (0.99; 1.18) |
|  | Richest | 2.50 (2.41; 2.59) |
|  | Age (years) | 0.06 (0.06; 0.07) |
|  | Sex (men [ref]) |  |
|  | Women | 1.63 (0.19; 3.08) |
| Later | HIV (no [ref] vs yes) | -0.85 (-0.97; -0.75) |
|  | Socioeconomic status (poorest [ref]) |  |
|  | Poorer | 0.37 (0.28; 0.46) |
|  | Middle | 0.74 (0.65; 0.83) |
|  | Richer | 1.60 (1.51; 1.69) |
|  | Richest | 3.14 (3.06; 3.22) |
|  | Age (years) | 0.08 (0.07; 0.08) |
|  | Sex (men [ref]) |  |
|  | Women | 2.17 (2.10; 2.24) |
| **Outcome: overweight (no [ref] versus yes)** | | |
| Overall | HIV (no [ref] vs yes) | 0.69 (0.66; 0.73) |
|  | Socioeconomic status (poorest [ref]) |  |
|  | Poorer | 1.31 (1.24; 1.38) |
|  | Middle | 1.72 (1.63; 1.81) |
|  | Richer | 2.93 (1.79; 3.08) |
|  | Richest | 6.80 (6.49; 7.12) |
|  | Age (years) | 1.06 (1.06; 1.06) |
|  | Sex (men [ref]) |  |
|  | Women | 4.63 (4.45; 4.83) |
| Early | HIV (no [ref] vs yes) | 0.72 (0.66; 0.78) |
|  | Socioeconomic status (poorest [ref]) |  |
|  | Poorer | 1.18 (1.07; 1.31) |
|  | Middle | 1.58 (1.43; 1.74) |
|  | Richer | 2.62 (2.39; 2.88) |
|  | Richest | 6.47 (5.94; 7.06) |
|  | Age (years) | 1.06 (1.06; 1.06) |
|  | Sex (men [ref]) |  |
|  | Women | 3.48 (1.51; 8.04) |
| Later | HIV (no [ref] vs yes) | 0.65 (0.61; 0.70) |
|  | Socioeconomic status (poorest [ref]) |  |
|  | Poorer | 1.26 (1.16; 1.36) |
|  | Middle | 1.74 (1.61; 1.88) |
|  | Richer | 3.09 (2.88; 3.32) |
|  | Richest | 7.12 (6.66; 7.62) |
|  | Age (years) | 1.06 (1.06; 1.06) |
|  | Sex (men [ref]) |  |
|  | Women | 4.77 (4.51; 5.04) |

# **Table T in S1 text. Multilevel association between HIV status (independent variable) and body mass index as well as overweight (dependent variable), adjusted by age and sex, stratified by study period (early vs late surveys), overall and for each level of socioeconomic status.**

| **Period** | **Socioeconomic Stratification** | **Odds Ratio / β (95% Confidence Interval) adjusted by age and sex** |
| --- | --- | --- |
| **Outcome: overweight (no [ref] versus yes)** | | |
| Overall | Overall | 0.77 (0.74-0.81) |
|  | Poorest | 0.82 (0.71-0.94) |
|  | Poorer | 0.73 (0.64-0.83) |
|  | Middle | 0.61 (0.54-0.68) |
|  | Richer | 0.64 (0.58-0.70) |
|  | Richest | 0.67 (0.62-0.72) |
| Early | Overall | 0.79 (0.73-0.86) |
|  | Poorest | 0.83 (0.63-1.10) |
|  | Poorer | 0.84 (0.67-1.06) |
|  | Middle | 0.54 (0.43-0.68) |
|  | Richer | 0.70 (0.58-0.82) |
|  | Richest | 0.70 (0.61-0.81) |
| Later | Overall | 0.73 (0.68-0.78) |
|  | Poorest | 0.84 (0.68-1.05) |
|  | Poorer | 0.60 (0.48-0.75) |
|  | Middle | 0.64 (0.53-0.77) |
|  | Richer | 0.55 (0.48-0.64) |
|  | Richest | 0.64 (0.57-0.71) |
| **Outcome: BMI** | | |
| Overall | Overall | -0.54 (-0.61; -0.47) |
|  | Poorest | -0.42 (-0.57; -0.28) |
|  | Poorer | -0.49 (-0.64; -0.34) |
|  | Middle | -0.70 (-0.86; -0.55) |
|  | Richer | -0.84 (-0.99; -0.69) |
|  | Richest | -1.14 (-1.29; -0.99) |
| Early | Overall | -0.51 (-0.63; -0.39) |
|  | Poorest | -0.36 (-0.61; -0.12) |
|  | Poorer | -0.58 (-0.81; -0.35) |
|  | Middle | -0.94 (-1.19; -0.69) |
|  | Richer | -0.63 (-0.88; -0.38) |
|  | Richest | -0.87 (-1.12; -0.62) |
| Later | Overall | -0.69 (-0.81; -0.58) |
|  | Poorest | -0.48 (-0.70; -0.25) |
|  | Poorer | -0.55 (-0.79; -0.30) |
|  | Middle | -0.63 (-0.87; -0.39) |
|  | Richer | -1.18 (-1.42; -0.94) |
|  | Richest | -1.34 (-1.58; -1.11) |

# **Fig A in S1 text. Age-SES standardized prevalence of overweight (%) by period, sex, and HIV status.**


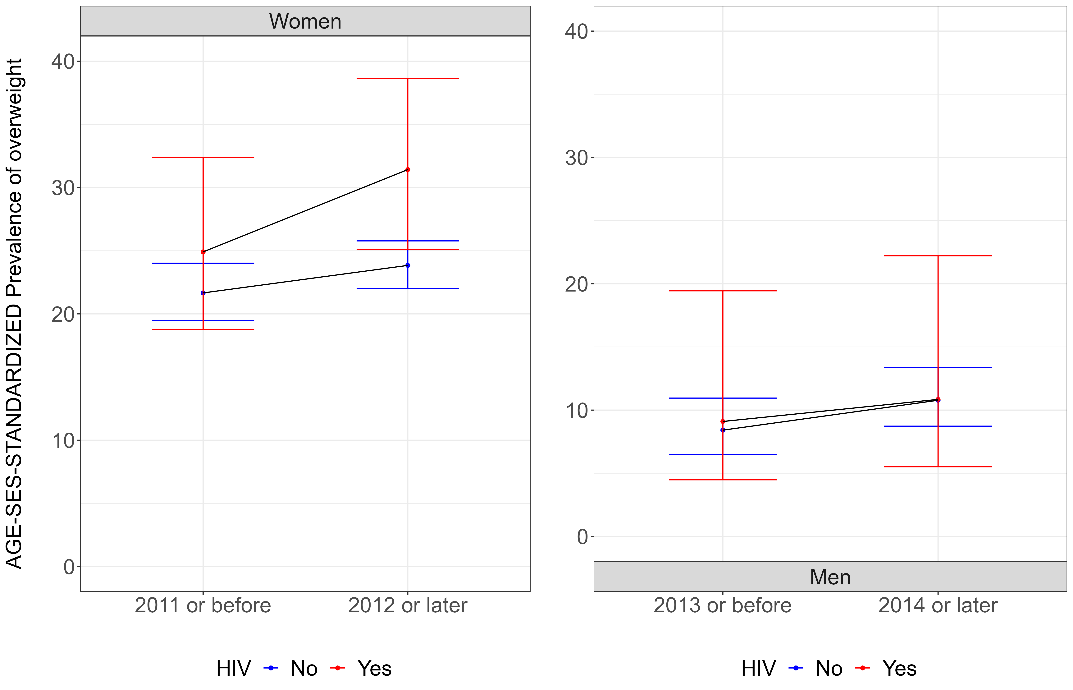


Overweight as body mass index ≥25 kg/m^2^. Results account for the complex survey design of each DHS. Results without age-SES standardization are available in Supplementary Table 10. Underlying results are available in Supplementary Table 11. For a list of DHS included in the earliest (before 2011 for women and before 2014 for men) and latest (after 2011 for women and after 2011 for men) periods, please refer to Supplementary Table 5. SES: socioeconomic status.

# **Fig B in S1 text. Age-SES standardized prevalence (%) of overweight stratified by sex and survey.**


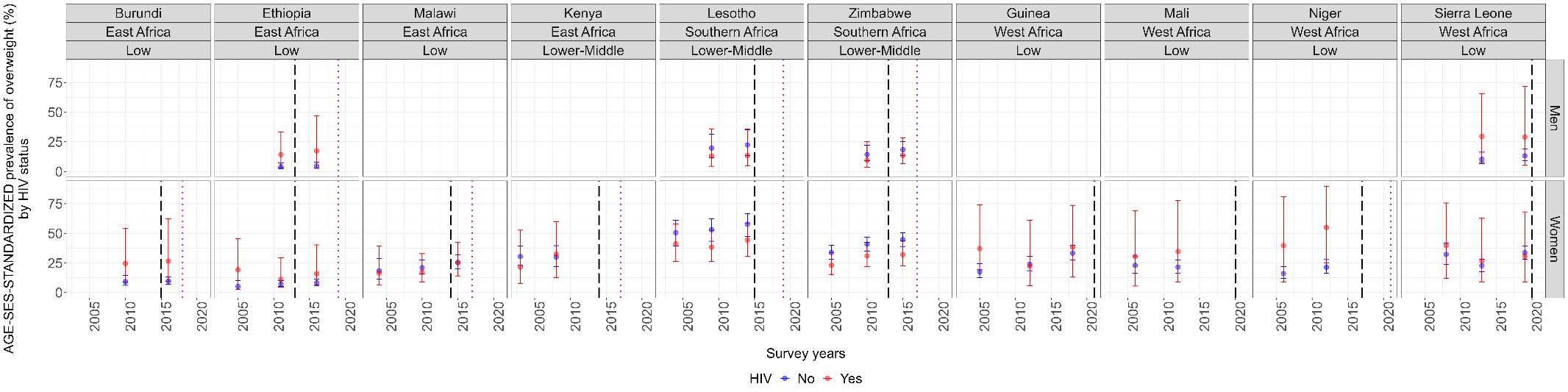


Overweight as body mass index ≥25 kg/m^2^. Results account for the complex survey design of each DHS. The vertical black dashed line signals the year when antiretroviral treatment (ART) coverage reached 50% of the population. The vertical purple dotted line signals the year when the ART coverage reached 75% of the population. The crude estimates (i.e., not age-SES standardized) are available in Supplementary Table 14. The underlying estimates are available in Supplementary Table 15. SES: socioeconomic status.

# **Fig C in S1 text. Meta-regression of prevalence (%) of overweight, as well as difference in prevalence of overweight between people without and with HIV, on antiretroviral treatment coverage.**


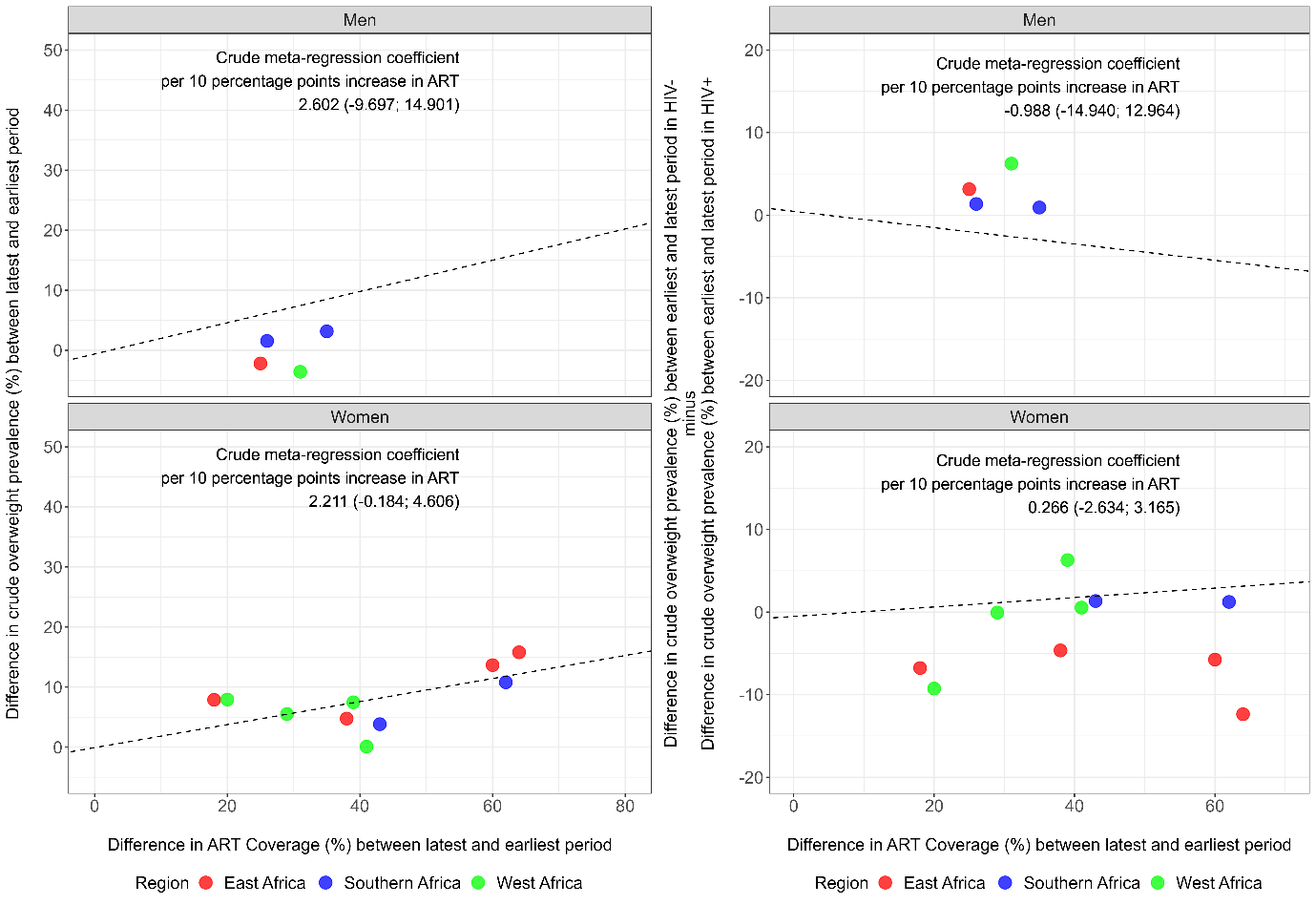


Overweight as body mass index ≥25 kg/m^2^. Each dot is a DHS. Colors depict the regions. we plotted in the y-axis a difference in difference: difference in mean BMI between the latest and earliest periods in PLWoH *minus* difference in mean BMI between the latest and earliest periods in PLWH. On the x-axis, we plotted the difference in ART coverage between the latest and earliest periods. The exposure was ART coverage by 10 percentage points to make the regression coefficients meaningful, i.e., observed prevalence of ART coverage divided by 10. Both the outcome and the exposure were at the country level and in the same calendar year. The meta-regression used the crude mean estimates (not age-SES standardized). Figure shows the coefficients for the crude meta-regression, for coefficients of the other meta-regression model and the 95% confidence intervals, please refer to Supplementary Table 16.

# **Fig D in S1 text. Mean body mass index throughout the observation period by socioeconomic quintile and HIV status in men; not age-SES standardized.**

**
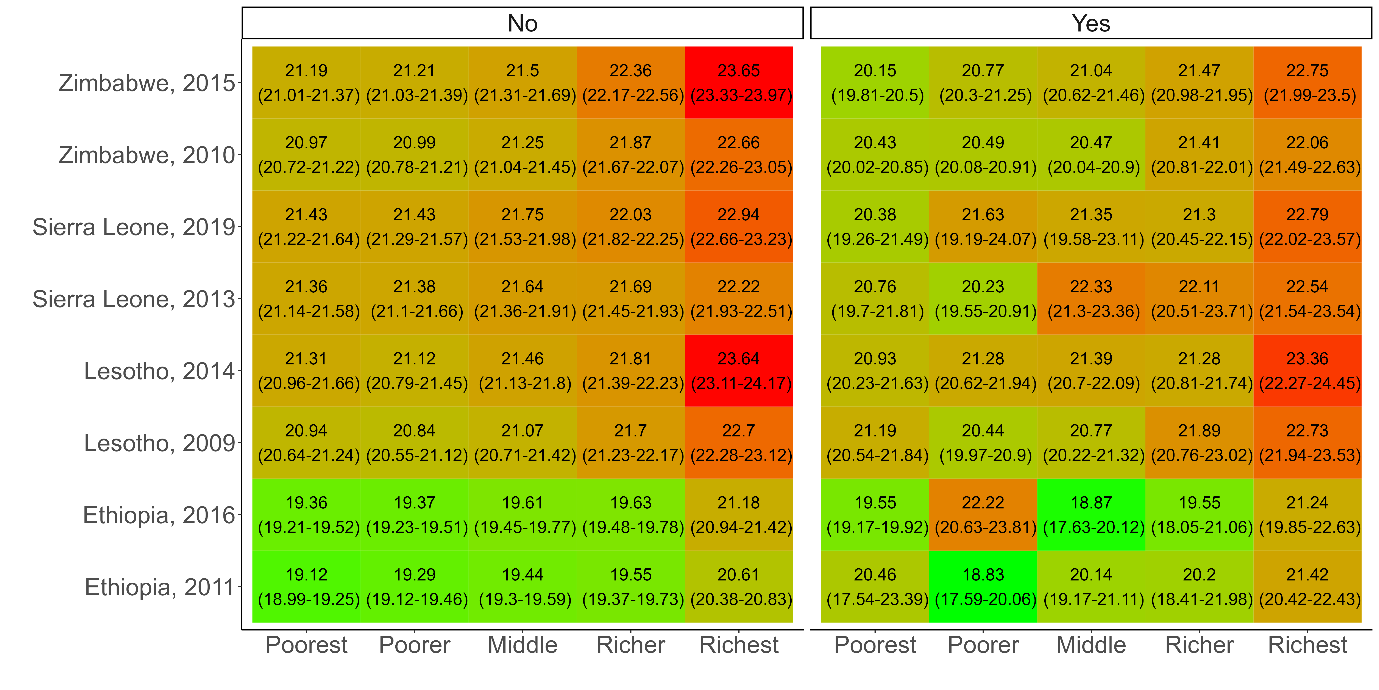
**

# **Fig E in S1 text. Mean body mass index throughout the observation period by socioeconomic quintile and HIV status in women; not age-SES standardized.**

**
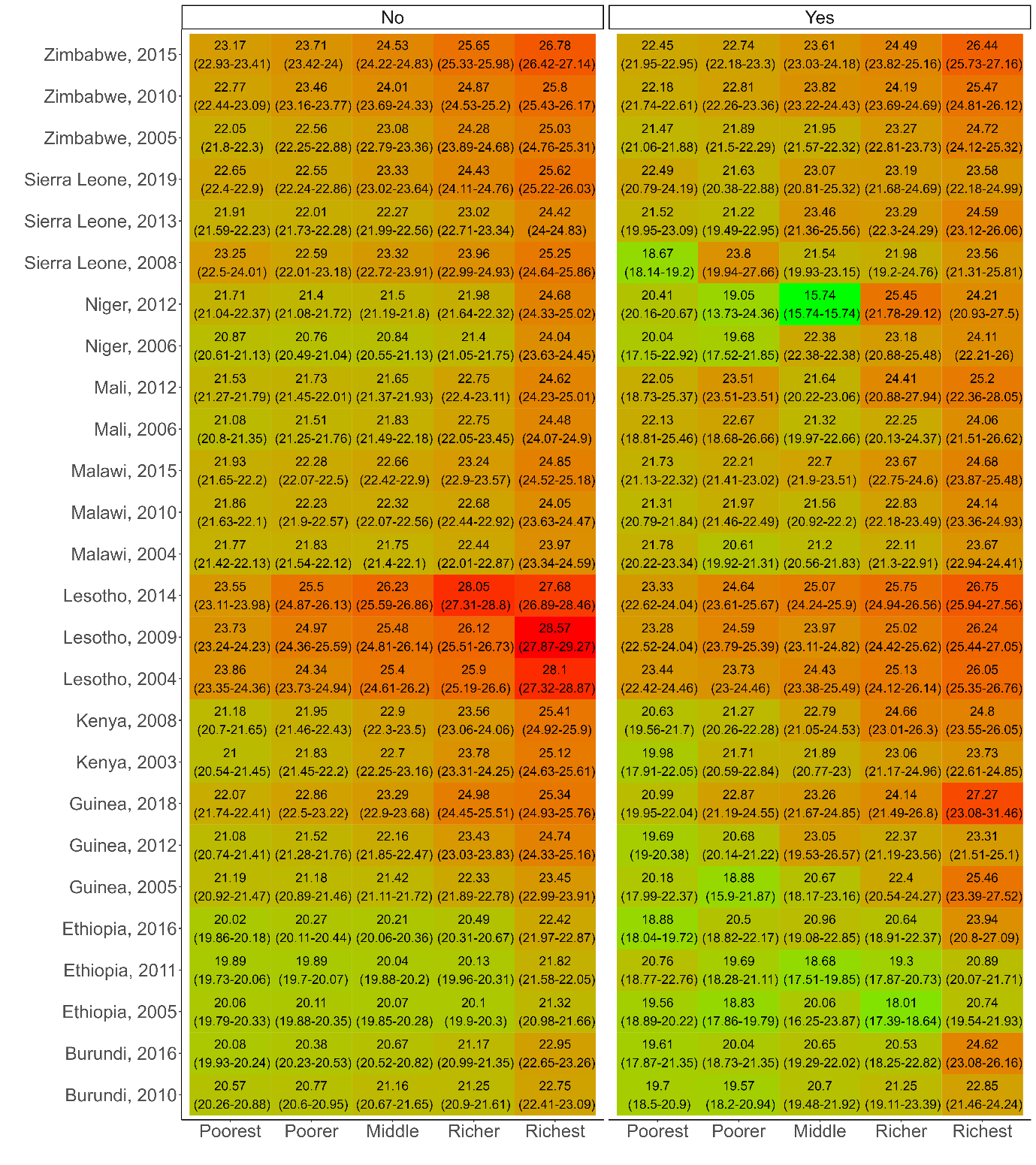
**

# **Fig F in S1 text. Prevalence of overweight throughout the observation period by socioeconomic quintile and HIV status in men; not age-SES standardized.**

**
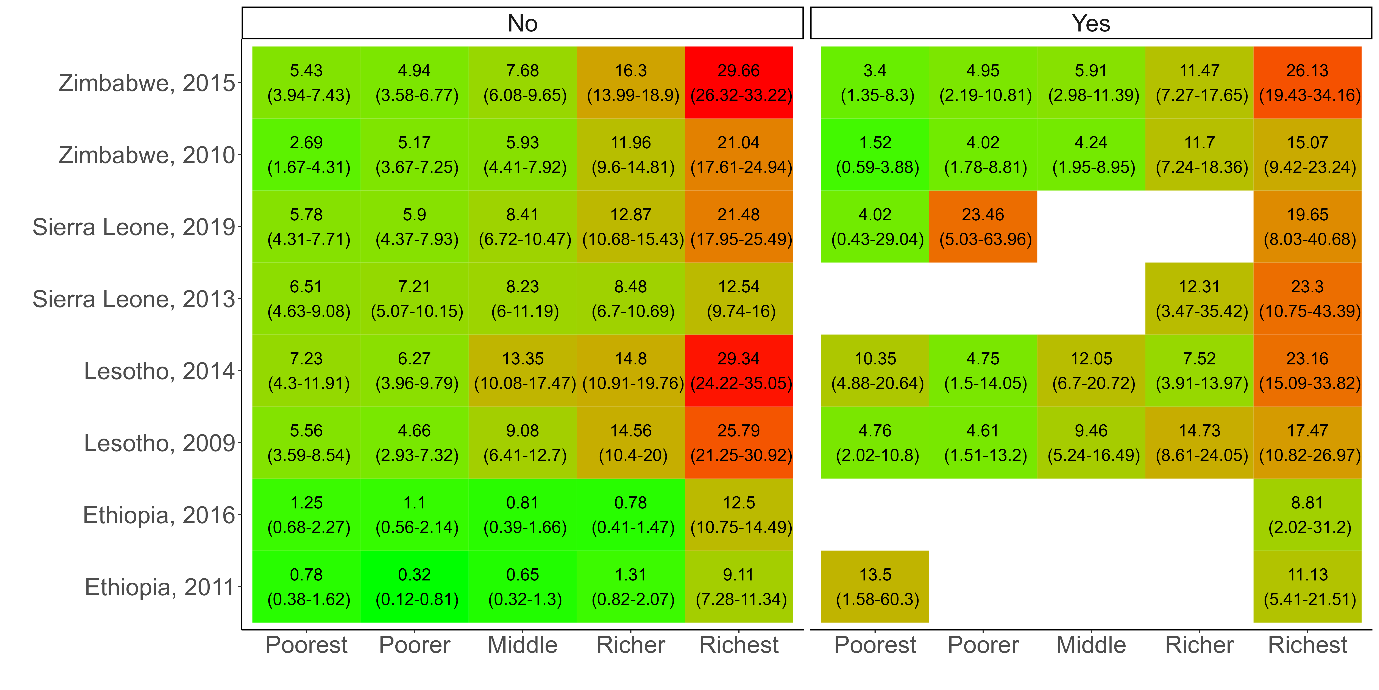
**

# **Fig G in S1 text. Prevalence of overweight throughout the observation period by socioeconomic quintile and HIV status in women; not age-SES standardized.**


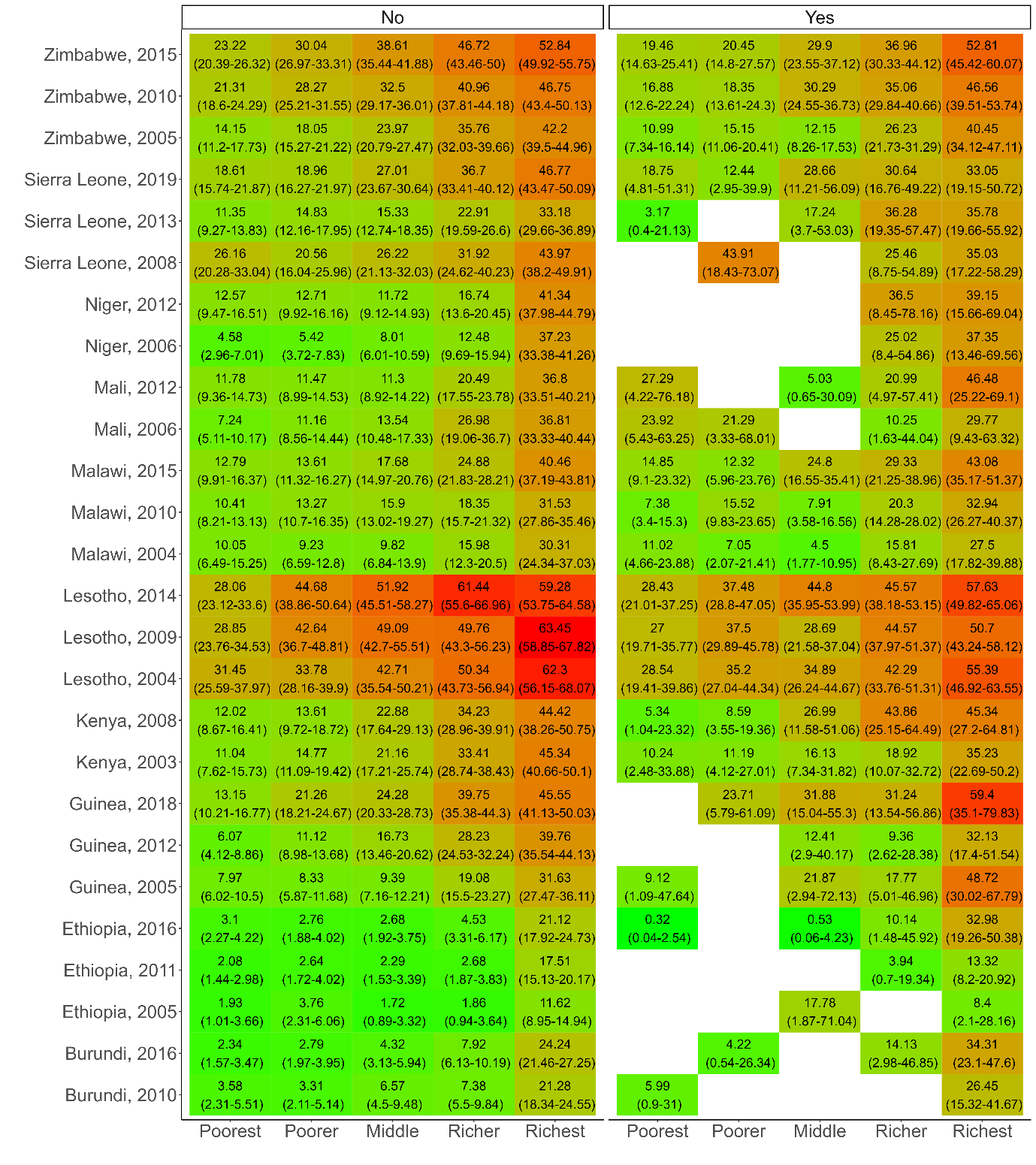


# **Fig H in S1 text. Comparison of crude and age-standardized mean body mass index by region and sex.**


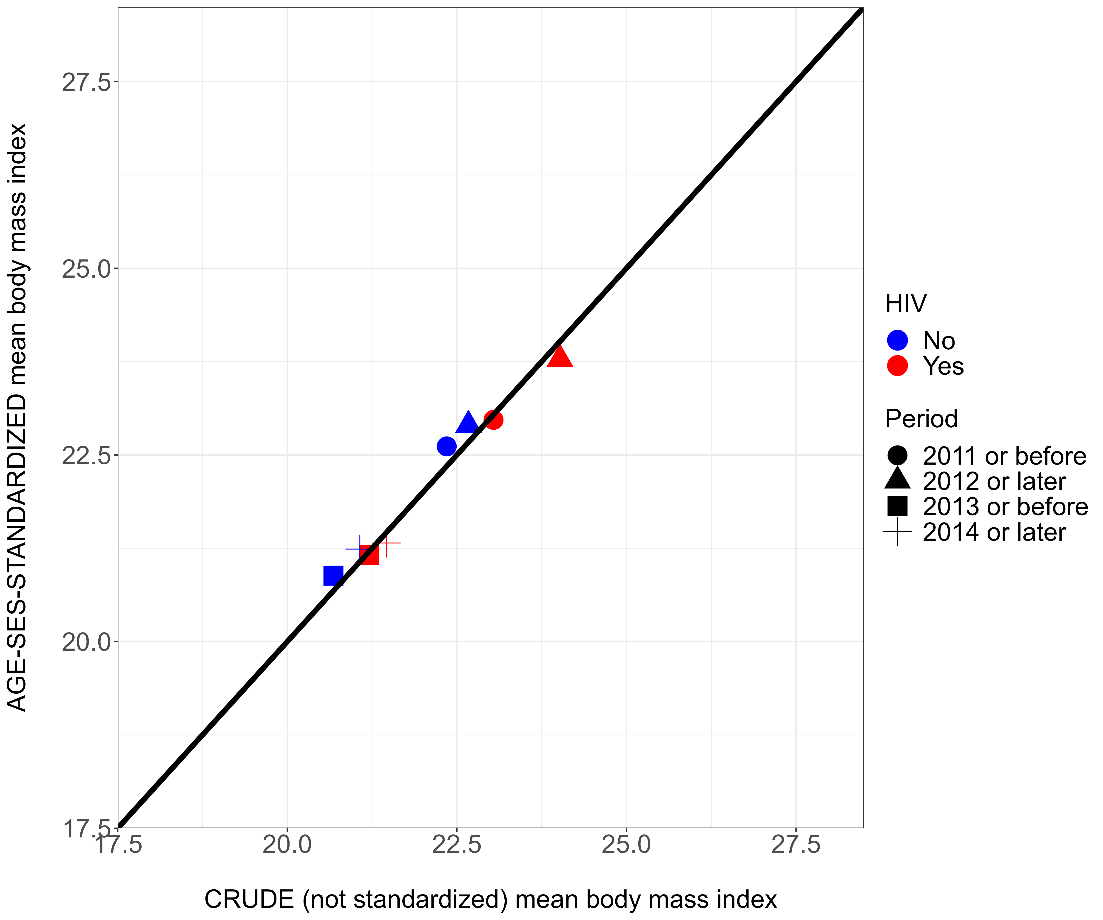


# **References**

1. Ahmad OB, Boschi-Pinto C, Lopez AD, Murray CJ, Lozano R, Inoue M. Age standardization of rates: a new WHO standard. World Health Organization, Geneva. 2001.

2. Burns RB, Dobson CB. Standard error of the difference between means. Experimental Psychology: Research Methods and Statistics. Dordrecht: Springer Netherlands; 1981: 151-7.
